# Alkyl deoxyglycoside-polymyxin combinations against critical priority carbapenem-resistant gram-negative bacteria

**DOI:** 10.1038/s41598-024-51428-6

**Published:** 2024-01-26

**Authors:** Ana M. de Matos, Patrícia Calado, Mónica Miranda, Rita Almeida, Amélia P. Rauter, M. Conceição Oliveira, Vera Manageiro, Manuela Caniça

**Affiliations:** 1 Centro de Química Estrutural, Institute of Molecular Sciences, Departmento de Química e Bioquímica, Faculdade de Ciências, Universidade de Lisboa, 1749-016 Campo Grande, Lisbon, Portugal; 2 Centro de Química Estrutural, Institute of Molecular Sciences, Instituto Superior Técnico, Av. Rovisco Pais, 1049-001 Lisbon, Portugal; 3 National Reference Laboratory of Antibiotic Resistances and Healthcare-Associated Infections, Department of Infectious Diseases, National Institute of Health Dr. Ricardo Jorge, 1649-016 Lisbon, Portugal; 4 Centre for the Studies of Animal Science, Institute of Agrarian and Agri-Food Sciences and Technologies, University of Porto, Porto, Portugal; 5 AL4AnimalS, Associate Laboratory for Animal and Veterinary Sciences, Lisbon, Portugal; 6 CIISA, Center for Interdisciplinary Research in Animal Health, Faculty of Veterinary Medicine, University of Lisbon, Lisbon, Portugal

**Keywords:** Drug discovery, Microbiology, Infectious diseases, Medicinal chemistry, Organic chemistry, Chemical synthesis

## Abstract

The escalating antimicrobial resistance crisis urges the development of new antibacterial treatments with innovative mechanisms of action, particularly against the critical priority carbapenem-resistant *Acinetobacter baumannii* (CRAB), *Pseudomonas aeruginosa* (CRPA) and *Enterobacteriaceae* (CRE). Membrane-disrupting dodecyl deoxyglycosides have been reported for their interesting phosphatidylethanolamine-associated bactericidal activity against Gram-positive strains; however, their inability to penetrate the Gram-negative outer membrane (OM) renders them useless against the most challenging pathogens. Aiming to repurpose alkyl deoxyglycosides against Gram-negative bacteria, this study investigates the antimicrobial effects of five reference compounds with different deoxygenation patterns or anomeric configurations in combination with polymyxins as adjuvants for enhanced OM permeability. The generation of the lead 4,6-dideoxy scaffold was optimized through a simultaneous dideoxygenation step and applied to the synthesis of a novel alkyl 4,6-dideoxy *C*-glycoside **5**, herein reported for the first time. When combined with subtherapeutic colistin concentrations, most glycosides demonstrated potent antimicrobial activity against several multidrug-resistant clinical isolates of CRAB, CRE and CRPA exhibiting distinct carbapenem resistance mechanisms, together with acceptable cytotoxicity against human HEK-293T and Caco-2 cells. The novel 4,6-dideoxy *C*-glycoside **5** emerged as the most promising prototype structure for further development (MIC 3.1 μg/mL when combined with colistin 0.5 μg/mL against CRPA or 0.25 μg/mL against several CRE and CRAB strains), highlighting the potential of *C*-glycosylation for an improved bioactive profile. This study is the first to show the potential of IM-targeting carbohydrate-based compounds for the treatment of infections caused by MDR Gram-negative pathogens of clinical importance.

## Introduction

Also referred to as the silent pandemic, antimicrobial resistance (AMR) is one of the most alarming threats posed to human health in the twenty-first century^1^. In 2019 alone, 4.95 million deaths were associated with bacterial AMR, of which 1.27 million were directly attributable to infections caused by antimicrobial-resistant bacteria^2^. The Gram-negative ESKAPE pathogens *Escherichia coli*, *Klebsiella pneumoniae*, *Acinetobacter baumannii,* and *Pseudomonas aeruginosa* are among the top six bacteria responsible for these estimates^2,3^. In agreement, carbapenem-resistant *A. baumannii* (CRAB), *P. aeruginosa* (CRPA), and *Enterobacteriaceae* (CRE) were placed at the very top of the World Health Organization (WHO)’s list of priority pathogens under the “critical priority” category. The fact that only one broad-spectrum agent, cefidorocol, is currently available to treat infections caused by all three bacteria highlights the urgent need for new therapeutic options, preferably with novel mechanisms of action (MoA)^4^.

**Figure 1:**
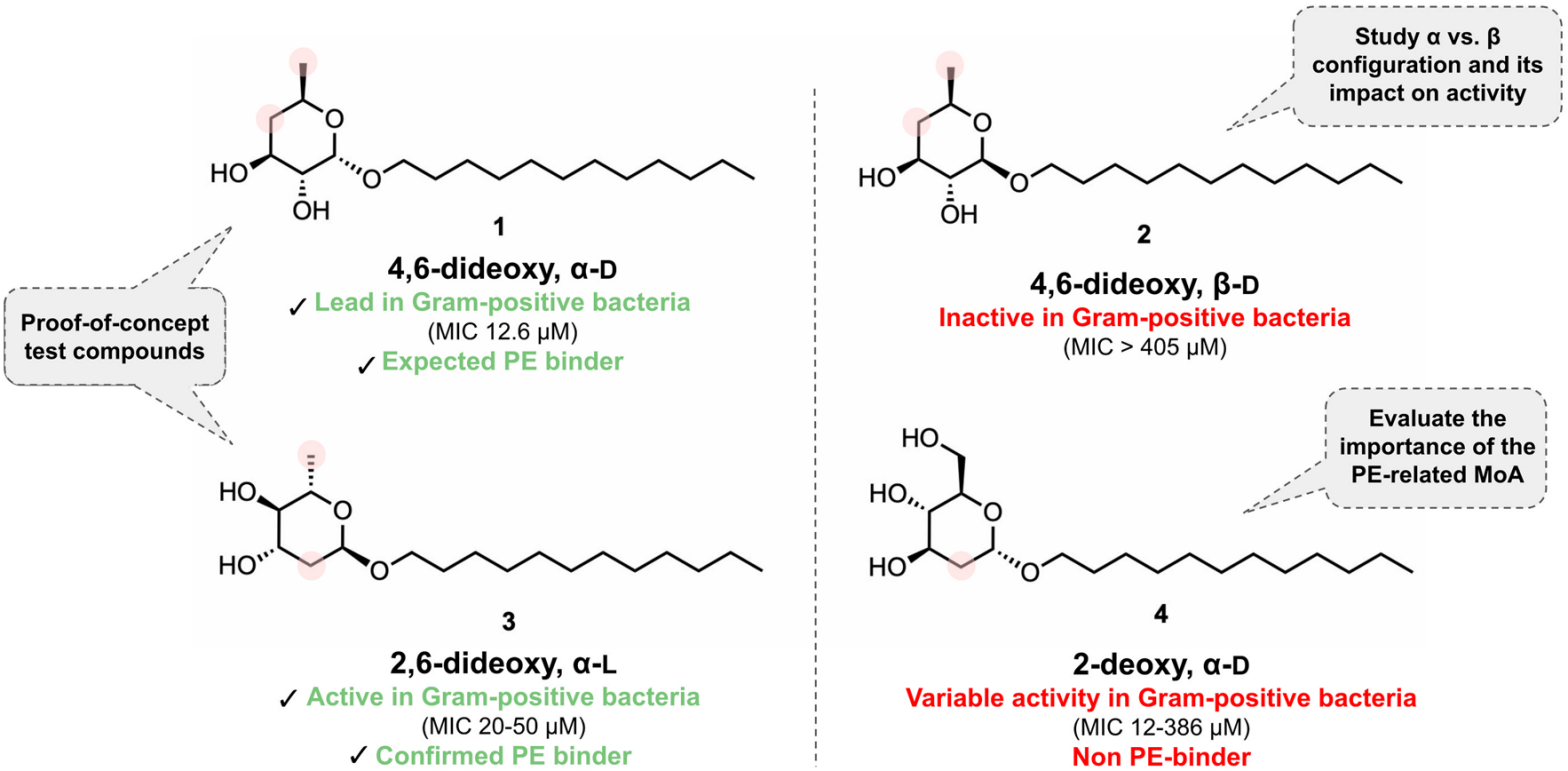
Structure of four dodecyl deoxyglycosides previously studied in Gram-positive bacteria^6^, and their role in the present work. Pink circles represent the locations where carbohydrate deoxygenation took place.

Carbohydrates are increasingly recognized as valuable scaffolds for the rational design and development of new compounds with antibiotic activity and innovative MoA^5^. In 2018, Rauter and co-workers reported a series of membrane-disrupting dodecyl deoxyglycosides with potent inhibitory activity against a small panel of Gram-positive bacteria, namely *Bacillus anthracis* (Sterne, ovine and pathogenic forms), *Enterococcus faecalis* and *Bacillus cereus*^6^. With a minimum inhibitory concentration (MIC) of 12.6 μM against all tested pathogens, the 4,6-dideoxy α-D-glycoside **1** (Fig. 1) was the best in the series, whereas the corresponding β-anomer (**2**) was virtually inactive (MIC > 405 μM). The 2,6-dideoxy α-L-glycoside **3** (MIC 20–50 μM), chosen as the representative structure of this class of compounds, was found to selectively interact with phosphatidylethanolamine (PE), present in bacterial membranes, versus phosphatidylcholine (PC), present in eukaryotic ones. When tested against the Gram-negative *E. coli* K12, compound **3** was not active per se (MIC 256 μg/mL), despite the 80% of PE in its inner membrane (IM) composition vs. the 43% reported for the membrane of *B. cereus*^7^. Yet, in *E. coli* K12 spheroplasts, where only the IM is left intact, a dramatic increase in inhibitory activity was observed (MIC 4 μg/mL), suggesting a complete lack of outer membrane (OM) permeability for these glycosides, but a clear potential for use in PE-enriched membranes in conditions where the OM integrity is compromised^6^. Furthermore, likely because of the membrane-targeting MoA, compound **3** presented low susceptibility to common mechanisms of resistance against several antibiotics used in the clinical setting, including erythromycin, penicillin, vancomycin, ciprofloxacin, and tetracycline^6^, ultimately pointing towards a vast therapeutic potential of dodecyl deoxyglycosides against MDR bacteria—one that is yet to be explored.

Due to the presence of the lipopolysaccharide (LPS)-encompassing OM, Gram-negative bacteria are indeed less susceptible to antibiotic entrance compared to Gram-positive bacteria, in addition to being particularly prone to develop permeability-related AMR mechanisms^8^. Because of the disparity observed between IM and OM in terms of physicochemical characteristics, compounds that are able to pass through the IM are not likely to pass through the OM and vice-versa^9^. Therefore, creative solutions involving the concomitant use of more than one compound may often be required when it comes to the development of new antimicrobial strategies directed against Gram-negative bacteria, particularly when the goal is to repurpose antibiotics with demonstrated activity against Gram-positive organisms^10^. In this context, this study explores the potential of dodecyl deoxyglycoside leads against the critical priority Gram-negative pathogens CRAB, CRPA, and CRE, ultimately aiming to contribute to new solutions for the emerging AMR threat. We hypothesized that by using subtherapeutic concentrations of an adjuvant agent able to enhance OM permeability, our glycosides would then succeed at disrupting the IM and cause bacterial cell lysis through a concerted, membrane-exclusive MoA. Polymyxins B (PMB) and E (colistin) were chosen as adjuvant agents for a few reasons: (i) these are last-resort antibiotics approved for clinical use and listed among the critically important antibiotics for combating AMR^11,12^; (ii) they act by binding to the OM LPS lipids, thus increasing OM permeability, as required^10^; (iii) polymyxins and polymyxin analogs have been previously shown to potentiate the activity of several antibiotics^13–15^. However, while these antibiotics are important for combating antimicrobial resistance, they should be carefully considered due to their potential for neurotoxicity and nephrotoxicity. Fortunately, these adverse effects are generally dose-dependent and typically reversible when the drug is discontinued early^16^. Moreover, there is evidence indicating that colistin does not appear to influence clinical outcomes or mortality, whether used alone or in combination^17^.

**Figure 2:**
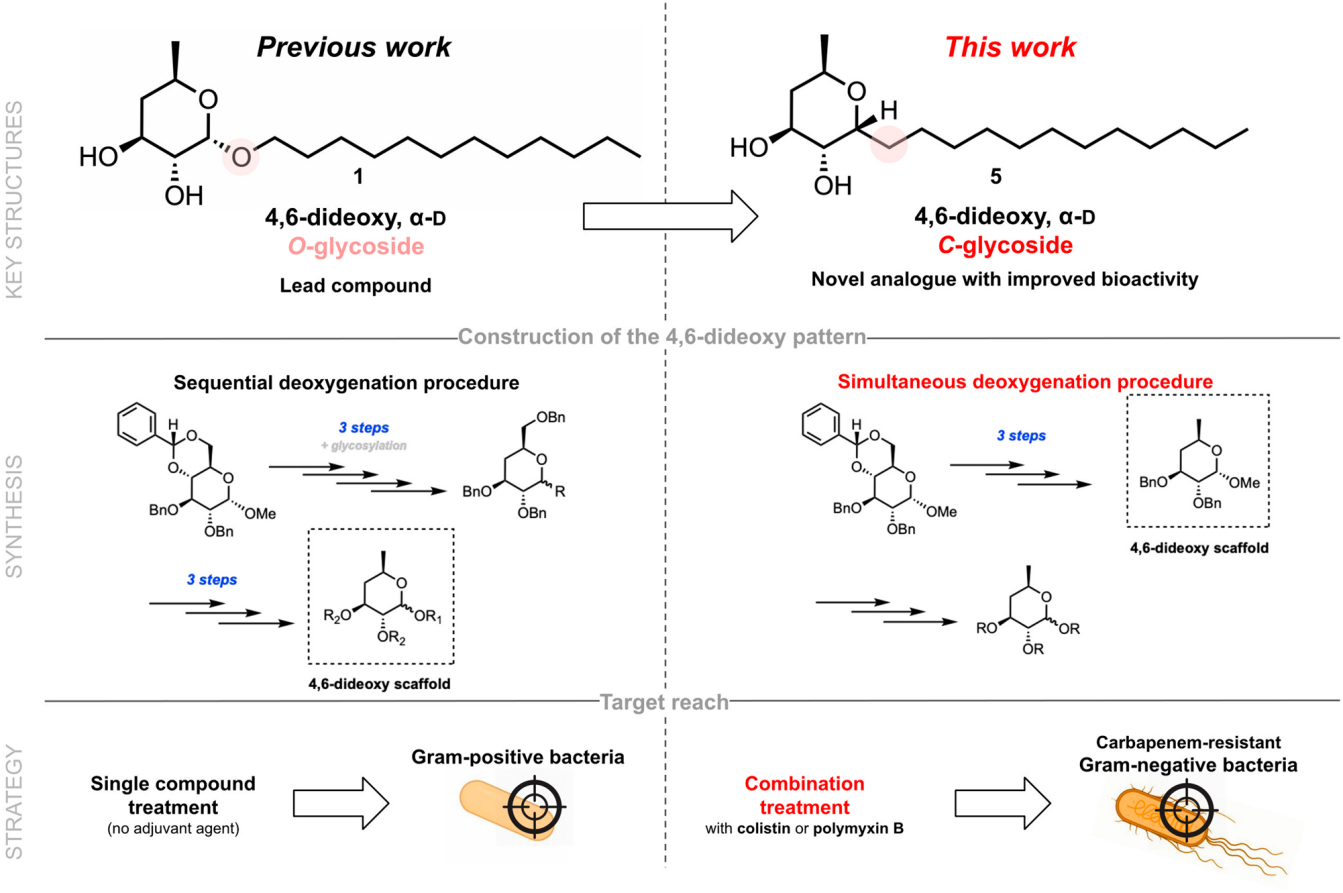
Strategy for the development and application of the novel *C*-glycoside analogue **5** (of **1**, from Fig. 1) in the present work. Pink circles highlight the implemented anomeric *O → C* substitution.

In addition to disclosing the lowest possible polymyxin concentration able to render the desired OM permeation, this study was also designed to identify the best glycoside among five reference structures for structural optimization. Besides the already described compounds **1** and **3**, 4,6-dideoxy β-D-glycoside **2** (Fig. 1) was chosen to provide a source of comparison between configuration α- vs. β- in *O*-glycosides against Gram-negative bacteria, since a huge disparity in activity between **1** and **2** has been reported in Gram-positive pathogens^6^. Furthermore, 2-deoxy α-D-glycoside **4** (Fig. 1), which exhibits limited affinity towards PE^6^, was also included to corroborate the importance of the PE-related MoA of alkyl deoxyglycosides in Gram-negative bacteria, as well as to highlight the role of the 6-deoxygenation pattern in activity. Last but not least, due to the potential for improved metabolic stability of *C*-glycosides versus their *O*-glycoside analogs^18^ and previous evidence of *C*-glycoside efficacy in carbohydrate-based antibiotic development^19,20^, we envisioned the anomeric *O → C* substitution in compound **1** to afford the more structurally robust α-glycoside **5** (Fig. 2), obtained through a simultaneous deoxygenation procedure and herein reported for the first time.

## Results and discussion

### Synthesis

**Figure 3:**
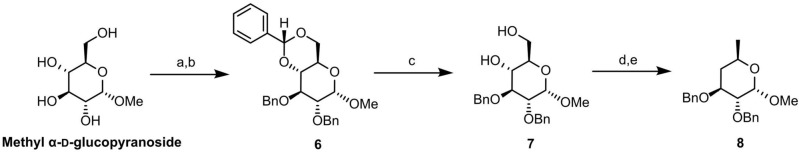
Synthesis of 4,6-dideoxy key intermediate **8** through a simultaneous dideoxygenation approach. Reagents and conditions: (**a**) PhCH(OMe)_2_, *p*-TsOH·H_2_O, ACN, 82 ºC, 19 h, 91%; (**b**) NaH, BnBr, DMF, 0ºC to rt, 17 h, 91%; (c) AcOH 80%, 50 ºC, 24 h, 98%; (d) Tf_2_O, Py, DCM, 0 ºC, 6 h; (e) Tol, *n*-Bu_4_NBH_4_, 85 ºC, 1h30, 88% over two steps.

Even though compounds **1** and **2** had previously been reported^6,21^, one of our goals was to further explore and optimize the construction of the desired 4,6-dideoxy pattern that was still offering significant potential for improvement. The synthesis started from the commercially available methyl α-D-glucopyranoside which, after 4,6-*O*-benzylidene introduction and subsequent benzylation of positions 2 and 3, rendered intermediate **6** in excellent yield (Fig. 3). In previous work (Fig. 2), selective benzylidene opening at position 4 in the presence of sodium cyanoborohydride and molecular iodine was the first step in the reported sequential deoxygenation procedure^21^. After triflate introduction followed by reduction of position 4, the sugar donor was immediately glycosylated. It was only after full benzyl removal that the last deoxygenation step was conducted, taking advantage of the preferential reactivity of position 6 when compared to positions 2 and 3. However, this procedure required a total of seven reaction steps from compound **6** before the 4,6-dideoxy pattern could be achieved^21^. In the present work, we opted for a full benzylidene deprotection in mildly acidic conditions at 50 ºC, which afforded intermediate **7** in almost quantitative yield (Fig. 3). Then, simultaneous triflation of positions 4 and 6, followed by reduction with tetrabutylammonium borohydride at 85 ºC gave the key intermediate **8** in 88% yield over two steps. We were hence able to access the target 4,6-dideoxy pattern in only three reaction steps, rendering the synthetic route toward the sugar donor for both *O*- and *C*-glycosylation much more concise, straightforward, and efficient.

**Figure 4:**
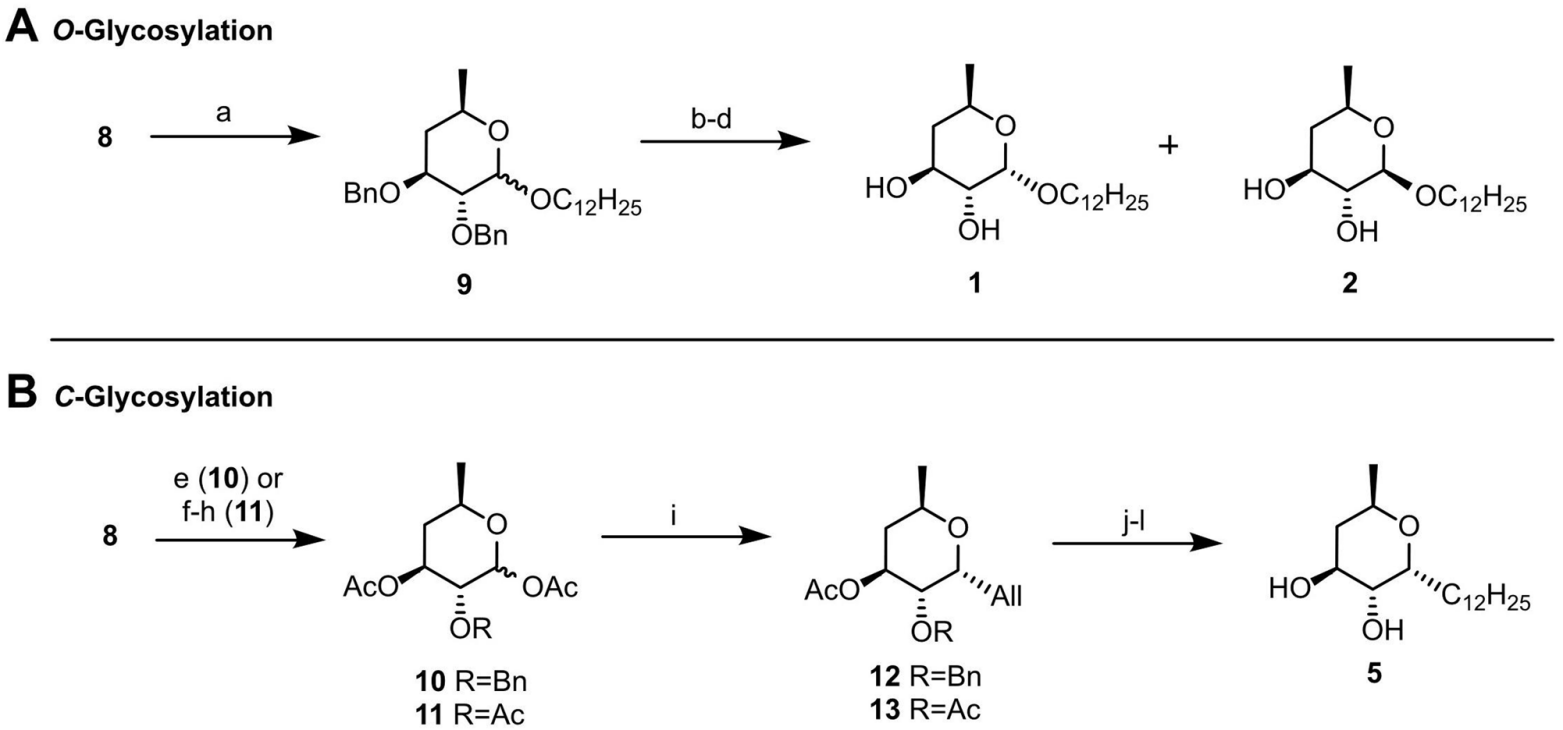
(**A**) *O-* and (**B**) *C*-glycosylation reactions towards the synthesis of compounds **1**, **2** and **5**. Reagents and conditions: (**A**) **(a)** dodecan-1-ol, Ambertlyst 15, DCM, 40 ºC, 40 h, 93%; (**b)** Pd/C, EtSiH, MeOH, rt; **(c)** py, DMAP, Ac_2_O, rt, 4 h; (**d)** NaOMe, MeOH, rt, 1–2 h, 37% (compound **1**) and 26% over three steps (compound** 2**); (**B**) (**e)** Ac_2_O/AcOH (1:1), H_2_SO_4_, 0 ºC, 20 h, 35% (compound **10**);(**f)** Pd/C, H_2_, MeOH/EtOAc 1:1, rt, 2h30; (**g)** Ac_2_O/AcOH (1:1), H_2_SO_4_, 0 ºC, o.n.; **(h)** Ac_2_O, py, rt, 1h45, 90% over three steps (compound **11**); (**i)** allyl trimethylsilane, BF_3_·OEt_2_, ACN, 0 ºC, 30 min-3 h, 91% (compound **12**) and 86% (compound **13**); (**j)** 2nd generation Hoveyda-Grubbs catalyst, undec-1-ene, DCM, 40 ºC, 24 h; (**k)** Pd/C, H_2_, EtOAc, rt, 6-20 h; **(l)** NaOMe, MeOH, rt, 1 h, 56% (from compound **12**) or 85% (from compound **13**) over three steps.

For the synthesis of compounds** 1** and **2**, intermediate **8** was *O*-glycosylated in the presence of dodecan-1-ol in acidic conditions, affording the inseparable anomeric mixture **9** in high yield (Fig. 4A). As previously described^21^, debenzylation still did not enable the separation of **1** and **2**. Therefore, the mixture was acetylated, the anomers separated by column chromatography, and Zemplén deacetylation gave the desired products in good overall yields. Compounds **1** and **2** were successfully accessed through this new procedure with only eight reaction steps, in 25% and 17% overall yield, respectively, compared with 11 steps in the previously described route, which gave **1** and **2** in 11% and 5% overall yield, respectively.

When it comes to *C*-glycosylation, intermediate **8** was first submitted to anomeric activation as previously described^6^. However, the use of strong acid in the attempted protocol for demethylation with concomitant acetylation led to a complex mixture of compounds, with **10** (Fig. 4B) being identified as the major product (35% yield) as a result of selective debenzylation and acetylation at position 3. After coming across such a low yield for a supposedly straightforward activation step, we decided to explore more efficient alternatives, starting with the full debenzylation of** 8** prior to anomeric activation. Thus, catalytic dehydrogenation followed by acid-promoted demethylation was conducted, after which full acetylation in the presence of acetic anhydride and pyridine gave compound **11** in 90% yield over three steps. At this point, it became clear that the switch from benzyl to acetate groups was a requirement for efficient anomeric activation. We, therefore, pondered the use of the 2,3-di-*O*-acetyl-4,6-*O*-benzylidene precursor **S1** (see Supplementary Fig. S1 online) as the starting material. Given the incompatibility of acetate protection with hydride-donor reagents such as the one used in the synthesis of **8**, an alternative route towards **11** relying on sequential iodination of positions 4 and 6 through the Garegg method^22^ followed by catalytic hydrogenation in the presence of a base^23^ was explored. Yet, due to the extent of this procedure compared to the above-described simultaneous 4,6-dideoxygenation, anomeric activation starting from the benzyl-protected intermediate **8** still proved to be more efficient.

For comparison purposes, *C*-glycosylation of both **10** and **11** was carried out in parallel. The use of boron trifluoride etherate in the presence of allyl trimethylsilane^6^ at 0 ºC gave the allyl α-*C*-glycosides **12** and **13**, respectively, in equally high yields (Fig. 4B). Then, a chain-elongation metathesis reaction catalysed by the 2nd Generation Grubbs-Hoveyda catalyst in the presence of undec-1-ene^6^ followed by catalytic hydrogenation and deacetylation afforded, in both cases, the final compound **5** in moderate and good yields, respectively. All in all, the synthesis of this new *C*-glycoside was found to be significantly more efficient through intermediate **11** (47% overall yield over 12 reaction steps) than through intermediate **10** (13% overall yield over 10 steps), regardless of the two additionally required reaction steps. Of note, compounds **3** and **4** were synthesized according to the previously described methodology^6^.

### Antibacterial activity

Glycosides and glycoside-adjuvant combinations were evaluated against two well-characterized Gram-negative strains for validation of our study: the antibiotic-susceptible *E. coli* ATCC 25922, and the colistin-resistant *E. coli* NCTC 13846 strains. We then set a panel of six carbapenem-resistant Gram-negative clinical isolates as test strains, all of which are part of the Portuguese National Reference Laboratory for Antibiotic Resistance and Healthcare-Associated Infections (NRL-AR/HAI, INSA) collection. These comprise several colistin-susceptible Gram-negative ESKAPE pathogens (*K. pneumoniae*, *E. cloacae*, *A. baumannii* and *P. aeruginosa*) expressing different types of carbapenem-hydrolyzing enzymes, including OXA-48, NDM-1, KPC-3, IMP-1 and GES-12. Most of these strains are also resistant to penicillins, cephalosporins, fluoroquinolones and aminoglycosides, with the complete antibiotic susceptibility and resistance profiles being presented in Supplementary Table S1 online. All test strains were susceptible to both colistin and PMB with a MIC value of 0.5 μg/mL or 1 μg/mL (*P. aeruginosa* CQ4924). Based on the reported concentration-dependent polymyxin-LPS electrostatic interactions leading to OM permeation^24–26^, we envisioned that polymyxin concentrations lower than the MIC would be sufficient to enhance OM permeability to our glycosides without causing significant inhibition in bacterial cell growth. Furthermore, subtherapeutic polymyxin concentrations have been described to enhance OM permeability in Gram-negative bacteria without interfering with IM integrity^27^. Therefore, the maximum polymyxin concentration for glycoside-polymyxin combination assays was established at the first two-fold dilution of the maximum MIC, i.e., 0.5 μg/mL. Of note, MIC values for the colistin-resistant control strain (*E. coli* NCTC 13846) were, as anticipated, significantly higher (4 μg/mL; Table 1) compared to the remaining bacteria, which should result in complete glycoside inactivity for the maximum used polymyxin concentration of 0.5 μg/mL.

**Table 1:** MIC (μg/mL) values for both polymyxins used in this study as adjuvant agents against several critical priority carbapenem-resistant Gram-negative clinical isolates. Results are presented as the median of three independent replicates. ^b^Acquired carbapenem- or colistin-resistance mechanisms.

Antibiotics	MIC (μg/mL)							
	A	B	C	D	E	F	G	H
	*E. coli* ATCC 25922	*E. coli* NCTC 13846	*K. pneumoniae* CQ4921	*E. cloacae CQ1941*	*K. pneumoniae* CQ1942	*K. pneumoniae* CQ1947	*A. baumannii* CQ4322	*P. aeruginosa* CQ4924
		(MCR-1)^a^	(OXA-48)^a^	(NDM-1)^a^	(KPC-3)^a^	(IMP-1)^a^	(GES-12)^a^	
Colistin	0.5	4	0.5	0.5	0.5	0.5	0.5	1
PMB	0.5	4	0.5	0.5	0.5	0.5	0.5	1

### Glycoside-colistin combinations

**Figure 5:**
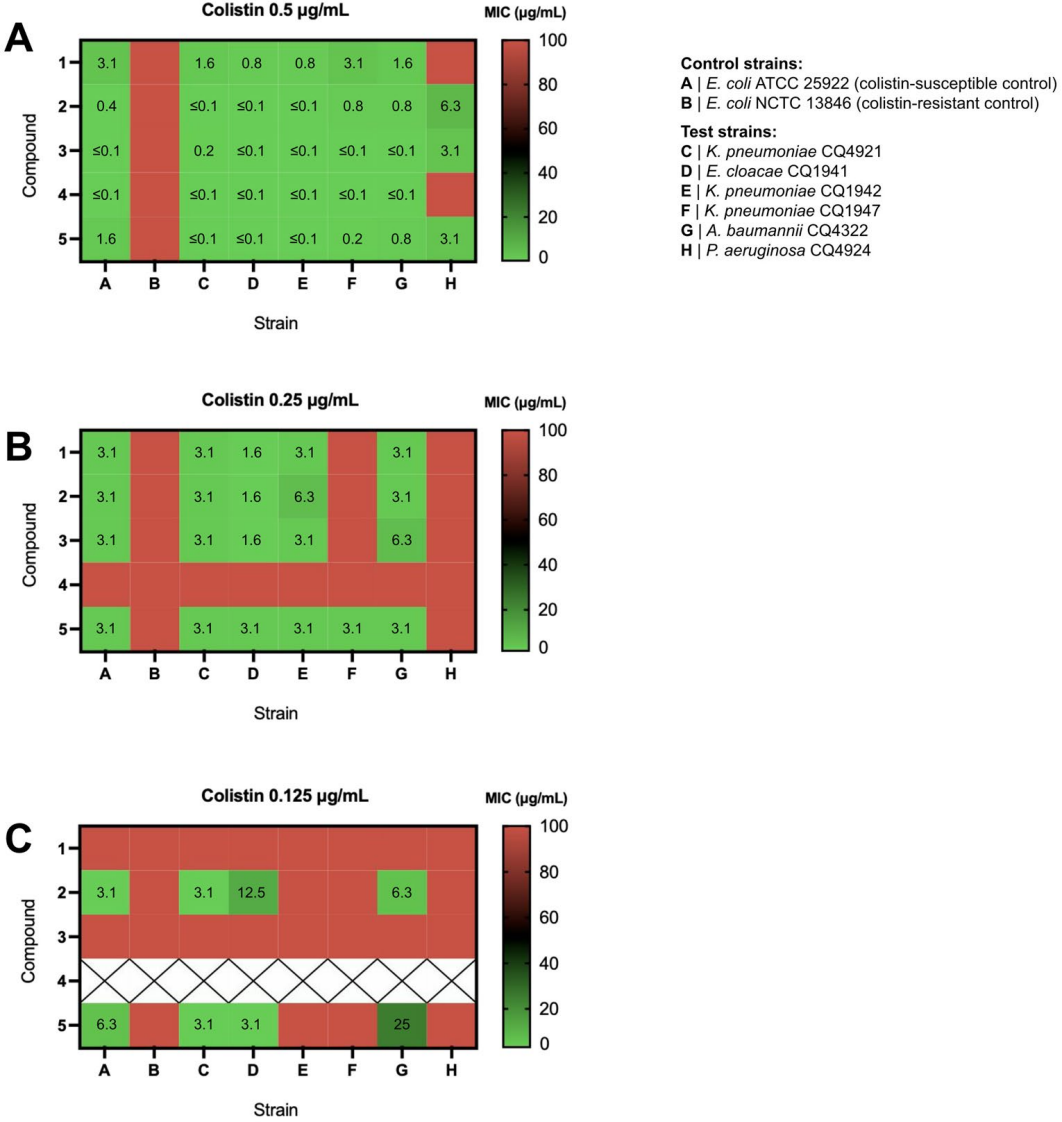
Heatmaps of MIC values for compounds **1–5** in the presence of (**A**) 0.5 μg/mL, (**B**) 0.25 μg/mL and (**C**) 0.125 μg/mL of colistin against critical priority carbapenem-resistant Gram-negative clinical isolates. Results are presented as the median of three independent replicates. X: not tested. Red squares represent MIC values above 100 μg/mL.

The heatmaps depicted in Fig. 5 show the antimicrobial activities of compounds **1–5** when combined with colistin at 0.5 μg/mL, 0.25 μg/mL or 0.125 μg/mL. All in all, with 0.5 μg/mL of colistin (Fig. 5A), both colistin-susceptible control (strain A) and test strains (strains C-H) were susceptible to glycoside-polymyxin combinations, with MIC values ranging from ≤ 0.1 μg/mL to 3.1 μg/mL in the most colistin-susceptible pathogens (strains C-G) and from 3.1 μg/mL to 6.3 μg/mL in *P. aeruginosa* CQ4924 (strain H). For all test strains except for the latter, the use of colistin at the MIC can merely rule out significant antagonistic effects between glycosides and the permeabilizing agent that would potentially hinder its ability to interact with the LPS and interfere with OM integrity. The close-to-zero MIC values observed throughout test strains (in most cases ≤ 0.1 μg/mL) indicate that these glycosides should generally be unable to interfere with the MoA of colistin to a significant extent, with compounds **2** and **5** clearly presenting the potential for additive or synergistic effects (see Supplementary Table S1 online). In fact, by calculating the Fractional Inhibitory Concentration Index (FICI) (Table 2)^28–30^, we were able to assert with more detail that these two glycosides act synergistically with colistin in *E. cloacae* CQ1941 (strain D) and *A. baumannii* CQ4322 (strain G), and additively in the remaining test strains.

**Table 2:** Combined activities of the alkyl deoxyglycosides compounds and colistin against seven carbapenem-resistant and colistin-susceptible reference clinical strains.

Cpd	Clinical isolate	MIC_A_ (mg/L)	MIC_B_ (mg/L)	MIC_A(A + B)_ | MIC_B(A + B)_ (mg/L)^a^	FICI	Interpretation
**1**	*E. coli* ATCC 25922	> 100	0.5	3.125 | 0.25	0.52	Additive
	*K. pneumoniae* CQ4921	> 100	0.5	3.125 | 0.25	0.52	Additive
	*E. cloacae* CQ1941	> 100	0.5	3.125 | 0.25	0.52	Additive
	*K. pneumoniae* CQ1942	> 100	0.5	3.125 | 0.25	0.52	Additive
	*K. pneumoniae* CQ1947	> 100	0.5	3.125 | 0.5	1.016	Indifferent
	*A. baumannii* CQ4322	> 100	0.5	3.125 | 0.25	0.52	Additive
	*P. aeruginosa* CQ4924	> 100	1	-	-	-
**2**	*E. coli* ATCC 25922	> 100	0.5	3.125 | 0.06	0.14	**Synergy**
	*K. pneumoniae* CQ4921	> 100	0.5	3.125 | 0.125	0.27	**Synergy**
	*E. cloacae* CQ1941	> 100	0.5	12.5 | 0.125	0.31	**Synergy**
	*K. pneumoniae* CQ1942	> 100	0.5	6.25 | 0.25	0.53	Additive
	*K. pneumoniae* CQ1947	> 100	0.5	0.8 | 0.5	1.004	Indifferent
	*A. baumannii* CQ4322	> 100	0.5	6.25 | 0.125	0.28	**Synergy**
	*P. aeruginosa* CQ4924	> 100	1	6.25 | 0.5	0.53	Additive
**3**	*E. coli* ATCC 25922	> 100	0.5	3.125 | 0.25	0.52	Additive
	*K. pneumoniae* CQ4921	> 100	0.5	3.125 | 0.25	0.52	Additive
	*E. cloacae* CQ1941	> 100	0.5	1.6 | 0.25	0.51	Additive
	*K. pneumoniae* CQ1942	> 100	0.5	3.125 | 0.25	0.52	Additive
	*K. pneumoniae* CQ1947	> 100	0.5	0.4 | 0.5	1.002	Indifferent
	*A. baumannii* CQ4322	> 100	0.5	6.25 | 0.25	0.53	Additive
	*P. aeruginosa* CQ4924	> 100	1	3.125 | 0.5	0.52	Additive
**4**	*E. coli* ATCC 25922	> 100	0.5	0.4 | 0.5	1.002	Indifferent
	*K. pneumoniae* CQ4921	> 100	0.5	0.4 | 0.5	1.002	Indifferent
	*E. cloacae* CQ1941	> 100	0.5	0.4 | 0.5	1.002	Indifferent
	*K. pneumoniae* CQ1942	> 100	0.5	0.4 | 0.5	1.002	Indifferent
	*K. pneumoniae* CQ1947	> 100	0.5	0.4 | 0.5	1.002	Indifferent
	*A. baumannii* CQ4322	> 100	0.5	0.4 | 0.5	1.002	Indifferent
	*P. aeruginosa* CQ4924	> 100	1	-	-	-
**5**	*E. coli* ATCC 25922	> 100	0.5	12.5 | 0.06	0.18	**Synergy**
	*K. pneumoniae* CQ4921	> 100	0.5	3.125 | 0.125	0.27	**Synergy**
	*E. cloacae* CQ 1941	> 100	0.5	3.125 | 0.125	0.27	**Synergy**
	*K. pneumoniae* CQ1942	> 100	0.5	3.125 | 0.25	0.52	Additive
	*K. pneumoniae* CQ1947	> 100	0.5	3.125 | 0.25	0.52	Additive
	*A. baumannii* CQ4322	> 100	0.5	25 | 0.125	0.38	**Synergy**
	*P. aeruginosa* CQ4924	> 100	1	3.125 | 0.5	0.52	Additive

Cpd, compound; MIC_A_, MIC of each compound individually; MIC_B_, MIC of colistin individually; MIC_A(A+B)_ and MIC_B(A+B)_ are the MIC of each compound and colistin in combination, respectively. FICI, Fractional Inhibitory Concentration Index. ^a^Combination MIC values of compound/colistin with the lowest FICI calculated. ^b^Values ≤ 0.5 were considered synergistic, from 0.5 to 1.0 were additive, 1.0 to 4.0 were indifferent, and ≥ 4.0 were considered antagonistic^28^.

Despite the absence of antagonistic effects (Table 2), there was a lack of antimicrobial activity of **1** towards *P. aeruginosa* CQ4924 (strain H) even in the presence of colistin at 0.5 μg/mL (Fig. 5A). Given that 0.5 μg/mL corresponds to half of the MIC value of colistin against CRPA CQ4924 (and therefore to a concentration that should be unable to cause inhibition of bacterial growth per se in this strain), our results suggest, on the other hand, that compounds **2**, **3** and **5** were able to effectively penetrate the OM and induce IM lysis — particularly **3** and **5**, which exhibited the lowest MIC (3.1 μg/mL). It is important to highlight that, according to the previously established MoA for these types of compounds^6^, the high IM PE levels displayed by Gram-negative bacteria (60–82%^7^), which can furthermore be upregulated in the presence of low colistin concentrations^31^, should be the core factor enabling the effective targeting of the IM, and could furthermore justify the five-fold lower MIC values vs. those reported in Gram-positive pathogens (e.g. 16 μg/mL for compound **3** in *B. cereus* ATCC 14579^6^, exhibiting 43% of PE in the membrane^7^). Moreover, these glycosides were not active per se (i.e. in the absence of polymyxins) in any of the eight tested bacterial strains (MIC > 100 μg/mL, see Supplementary Tables S3and S4 online), thus validating our concept that antimicrobial activity in Gram-negative bacteria is observed due to polymyxin-induced OM permeabilization. As expected, the colistin-resistant *E. coli* NCTC 13846 (strain B; colistin-resistant control) was not susceptible to any of the tested antibiotic combinations.

It is interesting to note the activity observed for β-*O*-glycoside **2** in light of the lack of antimicrobial effects previously reported for this compound in Gram-positive bacteria^6^. Rather than an intrinsic inability of the β-anomer to interact with PE and act as a bactericide, this result strongly points towards other factors contributing to its divergent behaviour when encountering variations in the bacterial cell envelope from Gram-positive to Gram-negative pathogens. We postulate that one possible explanation for this result could be related to target inaccessibility due to enzymatic liability. Indeed, being a β-*O*-glycoside, **2** should display a naturally higher susceptibility to hydrolysis by enzymes with β-glycosidase activity when compared with its α analogue (**1**). If, on the one hand, both Gram-positive and Gram-negative bacteria redundantly express cell wall-associated enzymes required for the cleavage of β-1,4 glycosidic bonds between MurNAc and GlcNAc peptidoglycan units^32^, on the other hand, the regulation of such enzymes may widely vary among types of bacteria and environmental factors^33,34^. Consequently, in case **2** is a substrate of one or more of these peptidoglycan-recycling enzymes, the compound could indeed reach the IM to a lower extent in bacteria with higher rates of cell wall remodeling and turnover.

As for glycoside **4**, it was found to be inactive against *P. aeruginosa* CQ4924 when combined with colistin at 0.5 μg/mL (Fig. 5A), pointing towards the inability of this 2-deoxy glycoside to inhibit bacterial cell growth even in conditions that favour OM permeabilization. This inability was indeed confirmed when the colistin concentration was lowered to 0.25 μg/mL (Fig. 5B), rendering **4** fully inactive even against the antibiotic-susceptible *E. coli* ATCC 25922 (control strain A).

Taken together, the results for compounds **1–3** and **5** (Fig. 5B), demonstrate that colistin 0.25 μg/mL is sufficient for OM permeabilization in all test strains except for *P. aeruginosa* CQ4924 (strain H). Under these conditions, however, compounds **1–3** failed to be active against *K. pneumoniae* CQ1947 (strain F), an IMP-1-producing MDR clinical isolate. *K. pneumoniae* is the most common pathogen causing bloodstream infections, pneumonia, urinary tract infections (UTIs) and peritonitis, being typically associated with infections occurring in hospitalized or immunocompromised patients^35,36^. In the early 2000s, an outbreak of carbapenem-resistant *K. pneumoniae* in the United States marked the onset of the CRE epidemic^37^. In Portugal, carbapenem-resistant *K. pneumoniae* isolates were reported in 2010^38^, suggesting a relatively rapid importation of CRE strains to the European continent. Indeed, these remain a threat to public health to this day: with limited treatment options, patients infected with carbapenem-resistant *K. pneumoniae* are at a significantly higher risk of in-hospital death and poorer health outcomes than those infected with carbapenem-sensitive *K. pneumoniae*^39^, which urgently calls for the identification and development of new antibiotic approaches against *K. pneumoniae* strains expressing carbapenemases. Despite the results for compounds **1–3**, we were pleased to find that, with a subtherapeutic colistin concentration of 0.25 μg/mL, *C*-glycoside **5** exhibited antimicrobial activity against all three carbapenem-resistant *K. pneumoniae* isolates tested, including the IMP-1-producing strain CQ1947 (strain F). This result already denotes a positive impact of the *O → C* bioisosteric replacement from **1** to **5** on the antimicrobial activity of alkyl 4,6-dideoxy-α-D-*xylo* glycosides, and highlights the potential of compound **5** as a lead for further development against CRE.

Using a colistin concentration fourfold lower than the MIC, both *K. pneumoniae* CQ1942 (strain E) and *K. pneumoniae* CQ1947 (strain F) became non-susceptible to any of the glycosides (Fig. 5C). Yet, 0.125 μg/mL was sufficient to allow the entry of compounds **2** and **5** through the OM of *K. pneumoniae* CQ4921 (strain C), *E. cloacae* CQ1941 (strain D) and *A. baumannii* CQ4322 (strain G), which promoted visible antibacterial effects (MIC 3.1–25 μg/mL). Notably, the OXA-48-producing *K. pneumoniae* CQ4921 is not only susceptible to imipenem upon increased exposure but is also susceptible to ciprofloxacin and aminoglycoside antibiotics, contrarily to the above-mentioned imipenem-resistant CQ1942 (KPC-3-producing) and CQ1947 (IMP-1-producing) strains (see Supplementary Table S1 online). Hence, even though all three strains are colistin-susceptible, our results reflect a higher sensitivity of the more susceptible *K. pneumoniae* CQ4921 to colistin-LPS interactions than the remaining two less susceptible *K. pneumoniae* strains. Moreover, to our surprise, the β configuration (**2**) of alkyl 4,6-dideoxy-D-*xylo O*-glycosides seems to be more beneficial to activity at low colistin concentrations than the α configuration (**1**).

As part of the CRE epidemic^37,38^, the reported rise in infections caused by carbapenem-resistant *K. pneumoniae* in the last decade^40^ is accompanied by an increase in the rate of nosocomial infections caused by carbapenem-resistant *E. cloacae* as well^41^. The expression of the New Delhi metallo-β-lactamase 1 (NDM-1) has been described as one of the most common carbapenem resistance-associated mechanisms in this type of bacteria^41–44^, which is captured by *E. cloacae* CQ1941 (strain D), used in the present study. This strain is not only resistant to penicillins, cephalosporins, and aminoglycosides, but also to the synthetic monobactam aztreonam (see Supplementary Table S1 online), which is still seen as a valid bet for pharmaceutical investment. Indeed, aztreonam is currently under Phase 3 trials in combination with avibactam for the treatment of serious infections caused by carbapenemase-producing MDR Gram-negative pathogens for which available therapeutic options are limited^45^. With a MIC of 3.1 μg/mL, *C*-glycoside **5** has the potential to be further explored against this aztreonam-resistant CRE strain when combined with colistin at 0.125 μg/mL, while *O*-glycoside **2** was significantly less active in the same conditions (MIC of 12 μg/mL) (Fig. 5C).

In contrast, **2** was significantly more active against the GES-12-producing *A. baumannii* CQ4322 (strain G) than **5**, in spite of the higher MIC value observed (6.3 μg/mL). In addition to CRPA and CRE, CRAB was also included in our MDR Gram-negative pathogen panel for being a top-priority pathogen in urgent need of novel antibiotic development, with frequent healthcare-associated outbreaks leading to high mortality rates in critically ill patients^4,46^. Hence, even though lead **5** seems to be the most promising glycoside of the series overall, lead **2** may offer a relevant alternative to cover a wider spectrum of pathogens and different carbapenem resistance mechanisms, and thus should also be further explored.

It is worth mentioning that 0.06 μg/mL of colistin led to OM permeabilization in the antibiotic-susceptible control strain *E. coli* ATCC 25922, but not in either of the carbapenem-resistant test strains (see Supplementary Table S3 online). A colistin concentration of 0.125 μg/mL can, therefore, be established as the lowest subtherapeutic colistin dilution with observable OM-permeabilizing effects in MDR Gram-negative pathogens with a MIC of colistin of 0.5 μg/mL (strains C, D and G), while 0.5 μg/mL is the lowest subtherapeutic colistin dilution capable of such effects in CRPA CQ4929 (strain H), which has a MIC of colistin of 1 μg/mL (Table 1).

To corroborate the bactericide MoA that was previously proposed for this class of glycosides, the minimum bactericidal concentration (MBC) was also determined for the best compounds in combination with colistin at 0.125 μg/mL. Since the MBC of compounds **2** and **5** was either equal to or just one dilution higher than the MIC (Table 3), we conclude that these compounds are, indeed, bactericides in Gram-negative bacteria when in the presence of an OM-permeabilizing agent. This is in alignment with previous evidence supporting a bactericidal MoA of alkyl deoxyglycosides in Gram-positive bacteria^6^.

**Table 3:** MIC and MBC (μg/mL) values for the best compounds in combination with colistin against critical priority carbapenem-resistant Gram-negative clinical isolates. MIC values are presented in bolditalic while MBC values are presented in italic. Results are presented as the median of three independent replicates.

*Compound*	*Colistin conc.* (μg/mL)	*A*	*B*	*C*	*D*	*G*
		*E. coli* ATCC 25922	*E. coli* NCTC 13846	*K. pneumoniae* CQ4921	*E. cloacae* CQ1941	*A. baumannii* CQ4322
**2**	0.125	***3.1***|*3.1*	> ***100***|> *100*	***3.1***|*6.3*	***12.5***|*25*	***6.3***|*12.5*
**5**	0.125	***6.3***|*6.3*	> ***100***|> *100*	***3.1***|*6.3*	***3.1****|****6.3***	***25****|****25***

### PMB-glycoside combinations

**Figure 6:**
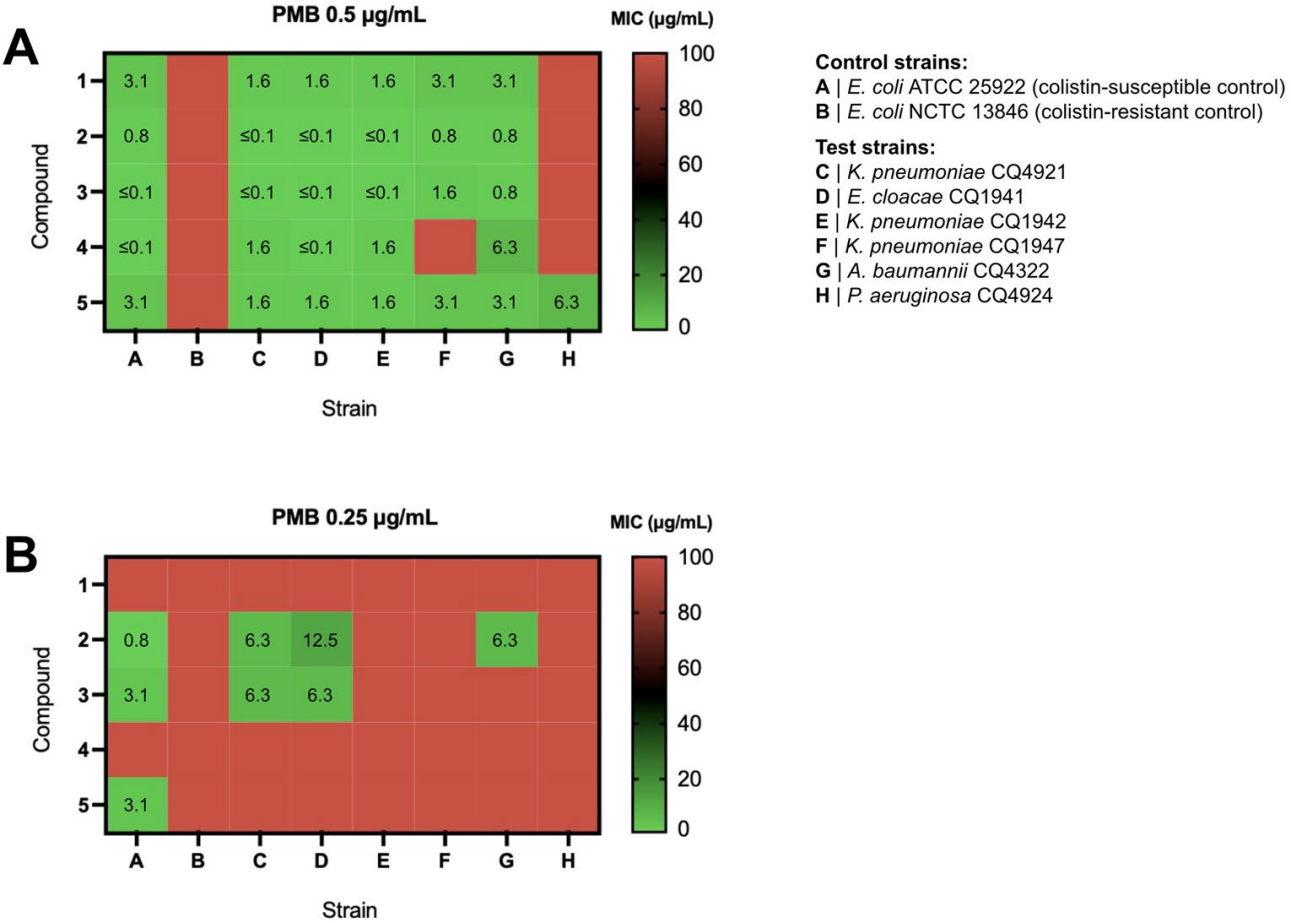
Heatmaps of MIC values for compounds **1–5** in the presence of (**A**) 0.5 μg/mL and (**B**) 0.25 μg/mL of polymyxin B (PMB) against critical priority carbapenem-resistant Gram-negative clinical isolates. MIC values are presented as the median of three independent replicates. Red squares represent MIC values above 100 μg/mL.

As a potentially useful alternative to colistin, PMB was also tested in combination with all five glycosides against the same panel of control and test CRE, CRAB and CRPA strains. The results, shown in Fig. 6 (and in Supplementary Table S4 online), reveal MIC values that were overall higher with PMB as adjuvant agent compared with colistin. For instance, at 0.5 μg/mL of PMB, only *C*-glycoside **5** was active against CRPA CQ4924 (MIC of 6.3 μg/mL) (Fig. 6A). Furthermore, at 0.25 μg/mL, only compounds **2** and **3** were active against a few carbapenem-resistant strains (C, D and G), but with higher MIC values compared with the same concentration of colistin (Fig. 6B). In fact, PMB and colistin are two cationic polypeptide antibiotics differing only in the amino acid residue at position 6 of the peptide ring (D-Phe vs. D-Leu, respectively)^47^. Even though both polymyxins should enhance OM permeability mainly via their amine moieties—which are protonated at physiological pH and involved in a cation-displacement MoA^26,48^—these antibiotics also rely upon their hydrophobic domains to interact with LPS and get inserted into the membrane^47^. In this perspective, our results indicate that the D-leucine residue present in colistin presents benefits in terms of OM permeabilization when used at subtherapeutic concentrations compared to the D-phenylalanine residue of PMB, probably by favouring hydrophobic interactions with LPS. Hence, colistin should be pursued as the most adequate adjuvant agent to be used with these types of glycosides. Lower concentrations of PMB were, therefore, not explored.

### Cytotoxic effects

**Figure 7:**
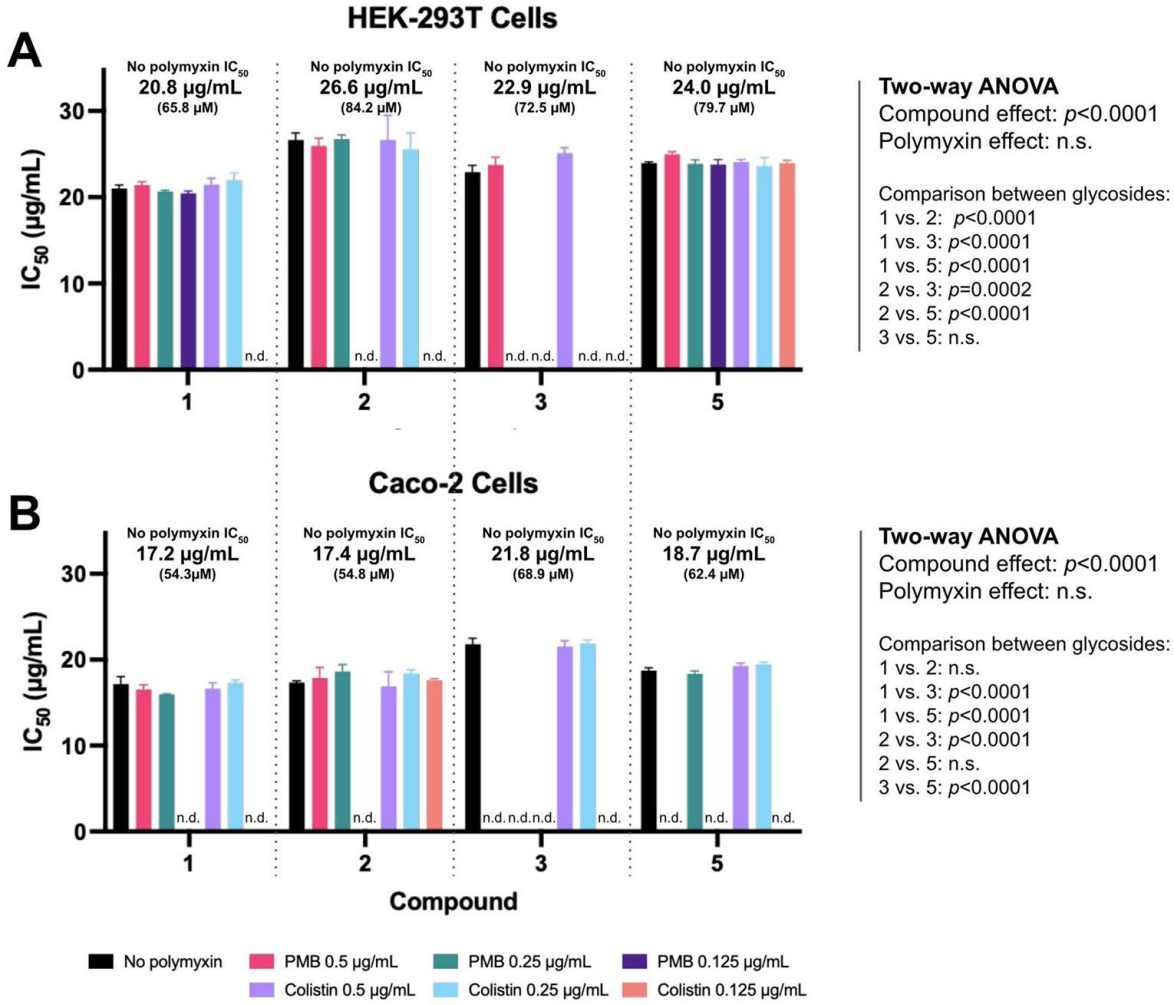
Cytotoxicity of compound combinations with polymyxins in (**A**) HEK-293T cells and (**B**) Caco-2 cells. Results are presented as the mean of three independent replicates ± SD. Statistical differences between conditions were assessed via a two-way ANOVA followed by a Tukey’s multiple comparisons test. n.d. not determined. n.s. not significant.

Cytotoxicity of each compound alone or in combination with different polymyxin concentrations was evaluated through the resazurin cell viability assay^49^ in human embryonic HEK-293T cells, as well as in human colon adenocarcinoma Caco-2 cells, after a 72-h long incubation period. Results (IC_50_ values expressed in μg/mL) for glycoside and glycoside-polymyxin combinations (except for the inactive glycoside **4**) are visually presented in Fig. 7 (IC_50_ values expressed in μM are presented in Supplementary Tables S5 and S6 online).

When tested alone in HEK-293T cells, colistin and PMB displayed an IC_50_ value > 115 μg/mL (not graphically shown), which is in agreement with previously reported values in HEK-293 cells (> 1000 and > 500 μg/mL, respectively)^50^ and other human cell lines^51,52^. An identical result was obtained for both polymyxins in Caco-2 cells (IC_50_ > 115 μg/mL). With regards to glycosides, compounds **1–3** presented IC_50_ values above 17 μg/mL in Caco-2 cells (Fig. 7B), which is also in agreement with previous results^6^, but slightly higher in HEK-293T cells (> 20 μg/mL, Fig. 7A). All in all, our results indicate that Caco-2 cells were more sensitive to the cytotoxic effects caused by the glycosides compared with HEK-293 T cells. Of note, in both cell lines, the inactive glycoside **4** displayed an IC_50_ value > 100 μM (i.e., > 33 μg/mL; see Supplementary Tables S5nd S6online).

Results for glycoside-polymyxin combinations indicate that there is not a significant increase in cytotoxic effects on human cells with the addition of subtherapeutic concentrations of colistin or PMB, regardless of the cell line (HEK-293T cells, Fig. 7A and Caco-2 cells, Fig. 7B). Indeed, a two-way ANOVA revealed a non-significant polymyxin effect, thus denoting that the use of polymyxins as adjuvant agents is effective for Gram-negative OM permeabilization but is not accompanied by increased cytotoxic effects in human cells. This observation is of particular importance given the known nephrotoxic effects of colistin and PMB, despite being considered safe enough for administration in patients with infections caused by Gram-negative bacteria^53^.

On the other hand, statistically significant differences in toxicity were found between glycosides (*p* < 0.0001). Compound **1** alone, with an IC_50_ of 20.8 μg/mL (65.8 μM) in HEK-293T cells and 17.2 μg/mL (54.3 μM) in Caco-2 cells, was significantly more cytotoxic than all the remaining compounds, including its β-epimer (**2**) in HEK-293 T cells (IC_50_ of 26.6 μg/mL or 84.2 μM; *p* < 0.0001), and its *C*-glycoside analogue (**5**) in both cell lines (IC_50_ of 24.0 μg/mL or 79.7 μM in HEK-293 T cells and 18.7 μg/mL or 64.2 μM in Caco-2 cells; *p* < 0.0001 for both comparisons). These results denote that these structural changes (β *vs.* α configuration and *C*- vs. *O*-glycosylation) were not only favourable in terms of antimicrobial activity but also in terms of cytotoxicity.

With these results, we calculated the lowest in vitro therapeutic index (TI = lowest determined IC_50_/MIC) for the microbiologically active glycoside-colistin concentrations. The TI is commonly used to quantify the balance between the efficacy and safety of a given drug, and reflects the therapeutic window that exists between the drug concentration that results in no significant toxicity and the concentration that produces the desired efficacy^54^. As compounds presented the lowest IC_50_ value in Caco-2 cells, the lowest in vitro TI was calculated based on the cytotoxic effects of each combination toward these cells.

**Figure 8:**
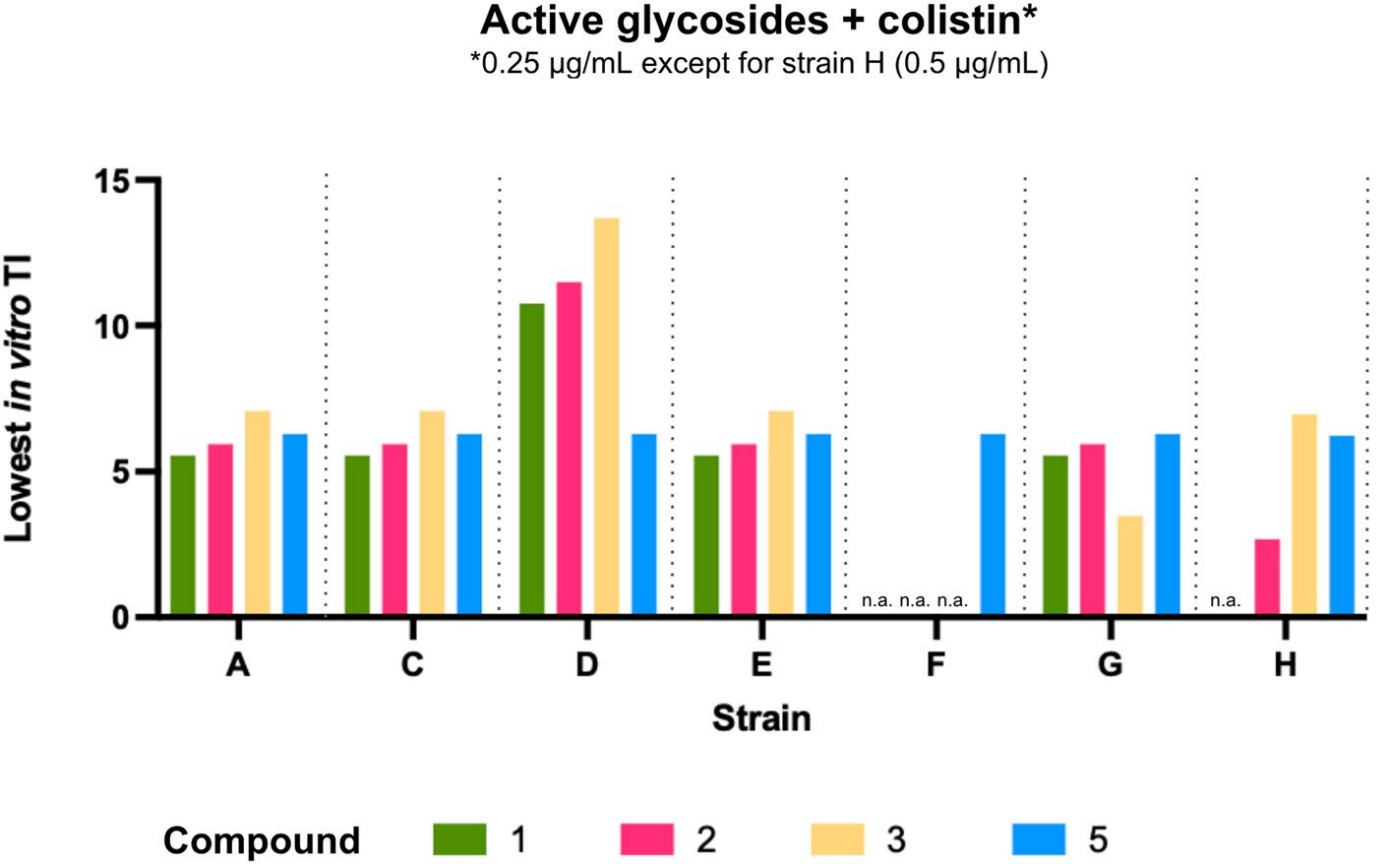
Lowest in vitro therapeutic index (TI) for active compounds in combination with colistin at 0.25 μg/mL (except for strain H, with colistin at 0.5 μg/mL). TIs were calculated based on IC_50_ values in Caco-2 cells for a given glycoside + 0.25 μg/mL or + 0.5 μg/mL, respectively. n.a.: not active.

With colistin 0.5 μg/mL, compounds **3** and **5** presented the best TI against *P. aeruginosa* CQ4924 (strain H; approximately 6.9 and 6.2, respectively, Fig. 8). With colistin 0.25 μg/mL, the best TIs were seen against *E. cloacae* CQ1941 (strain D; maximum observed TI of approximately 13.7 for compound **3**) — the strain for which MIC values were generally the lowest. Importantly, compound **5** presented a consistent TI of approximately 6.3 against all test strains C-G (Fig. 8). Despite seemingly narrow, this value reflects a reasonable (at least sixfold) margin for observing antimicrobial effects without significant toxicity in mammalian cells. Importantly, since the antimicrobial activity was fully retained when using compound **5** plus 0.125 μg/mL of colistin in *K. pneumoniae* CQ4921 and *E. cloacae* CQ1941 (MIC 3.1 μg/mL for both, Fig. 5C) without a significant change in cytotoxicity, this margin was also maintained against these two bacterial targets despite the use of a twofold lower adjuvant concentration. However, the 25 μg/mL MIC against *A. baumannii* CQ4322 (strain G; Fig. 5) exceeded the IC_50_ observed in both HEK-293 T and Caco-2 cells (24.0 μg/mL and 18.7 μg/mL, respectively; Fig. 7), suggesting the potential for general toxicity of compound **5** when used at this concentration against this particular isolate.

## Conclusions

In this study, several dodecyl deoxyglycoside leads were explored in combination with subtherapeutic concentrations of two OM-permeabilizing agents, colistin and PMB, and were successfully repurposed against a panel of critical priority carbapenem-resistant Gram-negative ESKAPE pathogens exhibiting different carbapenem resistance mechanisms. The route toward glycosides **1** and **2** was optimized through a simultaneous dideoxygenation methodology, which enabled the development of a much more expedient synthesis compared to that originally described^21^. Furthermore, a new *C*-glycoside lead (**5**) was herein reported for the first time, and several alternative procedures were exploited to set a solid foundation for its synthesis under optimized conditions. This novel α-*C*-glycoside (**5**) was active against CRPA when combined with colistin at 0.5 μg/mL, as well as against CRAB and several CRE clinical isolates with colistin 0.25 μg/mL and, in some cases, 0.125 μg/mL. With a generally wider range of susceptible clinical isolates compared to other compounds, the *C*-glycoside **5** was found to be the most promising glycoside of the series together with the *O*-glycoside** 2** with β-anomeric configuration. Both were found to display synergistic effects with colistin against clinical isolates of *E. cloacae* and *A. baumannii*. Importantly, the bactericidal MoA of both compounds was confirmed after MBC determination. Since glycoside-PE interactions have been proposed as the main MoA of this class of bactericidal glycosides in Gram-positive bacteria^6^, this result suggests that IM disruption could be the prime cause for the observed antimicrobial effects in Gram-negative pathogens as well. Furthermore, better antimicrobial activities were observed when using colistin as an adjuvant agent rather than PMB, but neither of these polymyxins led to a significant increase in glycoside cytotoxicity when tested in HEK-293T and Caco-2 cells at the OM-permeabilizing subtherapeutic concentrations of 0.5 μg/mL and below. On the other hand, compounds **2** and **5** were significantly less toxic than the corresponding α-*O*-glycoside (**1**), leading to a calculated in vitro therapeutic index of, at least, 6. Together, our results would suggest that alkyl deoxyglycosides could potentially be used with subtherapeutic concentrations of colistin for the targeting of MDR Gram-negative ESKAPE pathogens labeled as a critical priority by the WHO^4^, setting the α-anomer *C*-glycoside **5** and β-anomer *O*-glycoside** 2** as promising lead compounds for further development. Even though according to Dombach et al*.* another molecule (JD1) has already been shown to interfere with the IM of Gram-negative bacteria in conditions where the OM is compromised^27^, to the best of our knowledge this is the first study showing the potential of IM-targeting carbohydrate-based compounds for the treatment of infections caused by MDR Gram-negative pathogens of clinical importance. Furthermore, our results corroborate the previously highlighted importance of adjuvant agents in finding new solutions against Gram-negative AMR^55,56^, and validate the use of subtherapeutic concentrations of polymyxins for this purpose.

## Experimental section

### General experimental procedures

HPLC-grade solvents and reagents were obtained from commercial suppliers and were used without further purification. Thin-layer chromatography (TLC) was carried out on aluminum sheets (20 × 20 cm) coated with silica gel 60F-254 (0.2 mm thick, Merck) with detection by charring with 10% H_2_SO_4_ in ethanol. Column chromatography (CC) was performed using silica gel 230 − 400 mesh (Merck). Nuclear magnetic resonance (NMR) experiments were recorded on a Bruker Avance 400 spectrometer at 298 K, operating at 100.62 MHz for ^13^C and at 400.13 MHz for ^1^H for solutions in CDCl_3_, CO(CD_3_)_2_, or CD_3_OD (Sigma-Aldrich). Chemical shifts are expressed in δ (ppm) and the proton coupling constants *J* in Hertz (Hz), and spectra were assigned using appropriate COSY, DEPT, HMQC, and HMBC spectra. The high-resolution mass spectra of new compounds were acquired on a HR QqTOF Impact II mass spectrometer (Bruker Daltonics Billerica, MA, USA) interfaced with an ESI source operating in the positive ion mode. Samples were analyzed by flow injection analysis (FIA) using an isocratic gradient 30 A:70 B of 0.1% formic acid in water (A) and in acetonitrile (B), at a flow rate of 10 µL min^−1^ over 15 min. Internal calibration was achieved with a solution of ammonium formate 10 mM introduced to the ion source via a 20 µL loop at the beginning of each analysis, using a six-port valve. The full scan mass spectra were acquired over a mass range of 100–1000 m*/z* at a spectra rate of 1 Hz.

### Synthesis of methyl 2,3-Di-*O*-benzyl-4,6-*O*-benzylidene-α-D-glucopyranoside (6)

To a stirring solution of the commercially available methyl α-D-glucopyranoside (7.0 g, 36.0 mmol) in ACN (35 mL), *p*-toluenesulfonic acid monohydrate (137.0 mg, 0.72 mmol, 0.02 equiv.) was added, followed by benzaldehyde dimethyl acetal (10.8 mL, 72.0 mmol, 2.0 equiv.). The reaction was stirred at 82 ºC for 19 h. Then, the reaction was cooled to room temperature, washed with a saturated solution of sodium bicarbonate (50 mL), and extracted with DCM (3 × 50 mL). Organic phases were combined, dried with MgSO_4_, filtered, and concentrated under reduced pressure. The residue was resuspended in a small amount of DCM at 30 ºC, after which cold petroleum ether was added, affording a precipitate that was filtered and dried under vacuum. 4,6-*O*-benzylidene-α-D-glucopyranoside was hence obtained as a white solid in 91% yield, with physical and spectroscopic data that were in agreement with those previously reported in the literature^57﻿^. The compound (3.0 g, 10.6 mmol) was then dissolved in DMF (160 mL) at 0 ºC, after which NaH (60%, dispersion in paraffin; 1.70 g, 42.2 mmol, 4 equiv.) was added in small portions over 5 min. Then, benzyl bromide (5.04 mL, 42.4 mmol, 4 equiv.) was added to the mixture, and the reaction was stirred at room temperature for 17 h. The reaction was quenched with MeOH, and the solvent was evaporated. The residue was then resuspended in EtOAc, washed with brine (50 mL), and extracted with EtOAc (3 × 50 mL). Organic phases were combined, dried with MgSO_4_, filtered, and concentrated under reduced pressure to give a syrup that was purified by CC (Hex/EtOAc 1:0 to 8:1). Compound **6** was obtained as a colorless oil in 91% yield, with physical and spectroscopic data that were in full agreement with the literature^58^.

### Synthesis of methyl 4,6-*O*-benzylidene-α-D-glucopyranoside (7)

A solution of compound **6** (4.0 g, 8.65 mmol) in 80% (v/v) acetic acid (50 mL) was stirred at 50 ºC for 24 h, after which the solvent was co-evaporated with toluene under reduced pressure. The residue was then resuspended in a small amount of DCM at 30 ºC, cold petroleum was added, and the mixture was left at 0 ºC for 2 h. The so-formed white crystals were filtered and dried under reduced pressure, affording compound **7** in 98% yield. R_f_ (Hex/EtOAc 1:1) = 0.21; ^1^H NMR (400.13 MHz, CO(CD_3_)_2_ , 25 ºC): δ 7.42–7.24 (m, 10H, Ar–*H*), 4.88 (AB system, 2H, *J*_A-B_ = 11.25 Hz, Ar-C*H*_2_), 4.80 (d, 1H, *J*_1-2_ = 3.51 Hz, H-1), 4.71 (AB system, 2H, *J*_A-B_ = 12.49 Hz, Ar-C*H*_2_), 4.43 (br t, 1H, OH-4), 3.82–3.65 (m, 3H, H-3, H-6a and H-6b), 3.58–3.52 (m, 2H, H-4 and H-5), 3.46 (dd, 1H, *J*_2-1_ = 3.53 Hz, *J*_2-3_ = 9.59 Hz, H-2), 3.36 (s, 3H, OC*H*_3_), 2.86 (d, 1H, *J* = 13.32 Hz, OH-6); ^13^C NMR (100.62 MHz, CO(CD_3_)_2_, 25 ºC): δ 146.7, 140.0 (Ar-*C*_q_), 129.1, 128.9, 128.54, 128.5, 128.3, 128.0 (Ar–*C*H), 98.7 (C-1), 82.6 (C-3), 80.9 (C-2), 75.6 (Ar-*C*H_2_-3), 73.1 (C-5), 72.9 (Ar-*C*H_2_-2), 71.7 (C-4), 62.7 (C-6), 55.0 (O-CH_3_). HRMS: Calcd. [C_21_H_27_O_6_] 375.1802, found 375.1810 (error -2.0 ppm); Calcd. [C_21_H_26_NaO_6_] 397.1622, found 397.1634 (error -3.0 ppm).

### Synthesis of methyl 4,6-dideoxy-α-D-xylo-hexopyranoside (8)

Compound **7** (1.0 g, 2.67 mmol) was dissolved in anhydrous DCM (43 mL) and pyridine (1.0 mL, 12.3 mmol, 4.6 equiv.) under nitrogen atmosphere, and the mixture was cooled down to 0 ºC. Then, trifluoromethanesulfonic anhydride (2.1 mL, 12.3 mmol, 4.6 equiv.) was added dropwise, and the reaction was left stirring for 2 h and 30 min. As starting material was still detected by TLC, another portion of pyridine (2.2 mL, 2.7 mmol, 1 equiv.) and trifluoromethanesulfonic anhydride (0.46 mL, 2.7 mmol, 1 equiv.) was added as previously described, and the mixture stirred for another 3 h and 30 min. The reaction was quenched with cooled distilled water (100 mL), followed by extraction with DCM (3 × 100 mL). Organic phases were combined and dried with MgSO_4_ filtered, and concentrated under reduced pressure to afford a highly labile brownish oil that was used in the subsequent reaction without further purification. The residue was hence dissolved in toluene (36 mL), and tetra-*n*-butylammonium borohydride (4.13 g, 16.0 mmol, 6 equiv.) was added. The mixture was stirred at 85 ºC for 1 h and 30 min, cooled down to room temperature, poured into cooled distilled water (100 mL) and extracted with DCM (3 × 100 mL). Organic phases were combined and dried with MgSO_4_, filtered, and concentrated under reduced pressure. The resulting residue was purified by CC (Hex/EtOAc 10:1 to 9:1) to afford compound **8** as a colorless oil in 88% yield. R_f_ (CyHex/EtOAc 3:1) = 0.56; ^1^H NMR (400.13 MHz, CDCl_3_, 25 ºC): δ 7.40–7.25 (m, 10H, Ar–*H*), 4.85 (part A of AB system 1, 1H, *J*_A-B_ = 12.13 Hz, Ar-C*H*_2_), 4.74 (AB system 2, 2H, *J*_A-B_ = 11.63 Hz, Ar-C*H*_2_), 4.69 (part B of AB system 2, 1H, *J*_A-B_ = 11.63, Ar-C*H*_2_), 4.63 (d, 1H, *J*_1-2_ = 3.55 Hz, H-1), 3.95–3.83 (m, 2H, H-3 and H-5), 3.46 (dd, 1H, *J*_2-3_ = 9.45 Hz, *J*_2-1_ = 3.61 Hz, H-2), 3.37 (s, 3H, OC*H*_3_), 2.07 (ddd, 1H, *J*_4eq-4ax_ = 12.84 Hz, *J*_4eq-5_ = 4.99 Hz, *J*_4eq-3_ = 2.30 Hz, H-4_ eq_), 1.38 (dt, 1H, *J*_4ax-4 eq_ = 12.60 Hz, *J*_4ax-3_ = *J*_4ax-5_ = 10.68 Hz, H-4_ax_), 1.17 (d, 3H, *J*_6-5_ = 6.35 Hz, H-6); ^13^C NMR (100.62 MHz, CDCl_3_, 25 ºC): δ 139.1, 138.7 (Ar-*C*_q_), 128.5, 128.1, 127.8, 127.7, 127.6 (Ar–*C*H), 99.1 (C-1), 80.6 (C-2), 75.4 (C-3), 73.3, 72.6 (Ar-*C*H_2_), 63.5 (C-5), 55.2 (O*C*H_3_), 39.3 (C-4), 21.0 (C-6). HRMS: Calcd. [C_21_H_26_NaO_4_] 365.1723, found 365.1724 (error -0.2 ppm).

### Synthesis of dodecyl 2,3-di-*O*-benzyl-4,6-dideoxy-α/β-D-xylo-hexopyranoside (9)

A solution of **8** (0.89 g, 2.60 mmol) in anhydrous DCM (5 mL) containing Amberlyst 15 beads (0.195 g) and dodecan-1-ol (2.90 mL, 12.95 mmol, 5 equiv.) was stirred at 40 ºC over 40 h. The reaction mixture was then cooled down to room temperature, diluted in DCM (30 mL), filtered, and concentrated under reduced pressure. The resulting residue was purified by CC (Hex/DCM 1:1), affording the inseparable anomeric mixture (1.35:1 α/β) **9** as a colorless oil in 93% yield. R_f_ (DCM) = 0.6; ^1^H NMR (400.13 MHz, CDCl_3_, 25 ºC): δ 7.40–7.26 (m, 23.5H, Ar–*H*), 4.95 (Part A of AB system, 1H, *J*_A-B_ = 11.05 Hz, Ar-C*H*_2_β), 4.84–4.66 (m, 9.75H, Ar-C*H*_2_, H-1α), 4.31 (d, 1H, *J*_1-2_ = 7.79 Hz, H-1β), 3.96–3.87 (m, 3.7H, H-1ʹaα, H-3α and H-5β), 3.65–3.39 (m, 7.05H, H-1ʹbα, H-1ʹβ, H-2α, H-3β and H-5α), 3.30 (t, 1H, *J*_2-1_ = *J*_2-3_ = 7.80 Hz, H-2β), 2.11–2.00 (m, 2.35H, H-4_ eq_), 1.71–1.58 (m, 5.7H, H-2ʹ and H-4_ax_β), 1.48–1.26 (m, 46.3H, H-4_ax_α, H-6β and H-3ʹ to H-11ʹ), 1.16 (d, 4.05H, *J*_6-5_ = 6.29 Hz, H-6α), 0.89 (t, *J*_12ʹ-11ʹ_ = 7.07 Hz, H-12ʹ); ^13^C NMR (100.62 MHz, CDCl_3_, 25 ºC): δ 139.2, 139.0, 138.9, 138.9 (Ar-*C*_q_), 128.5, 128.4, 128.4, 128.2, 128.0, 127.8, 127.7, 127.6, 127.5 (Ar–*C*H), 103.9 (C-1β), 97.7 (C-1α), 83.0 (C-2β), 80.9 (C-2α), 78.4 (C-3β), 75.5 (C-3α), 75.0, 73.0, 72.6, 72.4 (Ar-*C*H_2_), 70.2 (C-1ʹα or β), 68.0 (C-1ʹα or β), 67.7 (C-5β), 63.6 (C-5α), 39.4 (C-4α), 38.9 (C-4β), 32.1, 30.0, 29.8, 29.8, 29.7, 29.6, 29.6, 29.5, 26.3, 22.8 (C2’ to C11’), 21.1 (C-6β), 21.1 (C-6α), 14.3 (C-12ʹ). HRMS: Calcd. [C_32_H_49_O_4_] 497.3625, found 497.3635 (error -1.9 ppm); Calcd. [C_32_H_48_NaO_4_] 519.3445, found 519.3460 (error -2.8 ppm).

### Synthesis of dodecyl 4,6-dideoxy-α/β-D-xylo-hexopyranoside (1,2)

The anomeric mixture **9** (0.838 g, 1.69 mmol) was dissolved in methanol (12 mL). After the addition of Pd/C 10% (29 mg) under N_2_ atmosphere, triethylsilane (6.45 mL, 40.39 mmol, 23.9 equiv.) was added dropwise and the reaction was stirred at room temperature. After 24 h, the catalyst was filtered off through a pad of Celite, and the solvent was evaporated under reduced pressure, affording an inseparable mixture of anomers as observed by TLC. To allow anomer separation, the residue was dissolved in pyridine (5 mL) and acetic anhydride was added (0.46 mL, 6.76 mmol, 4 equiv.), followed by a spatula tip of DMAP. After stirring for 4 h at room temperature, the reaction reached completion and both anomers were finally visible by TLC. The solvent was removed by co-evaporation with toluene and the residue was purified by CC (Hex/EtOAc 10:1), affording the α anomer as a colorless oil (256 mg, 38% yield), and the β anomer as a white solid (172.7 mg, 26% yield), as previously reported^21^. Each anomer was then separately dissolved in methanol (100 mg/mL), followed by the addition of a 1 M solution of NaOMe in methanol (0.1 mL per 0.1 mg of substrate). For anomer α, the reaction was stirred at room temperature for 2 h, whereas for anomer β the reaction was stirred at room temperature for 1 h. Both mixtures were neutralized with Amberlite IR-120, filtered and the solvent was evaporated. Both final compounds were found to be pure by TLC; however, to ensure maximum purity, both were purified by CC (EA), affording compound **1** as a white solid in 97% yield and compound **2** as a white solid in quantitative yield, with physical and spectroscopic data that were in full agreement with the literature^21^. HRMS for compound **1**: Calcd. [C_18_H_36_NaO_4_] 339.2506, found 339.2512 (error -1.8 ppm); HRMS for compound **2**: Calcd. [C_18_H_36_NaO_4_] 339.2506, found 339.2510 (error -1.1 ppm).

### Synthesis of dodecyl 2,6-dideoxy-α-L-arabino-hexopyranoside (3)

Synthesized according to the previously described methodology^6^. Physical and spectroscopic data are in full agreement with the literature^6^. HRMS: Calcd. [C_18_H_36_NaO_4_] 339.2506, found 339.2513 (error -2.0 ppm).

### Synthesis of dodecyl 2-deoxy-α-D-arabino-hexopyranoside (4)

Synthesized according to the previously described methodology^59^. Physical and spectroscopic data are in full agreement with the literature^59^. HRMS: Calcd. [C_18_H_37_O_5_] 333.2636, found 333.2634 (error 0.6 ppm); Calcd. [C_18_H_36_NaO_5_] 355.2455, found 355.2457 (error -0.5 ppm).

### Synthesis of 1,3-di-*O*-acetyl-2-*O*-benzyl-4,6-dideoxy-α/β-D-xylo-hexopyranoside (10)

To a stirring solution of compound **8** (0.559 g, 1.63 mmol) in acetic acid (5 mL) and acetic anhydride (5 mL) at 0 ºC, H_2_SO_4_ 97% (90 μL) was added dropwise. The reaction was stirred at 0 ºC for the first 4 h, and at room temperature for another 16 h. The mixture was then washed with a saturated solution of sodium bicarbonate (20 mL), extracted with DCM (2 × 20 mL), then washed with distilled water (20 mL), and again extracted with DCM (3 × 20 mL). Organic phases were combined and dried with MgSO_4_, filtered, and concentrated under reduced pressure. The resulting residue was purified by CC (Hex/EtOAc 10:1 to 6:1) to afford the anomeric mixture (1:0.3 α/β) **10** as a white solid in 35% yield. R_f_ (Hex/EtOAc 2:1) = 0.57; ^1^H NMR (400.13 MHz, CDCl_3_, 25 ºC): 7.36–7.28 (m, 6.5H, Ar–*H*), 6.33 (d, 1H, *J*_1-2_ = 3.39 Hz, H-1α), 5.60 (d, 0.3H, *J*_1-2_ = 8.13 Hz, H-1β), 5.19 (dt, 1H, *J*_3-2_ ≈ *J*_3-4ax_ = 10.34 Hz, *J*_3-4 eq_ = 4.66 Hz, H-3α), 4.97 (dt, 0.3H, m, H-3β), 4.73–4.54 (m, 2.6H, Ar-C*H*_2_), 4.11–4.04 (m, 1H, H-5α), 3.81–3.73 (m, 0.3H, H-5β), 3.56 (dd, 1H, *J*_2-1_ = 3.37 Hz, *J*_2-3_ = 9.90 Hz, H-2α), 3.44 (t, 0.3H, *J*_2-1_ = *J*_2-3_ = 8.56 Hz, H-2β), 2.19–2.10 (m, 4.3H, C*H*_3_ OAcα and H-4_ eq_), 2.03 (s, 0.9H, C*H*_3_ OAcβ), 2.03 (s, 3H, C*H*_3_ OAcα), 1.98 (s, 0.9H, C*H*_3_ OAcβ), 1.43–1.33 (m, 1.3H, H-4_ax_), 1.24 (d, 0.9H, *J*_6-5_ = 6.12 Hz, H-6β), 1.17 (d, 3H, *J*_6-5_ = 6.12 Hz, H-6α); ^13^C NMR (100.62 MHz, CDCl_3_, 25 ºC): δ 170.5 (C = O OAcα), 170.4 (C = O OAcβ), 169.9 (C = O OAcα), 169.3 (C = O OAcβ), 138.3 (Ar-*C*_q_β), 137.8 (Ar-*C*_q_α), 128.5, 128.5, 127.9, 127.9, 127.8 (Ar–*C*H), 94.3 (C-1β), 90.6 (C-1α), 79.1 (C-2β), 76.5 (C-2α), 74.8 (Ar-*C*H_2_β), 72.8 (C-3β), 72.7 (Ar-*C*H_2_α), 69.7 (C-3α), 68.8 (C-5β), 66.1 (C-5α), 37.9 (C-4α), 37.8 (C-4β), 21.3 (*C*H_3_ OAc), 21.3 (*C*H_3_ OAcα), 21.2 (*C*H_3_ OAcβ), 20.9 (C-6α), 20.7 (C-6β). HRMS: Calcd. [C_17_H_22_NaO_6_] 345.1309, found 345.1306 (error 0.8 ppm).

### Synthesis of 1,2,3-tri-*O*-acetyl-4,6-dideoxy-α/β-D-xylo-hexopyranoside (11)

To a stirring solution of compound **8** (0.550 g, 1.61 mmol) in ethyl acetate (10 mL) under N_2_ atmosphere, Pd/C 10% (53 mg) was carefully added. The reaction mixture was stirred for 3 h under H_2_ atmosphere, after which the catalyst was filtered off through a pad of Celite, and the solvent evaporated under reduced pressure. The obtained residue was then dissolved in acetic acid (2.26 mL) and acetic anhydride (2.26 mL) at 0 ºC, and H_2_SO_4_ 97% (40 μL) was added dropwise. The reaction was stirred at 0 ºC for the first 4 h, and then at room temperature overnight. The mixture was washed with a saturated solution of sodium bicarbonate (20 mL) and extracted with DCM (3 × 25 mL). Organic phases were combined and dried with MgSO_4_, filtered, and concentrated under reduced pressure. As TLC showed that acetylation was not complete, the residue was resuspended in pyridine (13.5 mL) and acetic anhydride (5.5 mL), and the mixture was stirred for 1 h and 45 min. The solvent was removed by co-evaporation with toluene, affording the anomeric mixture (1:0.3 α/β) **11** as a colorless oil in 90% yield over three steps. R_f_ (Hex/EtOAc 3:1) = 0.57; ^1^H NMR (400.13 MHz, CDCl_3_, 25 ºC): 6.27 (d, 1H, *J*_2-1_ = 3.56 Hz, H-1α), 5.62 (d, 1H, *J*_2-1_ = 7.92 Hz, H-1β), 5.25 (dt, 1H, *J*_3-2_ ≈ *J*_3-4ax_ = 10.44 Hz, *J*_3-4 eq_ = 5.01 Hz, H-3α), 5.04–4.93 (m, 1.6H, H-3β, H-2α and H-2β), 4.11 (dtd, *J*_5-4ax_ = 11.70 Hz, *J*_5-6_ = 5.98 Hz, *J*_5-4 eq_ = 2.20 Hz, 1H, H-5α), 3.79 (dtd, *J*_5-4ax_ = 11.70 Hz, *J*_5-6_ = 5.45 Hz, *J*_5-4 eq_ = 1.94 Hz, 1H, H-5β), 2.23–2.14 (m, 1.3H, H-4_eq_α and H-4_eq_β), 2.13 (s, 3H, OAcα), 2.09 (s, 0.9H, OAcβ), 2.03–2.00 (m, 6.9H, 2 × C*H*_3_ OAcα and 2 × C*H*_3_ OAcβ), 1.56–1.46 (m, 1.3H, H-4_ax_α and H-4_ax_β), 1.27 (d, 0.9H, *J*_6-5_ = 6.15 Hz, H-6β), 1.27 (d, 3H, *J*_6-5_ = 6.36 Hz, H-6α); ^13^C NMR (100.62 MHz, CDCl_3_, 25 ºC): δ 170.6 (C = O OAcα), 170.4 (C = O OAcβ), 170.2 (C = O OAcα), 169.9 (C = O OAcβ), 169.5 (C = O OAcα), 169.4 (C = O OAcβ), 92.4 (C-1β), 90.5 (C-1α), 71.5 (C-2β or C-3β), 71.4 (), 70.5 (C-2α), 69.0 (C-5β), 67.8 (C-3α), 66.3 (C-5α), 37.6 (C-4α), 37.6 (C-4β), 21.2, 21.1 (C*H*_3_ OAcα), 21.1, 21.1, 20.9 (*C*H_3_ OAcβ), 20.8 (*C*H_3_ OAcα and C-6α), 20.8 (C-6β). HRMS: Calcd. [C_12_H_19_O_7_] 275.1125, found 275.1118 (error -0.3 ppm); Calcd. [C_12_H_18_NaO_7_] 297.0945, found 297.0950 (error -1.8 ppm).

### Synthesis of 1-(3-*O*-acetyl-2-*O*-benzyl-4,6-dideoxy-α-D-xylo-hexopyranosyl)prop-2-ene (12)

To a solution of compound **10** (0.173 g, 0.54 mmol) in dry ACN (1 mL), allyltrimethylsilane (0.18 mL, 1.13 mmol, 2.1 equiv.) was added at 0 ºC under N_2_ atmosphere. Then, boron trifluoride etherate (0.28 mL, 2.25 mmol, 4.2 equiv.) was added in a dropwise manner, and the reaction was stirred for 30 min at 0 ºC. The mixture was then washed with a saturated solution of sodium bicarbonate (5 mL), extracted with DCM (2 × 10 mL), then washed with distilled water (10 mL), and again extracted with DCM (2 × 10 mL). Organic phases were combined and dried with MgSO_4_, filtered, and concentrated under reduced pressure, and the residue was purified by CC (Hex/Acetone 1:0 to 10:1) to afford compound **12** as a colorless oil in 70% yield. R_f_ (Hex/EtOAc 5:1) = 0.46; ^1^H NMR (400.13 MHz, CDCl_3_, 25 ºC): 7.36–7.28 (m, 5H, Ar–*H*), 5.85–5.75 (m, 1H, H-2), 5.13–5.06 (m, 3H, H-3 and H-3ʹ), 4.61 (s, 2H, Ar-C*H*_2_), 4.08 (dt, 1H, *J*_1ʹ-1a_ = 10.74 Hz, *J*_1ʹ-2ʹ_ ≈ *J*_1ʹ-1b_ = 5.40 Hz, H-1ʹ), 3.88–3.80 (m, 1H, H-5ʹ), 3.60 (dd, 1H, *J*_2ʹ-3ʹ_ = 9.14 Hz, *J*_2ʹ-1ʹ_ = 5.50 Hz, H-2ʹ), 2.54–2.39 (m, 2H, H-1), 2.13 (ddd, 1H, *J*_4ʹeq-4ʹax_ = 12.81 Hz, *J*_4ʹeq-5ʹ_ = 4.97 Hz, *J*_4ʹeq-3ʹ_ = 2.49 Hz, H-4ʹ_eq_), 2.04 (s, 3H, *C*H_3_ OAc), 1.38 (dt, 1H, *J*_4ʹax-4ʹeq_ = 12.61 Hz, *J*_4ʹax-3ʹ_ = *J*_4ʹax-5ʹ_ = 10.70 Hz, H-4ʹ_ax_), 1.15 (d, 3H, *J*_6ʹ-5ʹ_ = 6.36 Hz, H-6ʹ); ^13^C NMR (100.62 MHz, CDCl_3_, 25 ºC): δ 170.7 (C = O OAc), 138.4 (Ar-*C*_q_β), 135.1 (C-2), 128.5, 127.9, 127.8 (Ar–*C*H), 117.0 (C-3), 77.6 (C-2ʹ), 73.8 (C-1ʹ), 72.8 (Ar-*C*H_2_), 70.5 (C-3ʹ), 63.9 (C-5ʹ), 37.9 (C-4ʹ), 30.7 (C-1), 21.4 (*C*H_3_ OAc), 21.1 (C-6ʹ). The presence of a single benzyl group at position 2 was unequivocally confirmed by HMBC. HRMS: Calcd. [C_12_H_24_NaO_4_] 327.1567, found 327.1572 (error -1.5 ppm).

### Synthesis of 1-(2,3-di-*O*-acetyl-4,6-dideoxy-α-D-xylo-hexopyranosyl)prop-2-ene (13)

To a solution of compound **11** (0.43 g, 1.57 mmol) in ACN (3 mL), allyltrimethylsilane (0.65 mL, 3.29 mmol, 2.1 equiv.) was added at 0 ºC under N_2_ atmosphere. Then, boron trifluoride etherate (0.81 mL, 6.58 mmol, 4.2 equiv.) was added in a dropwise manner, and the reaction was stirred for 3 h at 0 ºC. The mixture was then washed with a saturated solution of sodium bicarbonate (100 mL), extracted with DCM (2 × 100 mL), then washed with distilled water (100 mL), and again extracted with DCM (2 × 100 mL). Organic phases were combined and dried with MgSO_4_, filtered, and concentrated under reduced pressure, and the residue was purified by CC (CyHex/EtOAc 1:0 to 20:1) to afford compound **13** as a colorless oil in 86% yield. R_f_ (CyHex/EtOAc 3:1) = 0.49; ^1^H NMR (400.13 MHz, CDCl_3_, 25 ºC): 5.77 (ddt, 1H, *J*_2-3_trans = 17.12 Hz, *J*_2-3_cis = 10.25 Hz, *J*_2-1_ = 6.86 Hz, H-2), 5.15–5.06 (m, 3H, H-3 and H-3ʹ), 4.99 (dd, 1H, *J*_2ʹ-3ʹ_ = 9.36 Hz, *J*_2ʹ-1ʹ_ = 5.40 Hz, H-2ʹ), 4.18 (dt, 1H, *J*_1ʹ-1a_ = 10.20 Hz, *J*_1ʹ-2ʹ_ ≈ *J*_1ʹ-1b_ = 5.26 Hz, H-1’), 3.87 (dqd, 1H, *J*_5ʹ-4ʹax_ = 9.09 Hz, *J*_5ʹ-4ʹeq_ = 6.19 Hz, *J*_5ʹ-6ʹ_ = 6.25 Hz, H-5ʹ), 2.53–2.44 (m, 1H, H-1a), 2.30–2.23 (m, 1H, H-1b), 2.13 (ddd, 1H, *J*_4ʹeq-4ʹax_ = 12.92 Hz, *J*_4ʹeq-5ʹ_ = 5.08 Hz, *J*_4ʹeq-3ʹ_ = 2.77 Hz, H-4ʹ_eq_), 2.04 (s, 3H, *C*H_3_ OAc), 2.03 (s, 3H, *C*H_3_ OAc), 1.45 (dt, 1H, *J*_4ʹax-4ʹeq_ = 13.01 Hz, *J*_4ʹax-3ʹ_ = *J*_4ʹax-5ʹ_ = 10.24 Hz, H-4ʹ_ax_), 1.18 (d, 3H, *J*_6ʹ-5ʹ_ = 6.29 Hz, H-6ʹ); ^13^C NMR (100.62 MHz, CDCl_3_, 25 ºC): δ 170.5 (C = O OAc), 170.2 (C = O OAc), 134.2 (C-2), 117.3 (C-3), 72.1 (C-1ʹ), 71.5 (C-2ʹ), 68.6 (C-3ʹ), 64.2 (C-5ʹ), 37.3 (C-4ʹ), 31.4 (C-1), 21.2 (*C*H_3_ OAc), 21.0 (*C*H_3_ OAc), 20.9 (C-6ʹ). HRMS: Calcd. [C_13_H_21_O_5_] 257.1384, found 257.1386 (error -1.0 ppm); Calcd. [C_13_H_20_NaO_5_] 279.1203, found 279.1207 (error -1.6 ppm).

### Synthesis of 1-(4,6-dideoxy-α-D-xylo-hexopyranosyl)dodecane (5)

To a solution of **12** (0.157 g, 0.52 mmol) or **13** (0.330 g, 1.30 mmol) in dry DCM (3.1 mL or 7 mL, respectively), the 2nd generation Grubbs-Hoveyda catalyst (10%) was added under N_2_ atmosphere, followed by undec-1-ene (5.5 equiv.). Each mixture was stirred for 24 h at 40 ºC, and the solvent was evaporated under reduced pressure. To remove the catalyst and the excess of undec-1-ene, a flash CC (Hex/Acetone 10:1 and Hex/EtOAc 3:1, respectively) afforded the metathesis products (3-*O*-acetyl-2-*O*-benzyl-4,6-dideoxy-α-D-*xylo*-hexopyranosyl-dodec-2-ene: 0.170 g, 77% yield; 2,3-di-*O*-acetyl-4,6-dideoxy-α-D-*xylo*-hexopyranosyl-dodec-2-ene: 0.480 g, 96% yield) that were used in the following reactions without further characterization. Each compound (0.150 g and 0.430 g, respectively) was then dissolved in EtOAc (5 mL or 6 mL, respectively) under N_2_ atmosphere, after which Pd/C 10% (20 mg or 57 mg, respectively) was added. The mixtures were stirred under H_2_ atmosphere at room temperature (for 20 h and 6 h, respectively). Upon reaction completion, the catalyst was filtered off through a pad of Celite, and the solvent was evaporated under reduced pressure, and each crude was individually submitted to deacetylation according to the procedure described for compound **1**. After 1 h, both mixtures were neutralized with Amberlite IR-120, filtered and the solvent evaporated. Finally, CC (Hex/Acetone 1:0 to 6:1) afforded compound **5** as a white solid in 56% or 85% overall yield over three steps, respectively. R_f_ (Hex/Acetone 2:1) = 0.42; ^1^H NMR (400.13 MHz, CDCl_3_, 25 ºC): 3.95 (dt, 1H, *J*_1ʹ-1a_ = 9.80 Hz, *J*_1ʹ-2ʹ_ ≈ *J*_1ʹ-1b_ = 5.00 Hz, H-1ʹ), 3.82–3.70 (m, 2H, H-3ʹ and H-5ʹ), 3.62 (dd, 1H, *J*_2ʹ-3ʹ_ = 9.00 Hz, *J*_2ʹ-1ʹ_ = 5.91 Hz, H-2ʹ), 2.37 (br d, 2H, OH-2ʹ and OH-3ʹ), 1.98 (ddd, 1H, *J*_4ʹeq-4ʹax_ = 12.92 Hz, *J*_4ʹeq-5ʹ_ = 5.02 Hz, *J*_4ʹeq-3ʹ_ = 2.30 Hz, H-4ʹ_eq_), 1.63–1.51 (m, 2H, H-1), 1.48–1.16 (m, 23H, H-2 to H-11 and H-6ʹ), 0.87 (t, 3H, *J*_6ʹ-5ʹ_ = 6.65 Hz, H-6ʹ); ^13^C NMR (100.62 MHz, CDCl_3_, 25 ºC): δ 76.0 (C-1ʹ), 74.5 (C-2ʹ), 69.0 (C-3ʹ), 64.1 (C-5ʹ), 40.9 (C-4ʹ), 32.1, 29.8, 29.8, 29.8, 29.7, 29.5, 25.9, 24.8, 22.8 (C-2 to C-11), 21.5 (C-6ʹ), 14.3 (C-12). HRMS: Calcd. [C_18_H_37_O_3_] 301.2737, found 301.2742 (error -1.7 ppm); Calcd. [C_18_H_36_NaO_3_] 323.2557, found 323.2563 (error -1.8 ppm).

### Bacterial strains and antibacterial activity assays

The *E. coli* ATCC 25922 and *E. coli* NCTC 13846 reference strains were used as polymyxin-susceptible and polymyxin-resistant bacterial control strains, respectively. Test strains comprised carbapenem-resistant clinical isolates *K. pneumoniae* CQ4921, *E. cloacae* CQ1941, *K. pneumoniae* CQ1942, *K. pneumoniae* CQ1947, *A. baumannii* CQ4322, and *P. aeruginosa* CQ4924, from INSA’s strain collection. Information on antibiotic susceptibility and resistance mechanisms of each of these strains can be found in Supplementary Table S1 online. In vitro susceptibility to glycosides with or without polymyxins (colistin sulfate and polymyxin B sulfate for microbiological assays, European Pharmacopoeia Reference Standards, Sigma-Aldrich) was assessed by MICs. An in-house broth microdilution method was used in a two-fold concentration series according to the modified method accredited at NRL-AR/HAI and recommended by the EUCAST (2022), which follows the ISO 20776–1 (2006) and ISO 20776–2 (2007). Briefly, antibiotic combinations and controls were prepared at the desired dilutions in 96-well plates. The compounds underwent a serial dilution from 0.1 to 100 µg/mL, while colistin or PMB remained fixed at concentrations of 0.06, 0.125, 0.25 or 0.5 µg/mL. Bacterial cells were added to obtain a final concentration of 5 × 10^5^ CFU/mL and a final volume of 200 µL per well. The preparation of the 96-well plates and the distribution of the inoculum were carried out automatically using the Precision Microplate Pipetting System (Biotek). The plates were incubated at 35 °C for 18 h. The lowest concentration of compound and polymyxin that showed no growth was determined as the combination MIC and the lowest concentration causing at least a 99.9% reduction in CFU/mL compared to the starting inoculum was determined as the combination MBC. All experiments were carried out in triplicate.

The fractional inhibitory concentration index (FICI) of each combination (compound/ polymyxin) was calculated according to the following formula: FICI = [MIC_A(A+B)_/MIC_A_] + [MIC_B(A+B)_/MIC_B_], where MIC_A(A+B)_ represents the MIC of compound A within the compound A and compound B combination, while MIC_B(A+B)_ represents the MIC of compound B within the same combination. Values of FICI ≤ 0.5 were considered synergistic, from 0.5 to 1.0 were additive, 1.0 to 4.0 were indifferent, and ≥ 4.0 were considered antagonistic^28^.

### Cytotoxicity assays

HEK-293T and Caco-2 cells were grown in RPMI 1640 medium (VWR) supplemented with 10% FBS (Biowest), 1% PenStrep (Sigma Aldrich), and 1% glutamine (Biowest) in a humidified incubator at 37 °C with a 5% CO_2_ atmosphere. Twice a week, the medium was aspirated, the cells were washed with DPBS (VWR), trypsinized with trypsin–EDTA solution (Biowest), and resuspended in fresh culture medium. The dose-dependent effects of each glycoside or glycoside-polymyxin combination were evaluated by the resazurin cell viability assay^49^. Polymyxins (colistin sulfate and PMB sulfate for microbiological assays, European Pharmacopoeia Reference Standards, Sigma-Aldrich) were firstly dissolved in sterilized Mili-Q water to a stock concentration of 1 mg/mL, while glycosides were dissolved in DMSO to a stock concentration of 30 mM. 1.5 × 10^4^ cells/well in 96.5 µL of medium were seeded onto 96-well plates and incubated at 37 °C for 24 h. For each compound, ten stock dilutions (with concentrations from 750 to 75 µM) were prepared in fresh medium immediately prior to incubation. 16 µL of each dilution were added to each well so that the final glycoside concentrations ranged from 100 to 10 µM in each row in a final assay volume of 120 µL. In addition, the previously prepared polymyxin stock solutions were diluted in fresh medium, affording new stocks of 8, 4, and 2 µg/mL. The same volume (7.5 µL/well) of PMB or colistin was then added to each row to achieve a final polymyxin concentration of 0.5, 0.25, and 0.125 µg/mL, respectively, regardless of the glycoside concentration. In assays without polymyxin, the same volume (7.5 µL/well) of medium was added. In all assays, the maximum DMSO concentration was 0.33%. After a 72-h incubation at 37 ºC, 12 µL of a 0.15 mg/mL resazurin (Sigma-Aldrich) in sterilized Mili-Q water were added to each well, and the plates were left incubating for another 4 h at 37 ºC. Resorufin was measured at 570/600 nm, and the percentage of resazurin reduction, corresponding to the cell viability percentage, was calculated according to Eq. (1)^60^. IC_50_ values were obtained by best fitting the dose-dependent inhibition curves using the Prism 9® software. The results were expressed as the mean ± SD of three independent replicates, with only data from analysis with *R*^*2*^ > 0.90 being considered.

1 $$\% Cell\; viability = \frac{{Abs_{sample} - Abs_{medium\;control } }}{{Abs_{cell\;control } - Abs_{medium \;control } }} \times 100$$

### Statistical analysis

Cell viability results were evaluated through a two-way ANOVA followed by a Tukey’s multiple comparisons test using Prism 9® software. Results were considered statistically significant when the *p*-value was equal to or inferior to 0.05.

### Supplementary Information

Supplementary Information.

## Data Availability

The data that support the findings of this study are available online in the Supplementary Information of this article.
