# Supplementary material for: Alkyl deoxyglycoside-polymyxin combinations against critical priority carbapenem-resistant gram-negative bacteria
Source: Sci Rep. 2024 Jan 26;14:2219. doi: 10.1038/s41598-024-51428-6 (PMC10817917; doi:10.1038/s41598-024-51428-6)
Supplement: Supplementary file 1 — Supplementary Information. [file 41598_2024_51428_MOESM1_ESM.pdf]

Supplementary Information

Alkyl Deoxyglycoside-Polymyxin Combinations against Critical Priority Carbapenem-resistant Gram-negative Bacteria

Ana M. de Matos,[1,*] Patrícia Calado,[1] Mónica Miranda,[1] Rita Almeida,[1] Amélia P. Rauter,[1] M. Conceição Oliveira,[2] Vera Manageiro,[3,4,5] Manuela Caniça[3,4,5,6]

*Corresponding author: amamatos@fc.ul.pt

[1]Centro de Química Estrutural, Institute of Molecular Sciences, Departmento de Química e Bioquímica, Faculdade de Ciências, Universidade de Lisboa, Campo Grande, Lisbon, 1749-016, Portugal. [2]Centro de Química Estrutural, Institute of Molecular Sciences, Instituto Superior Técnico, Av. Rovisco Pais, Lisbon, 1049-001, Portugal. [3]National Reference Laboratory of Antibiotic Resistances and Healthcare-Associated Infections, Department of Infectious Diseases, National Institute of Health Dr. Ricardo Jorge, 1649-016, Lisbon, Portugal. [4]Centre for the Studies of Animal Science, Institute of Agrarian and Agri-Food Sciences and Technologies, University of Porto, Porto, Portugal. [5]AL4AnimalS, Associate Laboratory for Animal and Veterinary Sciences, Lisbon, Portugal. [6]CIISA, Center for Interdisciplinary Research in Animal Health, Faculty of Veterinary Medicine, University of Lisbon, Lisbon, Portugal.

Table of Contents

1. Supplementary Methods 2
   1.1. Synthesis 2
2. Supplementary Tables 8
   2.1. Characterization of clinical isolates 8
   2.2. MIC values (Antimicrobial Activity Assays) 10
   2.3. IC_{50} values (Cytotoxicity Assays) 13
3. Compound Characterization 14
   3.1. NMR Spectra 14
   3.2. HRMS Spectra 28

1. Supplementary Methods

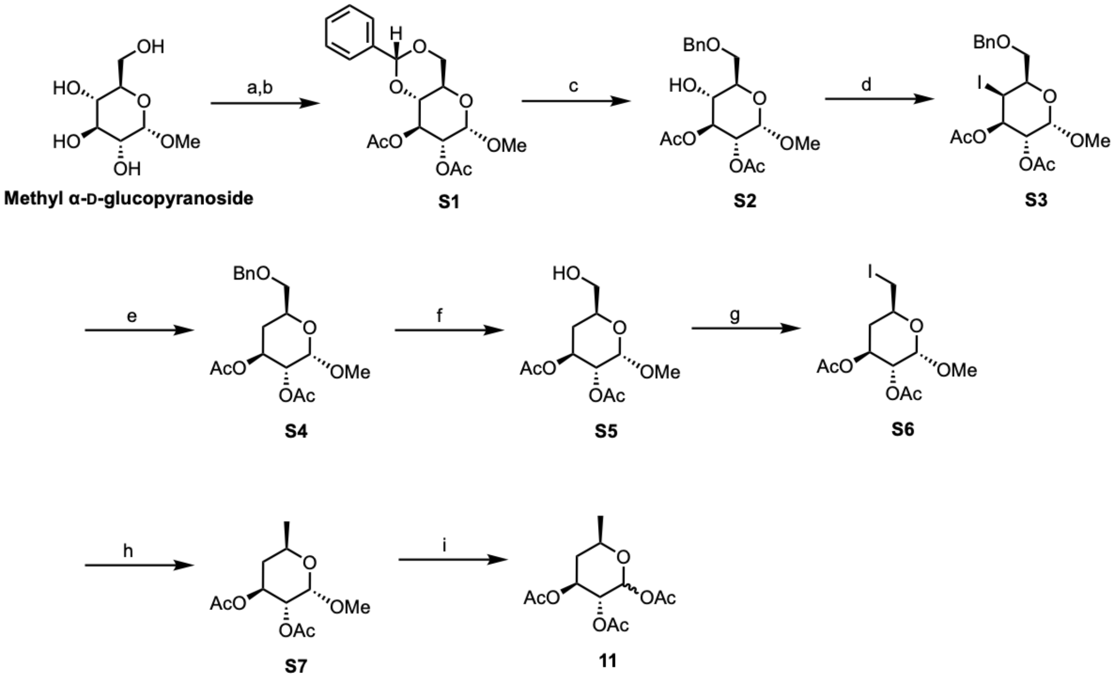

Figure S1. Synthesis of 4,6-dideoxy intermediate 11 through a sequential iodination-reduction approach.[a] Reagents and conditions: **a)** $PhCH(OMe)_2$, *p*-$TsOH{\cdot}H_2O$, ACN, 82 ºC, 19 h, 91%; **b)** Ac_2O, py, rt, 2 h, 80%; **c)** $NaBH_3CH$, I_2, ACN, rt, 1 h, 63%; **d)** I_2, PPh_3, Im, Tol, ACN, 70 ºC, 5 h, 84%; **e)** H_2, Pd/C, EtOAc, DIPEA, 24 h, 94%; **f)** H_2, Pd/C, EtOAc, 24 h, 96%; **g)** I_2, PPh_3, Im, Tol, ACN, 70 ºC, 1 h, 91%; **h)** H_2, Pd/C, EtOAc, DIPEA, 24 h, 66%; **i)** Ac_2O/AcOH (1:1), H_2SO_4, 0 ºC, 1 h, 84%.

[a]Simultaneous reduction of methyl 2,3-di-*O*-acetyl-4,6-dideoxy-4,6-diiodo-α-D-galactopyranoside gave both **S7** and methyl 2,3-di-*O*-acetyl-4,6-cyclo-4,6-dideoxy-α-D-galactopyranoside (data not shown), as confirmed by data in the literature,[1] and hence this route was not further pursued.

[1] Wessel H.-P.; Viaud, M.C.; Gardon, V. Preparation of 4,6-cyclo-hexopyranoses by palladium-mediated intramolecular cyclodehalogenation. *Carbohydr. Res.* **1993**, 245, 233-244.

1.1. Synthesis

1.1.1. Methyl 2,3-di-*O*-acetyl-4,6-*O*-benzylidene-α-D-glucopyranoside (S1). 4,6-*O*-benzylidene-α-D-gluco-pyranoside was synthesized as described in the experimental section of the main text for compound **6**. Then, this intermediate (2.0 g, 7.08 mmol) was dissolved in pyridine (6.5 mL) and acetic anhydride (3 mL), and a spatula tip of DMAP was added. The reaction was left stirring at room temperature for 2 hours, after which the solvent was removed by co-evaporation with toluene. The residue was recrystallized in diethyl ether, affording compound **S1** as a white crystalline solid in 73% overall yield over two steps and spectroscopic data in full agreement with the literature.[2]

1.1.2. Methyl 2,3-di-*O*-acetyl-6-*O*-benzyl-α-D-glucopyranoside (S2). Compound **S1** (2.0 g, 5.96 mmol) was dissolved in ACN (40 mL) in the presence of 4Å and sodium cyanoborohydride (1.80 g, 28.7 mmol, 4.8 equiv.). Iodine (5.32 g, 28.7 mmol, 4.8 equiv.) was then added portion-wise over a period of 15 minutes, and the mixture was stirred at room temperature under N_2 atmosphere for 1 hour. The mixture was diluted in DCM, filtered through a pad of Celite, and the resulting filtrate was washed with a 10% (w/v) solution of sodium carbonate (50 mL), and then with water (50 mL). The organic phase was dried with Na_2SO_4, filtered and the solvent evaporated. The residue was purified by CC (P. Ether/EtOAC 5:1 to 1:1), affording compound **S2** as a colorless oil in 63% yield. R_f (Hex/EtOAc 1:1) = 0.28; **^{1}H NMR** (400.13 MHz, $CDCl_3$, 25 ºC): δ 7.36-7.28 (m, 5H, Ar-*H*), 5.32 (t, 1H, $J_{3\text{-}2}$ = $J_{3\text{-}4}$ = 10.17 Hz, H-3), 4.92 (br s, 1H, H-1), 4.87 (br d, 1H, $J_{2\text{-}3}$ = 10.19 Hz, H-2), 4.60 (AB system, 2H, $J_{A\text{-}B}$ = 11.92 Hz, Ar-CH_2), 3.83-3.72 (m, 4H, H-4, H-5 and H-6), 3.40 (s, 3H, OCH_3), 2.09 (s, 3H, CH_3 OAc), 2.08 (s, 3H, CH_3 OAc); **^{13}C NMR** (100.62 MHz, $CDCl_3$, 25 ºC): δ 171.6 (C=O OAc), 170.4 (C=O OAc), 137.8 (Ar-C_q), 128.5, 127.8, 127.7 (Ar-*C*H), 96.8 (C-1), 73.7 (Ar-*C*H_2), 73.2 (C-3), 70.8 (C-2), 70.3 (C-4 or C-5), 70.2

[2] Matos, A.M.; Nunes, R.; Dias, C.; Rauter, A.P. Cyclic acetals for regioselective protection in carbohydrate synthesis: A comparative experiment. Comprehensive Organic Chemistry Experiments for the Laboratory Classroom. **2017**. Royal Society of Chemistry. pp188-193.

(C-4 or C-5), 69.4 (C-6), 55.3 (O*C*H_3), 21.0 (*C*H_3 OAc), 20.9 (*C*H_3 OAc). **HRMS:** Calcd. [$C_{18}H_{25}O_8$] 369.1544, found 369.1550 (error -1.6 ppm); Calcd. [$C_{18}H_{24}NaO_8$] 391.1363, found 391.1368 (error -1.1 ppm).

1.1.3. Methyl 2,3-di-*O*-acetyl-6-*O*-benzyl-4-deoxy-4-iodo-α-D-galactopyranoside (S3). Compound **S2** (1.05 g, 2.85 mmol) was dissolved in a 2:1 mixture of toluene and ACN (15 mL), together with triphenylphosphine (1.82 g, 6.93 mmol, 2.43 equiv.), imidazole (0.57 g, 8.43 mmol, 2.96 equiv.) and iodine (2.48 g, 9.77 mmol, 3.43 equiv.). The reaction was stirred at 70 ºC for 5 hours, after which the mixture was cooled down to room temperature, neutralized with a saturated solution of sodium thiosulphate (25 mL) and extracted with EtOAc (3 x 25 mL). Organic phases were combined, dried with $MgSO_4$, filtered and the solvent evaporated, and the residue was purified by CC (Hex/EtOAc 1:0 to 7:1) to afford compound **S3** as a yellow oil in 84% yield. R_f (Hex/EtOAc 2:1) = 0.66; **^{1}H NMR** (400.13 MHz, $CDCl_3$, 25 ºC): δ 7.31-7.22 (m, 5H, Ar-*H*), 5.15 (br d, 1H, $J_{2\text{-}3}$ = 10.32 Hz, H-2), 4.91 (br s, 1H, H-1), 4.73 (br s, 1H, H-4), 4.56-4.45 (m, 3H, H-3 and Ar-CH_2), 3.62-3.57 (m, 1H, H-6a), 3.47-3.37 (m, 2H, H5 and H-6b), 3.33 (s, 3H, OCH_3), 2.04 (s, 3H, CH_3 OAc); 2.02 (s, 3H, CH_3 OAc); **^{13}C NMR** (100.62 MHz, $CDCl_3$, 25 ºC): δ 169.7 (C=O OAc), 169.3 (C=O OAc), 137.5 (Ar-C_q), 128.1, 127.5, 127.4 (Ar-*C*H), 96.7 (C-1), 73.5 (C-6), 73.2 (Ar-*C*H_2), 70.3 (C-2), 68.0 (C-3), 66.4 (C-5), 54.9 (O*C*H_3), 37.1 (C-4), 20.6 (*C*H_3 OAc), 20.4 (*C*H_3 OAc). **HRMS:** Calcd. [$C_{18}H_{24}IO_7$] 479.0561, found 479.0557 (error 0.8 ppm); Calcd. [$C_{18}H_{23}INaO_7$] 501.0381, found 501.0383 (error -0.4 ppm).

1.1.4. Methyl 2,3-di-*O*-acetyl-6-*O*-benzyl-4-deoxy-α-D-*xylo*-hexopyranoside (S4). Compound **S3** (2.19 g, 4.57 mmol) and DIPEA (1.59 mL, 9.14 mmol, 2 equiv.) were dissolved in EtOAc (18 mL) under N_2 atmosphere. Then, 10% Pd/C (10-20% w/w) was carefully added, and the reaction was stirred at room temperature under H_2 atmosphere for 24 hours. The mixture was filtered through a pad of Celite, the solvent was evaporated, and the residue was neutralized

with HCl 2M (100 mL), extracted with DCM (2 x 100 mL), and washed with water (100 mL). Organic phases were combined and dried with $MgSO_4$, filtered, and concentrated under reduced pressure, and the residue was purified by CC (P. Ether/EtOAc 7:1 to 5:1), affording compound **S4** as a colorless oil in 94% yield. R_f (Hex/EtOAc 1:1) = 0.68; **^{1}H NMR** (400.13 MHz, $CDCl_3$, 25 ºC): δ 7.31-7.21 (m, 5H, Ar-*H*), 5.26 (br t, 1H, $J_{3-2} \approx J_{3-4ax}$ = 9.89 Hz, H-3), 4.91 (br s, 1H, H-1), 4.84 (br d, 1H, J_{2-3} = 10.13 Hz, H-2), 4.57-4.47 (m, 2H, Ar-CH_2), 4.04-3.95 (m, 1H, H-5), 3.50-3.43 (m, 2H, H-6), 3.34 (s, 3H, OCH_3), 2.12 (br d, $J_{4eq-4ax}$ = 12.24 Hz, H-4_{eq}), 2.03 (s, 3H, CH_3 OAc); 1.97 (s, 3H, CH_3 OAc), 1.65-1.54 (m, 1H, H-4_{ax}); **^{13}C NMR** (100.62 MHz, $CDCl_3$, 25 ºC): δ 170.2 (C=O OAc), 170.0 (C=O OAc), 137.8 (Ar-C_q), 128.2, 127.5, 127.4 (Ar-*C*H), 97.3 (C-1), 73.2 (Ar-*C*H_2), 71.9 (C-2), 71.7 (C-6), 67.7 (C-3), 66.2 (C-5), 54.9 (O*C*H_3), 32.8 (C-4), 20.8 (*C*H_3 OAc), 20.7 (*C*H_3 OAc). **HRMS:** Calcd. [$C_{18}H_{24}NaO_7$] 375.1414, found 375.1417 (error -0.8 ppm).

1.1.5. Methyl 2,3-di-*O*-acetyl-4-deoxy-α-D-*xylo*-hexopyranoside (S5). Compound **S4** (1.13 g, 3.19 mmol) was dissolved in EtOAc (10 mL) under N_2 atmosphere, after which 10% Pd/C (10-20% w/w) was carefully added. The reaction was stirred at room temperature under H_2 atmosphere for 24 hours. The catalyst was removed via filtration through a pad of Celite, the solvent was evaporated, affording compound **S5** as a colorless oil in 96% yield. R_f (P. Ether/EtOAc 1:1) = 0.23; **^{1}H NMR** (400.13 MHz, $CDCl_3$, 25 ºC): δ 5.29 (dt, 1H, $J_{3-2} \approx J_{3-4ax}$ = 10.17 Hz, J_{3-4eq} = 5.56 Hz, H-3), 4.92 (br s, 1H, H-1), 4.82 (br d, J_{2-3} = 10.20 Hz, H-2), 3.93 (ddd, 1H, J_{5-4ax} = 12.02 Hz, J_{5-6b} = 5.35 Hz, J_{5-4eq} = 2.85 Hz, H-5), 3.66 (d, 1H, J_{6a-6b} = 11.92 Hz, H-6a), 3.54 (dd, 1H, J_{6b-6a} = 12.02 Hz, J_{6b-5} = 5.45 Hz, H-6b), 3.37 (s, 3H, O*C*H_3), 2.13-2.06 (m, 4H, H-4_{eq} and CH_3 OAc), 2.00 (s, 3H, CH_3 OAc), 1.60 (br q, 1H, $J_{4ax-4eq}$ = 12.78 Hz, H-4_{ax}); **^{13}C NMR** (100.62 MHz, $CDCl_3$, 25 ºC): δ 170.7 (C=O OAc), 170.4 (C=O OAc), 97.6 (C-1), 72.2 (C-2), 67.9 (C-3), 67.7 (C-5), 64.9 (C-6), 55.3 (O*C*H_3), 32.2 (C-4), 21.1 (*C*H_3 OAc), 21.0 (*C*H_3 OAc). **HRMS:** Calcd.

$[C_{11}H_{19}O_7]$ 263.1125, found 263.1127 (error -0.6 ppm); Calcd. $[C_{11}H_{18}NaO_7]$ 285.0945, found 285.0946 (error -0.4 ppm).

1.1.6. Methyl 2,3-di-*O*-acetyl-4,6-dideoxy-6-iodo-α-D-*xylo*-hexopyranoside (S6). Compound **S5** (0.80 g, 3.06 mmol) was dissolved in a 2:1 mixture of toluene and ACN (9 mL), together with triphenylphosphine (1.98 g, 7.64 mmol, 2.5 equiv.), imidazole (0.54 g, 7.64 mmol, 2.5 equiv.) and iodine (1.64 g, 6.11 mmol, 2.0 equiv.). The reaction was stirred at 70 ºC for 1 hour, after which the mixture was cooled down to room temperature, neutralized with a saturated solution of sodium thiosulphate (25 mL) and extracted with EtOAc (3 x 25 mL). Organic phases were combined, dried with $MgSO_4$, filtered and the solvent evaporated, and the residue was purified by CC (P. Ether/EtOAc 1:0 to 8:1) to afford compound **S6** as a yellow oil in 91% yield. R_f (Hex/EtOAc 1:1) = 0.74; **^{1}H NMR** (400.13 MHz, $CDCl_3$, 25 ºC): δ 5.29 (dt, 1H, $J_{3\text{-}2} \approx J_{3\text{-}4ax}$ = 10.43 Hz, $J_{3\text{-}4eq}$ = 4.50 Hz, H-3), 4.29 (br s, 1H, H-1), 4.86 (br d, $J_{2\text{-}1}$ = 10.27 Hz, H-2), 3.86-3.80 (m, 1H, H-5), 3.43 (s, 3H, OCH_3), 3.27-3.16 (m, 2H, H-6), 2.33 (ddd, 1H, $J_{4eq\text{-}4ax}$ = 12.48 Hz, $J_{4eq\text{-}3}$ = 4.85 Hz, $J_{4eq\text{-}5}$ = 2.57 Hz, H-4_{eq}), 2.09 (s, 3H, CH_3 OAc), 2.02 (s, 3H, CH_3 OAc), 1.52 (br q, 1H, $J_{4ax\text{-}4eq}$ = 12.53 Hz, H-4_{ax}); **^{13}C NMR** (100.62 MHz, $CDCl_3$, 25 ºC): δ 170.6 (C=O OAc), 170.3 (C=O OAc), 97.7 (C-1), 71.8 (C-2), 67.5 (C-3), 66.6 (C-5), 55.6 (OCH_3), 36.5 (C-4), 21.1 (CH_3 OAc), 21.0 (CH_3 OAc), 7.5 (C-6). **HRMS:** Calcd. $[C_{11}H_{18}IO_6]$ 373.0143, found 373.0142 (error 0.3 ppm); Calcd. $[C_{11}H_{17}INaO_6]$ 394.9962, found 394.9963 (error -0.3 ppm).

1.1.7. Methyl 2,3-di-*O*-acetyl-4,6-dideoxy-α-D-*xylo*-hexopyranoside (S7). Compound **S6** (1.04 g, 2.78 mmol) and DIPEA (0.97 mL, 5.56 mmol, 2 equiv.) were dissolved in EtOAc (10 mL) under N_2 atmosphere, after which 10% Pd/C (10-20% w/w) was carefully added. The reaction was stirred at room temperature under H_2 atmosphere for 24 hours. The catalyst was removed via filtration through a pad of Celite, the solvent was evaporated, and the residue was neutralized with HCl 2M (100 mL), extracted with DCM (2 x 100 mL), and washed with water

(100 mL). Organic phases were combined and dried with $MgSO_4$, filtered, and concentrated under reduced pressure, and the residue was purified by CC (P. Ether/EtOAc 7:1 to 5:1), affording compound **S7** as a colorless oil in 66% yield. R_f (CyHex/EtOAc 2:1) = 0.46; **^{1}H NMR** (400.13 MHz, $CDCl_3$, 25 ºC): δ 5.22 (dt, 1H, $J_{3\text{-}2}$ ≈ $J_{3\text{-}4ax}$ = 11.65 Hz, $J_{3\text{-}4eq}$ = 4.88 Hz, H-3), 4.83-4.79 (m, 2H, H-1 and H-2), 3.98-3.90 (m, 1H, H-5), 3.33 (s, 3H, OCH_3), 2.13 (ddd, 1H, $J_{4eq\text{-}4ax}$ = 12.52 Hz, $J_{4eq\text{-}3}$ = 4.85 Hz, $J_{4eq\text{-}5}$ = 2.40 Hz, H-4_{eq}), 2.05 (s, 3H, CH_3 OAc), 1.98 (s, 3H, CH_3 OAc), 1.41 (br q, 1H, $J_{4ax\text{-}4eq}$ = 12.46 Hz, H-4_{ax}), 1.17 (d, $J_{6\text{-}5}$ = 5.96 Hz, H-6); **^{13}C NMR** (100.62 MHz, $CDCl_3$, 25 ºC): δ 170.6 (C=O OAc), 170.3 (C=O OAc), 97.6 (C-1), 72.2 (C-2), 68.1 (C-3), 63.1 (C-5), 55.1 (OCH_3), 38.2 (C-4), 21.1 (CH_3 OAc), 21.0 (CH_3 OAc), 20.7 (C-6). **HRMS:** Calcd. [$C_{11}H_{19}O_6$] 247.1176, found 247.1180 (error -1.6 ppm); Calcd. [$C_{11}H_{18}NaO_6$] 269.0996, found 269.0997 (error -0.4 ppm).

1.1.8. 1,2,3-tri-*O*-acetyl-4,6-dideoxy-α/β-D-*xylo*-hexopyranoside (11): synthesis from compound **S7**. To a solution of compound **S7** in a 1:1 mixture of acetic acid and acetic anhydride (10 mL) 0 ºC, H_2SO_4 97% (96 µL) was added dropwise. The reaction was stirred at 0 ºC for 1 hour, subsequently washed with a saturated solution of sodium bicarbonate (20 mL) and extracted with DCM (3 x 25 mL). Organic phases were combined and dried with $MgSO_4$, filtered, and concentrated under reduced pressure. CC (P. Ether/EtOAc 5:1) afforded the anomeric mixture (1:0.17 α/β) **11** as a colorless oil in 84% yield. Spectroscopic data is in agreement with the information provided in the experimental section of the main text.

2. Supplementary Tables

2.1. Characterization of clinical isolates

Table S1. Antibiotic susceptibility profile and resistance mechanisms of the used clinical isolates.

	MIC mg/L (RIS[a])							
	**A**<br>*E. coli* ATCC 25922	**B**<br>*E. coli* NCTC 13846	**C**<br>*K. pneumoniae* CQ4921	**D**<br>*E. cloacae* CQ1941	**E**<br>*K. pneumoniae* CQ1942	**F**<br>*K. pneumoniae* CQ1947	**G**<br>*A. baumannii* CQ4322	**H**<br>*P. aeruginosa* CQ4924
**Penicillins**								
Ampicillin	4 (S)	n.d.	≥128 (R)	≥128 (R)	≥128 (R)	≥128 (R)	ERP	ERP
Amoxicillin	4 (S)	n.d.	≥128 (R)	≥128 (R)	≥128 (R)	≥128 (R)	ERP	ERP
Amoxicillin-clavulanic acid	4 (S)	n.d.	≥128 (R)	≥128 (R)	≥128 (R)	≥128 (R)	ERP	ERP
Piperacillin--tazobactam	2-4 (S)	n.d.	≥128 (R)	≥128 (R)	≥128 (R)	32 (R)	n.d.	16 (I)
**Cephalosporins**								
Cefepime	0.03-0.06 (S)	n.d.	n.d.	>64 (R)	>64 (R)	32 (R)	n.d.	n.d.
Cefotaxime	0.06 (S)	n.d.	2 (I/R)	≥128 (R)	≥128 (R)	≥128 (R)	ERP	ERP
Ceftazidime	0.125-0.25 (S)	n.d.	1 (S)	≥128 (R)	≥128 (R)	≥128 (R)	n.d.	4 (I)
**Carbapenems**								
Imipenem	0.125-0.25 (S)	n.d.	4 (I)	16 (R)	≥128 (R)	8 - 16 (R)	32-64 (R)	16 (R)
Meropenem	0.016-0.03 (S)	n.d.	4 (R)	32 - 64 (R)	≥128 (R)	16 - 32 (R)	64-≥128 (R)	16 (R)
Ertapenem	0.008 (S)	n.d.	8 - 64 (R)	64 (R)	≥128 (R)	16 - 64 (R)	ERP	ERP
Resistance Mechanism	neg	neg	OXA-48	NDM-1	KPC-3	IMP-1	GES-12	neg
**Monobactams**								
Aztreonam	0.125 (S)	n.d.	n.d.	>64 (R)	>64 (R)	0.25 (S)	ERP	n.d.
**Fluoroquinolones**								
Ciprofloxacin	0.008 (S)	n.d.	0.03 (S)	≥128 (R)	≥128 (R)	≥128 (R)	64-≥128 (R)	8-16 (R)
**Aminoglycosides**								
Amikacin	1-2 (S)	n.d.	0.5 - 2 (S)	≥128 (R)	16 (R)	32 (R)	≥128 (R)	4 (S)
Gentamicin	0.5 (S)	n.d.	0.25 - 0.5 (S)	≥128 (R)	1 (S)	64 (R)	32-64 (R)	2 (wt)
Tobramycin	0.5 (S)	n.d.	0.25 (S)	≥128 (R)	16 (R)	≥128 (R)	32 (R)	1 (S)
**Polymyxins**								
Colistin	0.5-1 (S)	4 (R)	0.125-1 (S)	0.125-1 (S)	0.125-1 (S)	0.125-1 (S)	0.5-1 (S)	1-2 (S)
Resistance Mechanism	neg	MCR-1	neg	neg	neg	neg	neg	neg

[a]MIC interpretation accordingly with EUCAST clinical breakpoints (S - Susceptible, standard dosing regimen; I - Susceptible, increased exposure; R - Resistant. ERP: Expected resistant phenotype), or EUCAST epidemiological cut-off values (ECOFF) (wt – wild-type). neg: negative for acquired resistance mechanisms. n.d.: not determined.

2.2. MIC values (Antimicrobial Activity Assays)

Table S2. MIC (µg/mL) values for colistin in the combination of colistin with the compound against several critical priority carbapenem-resistant Gram-negative clinical isolates.

Cpd	Cpd conc. (µg/mL)	A<br>*E. coli*<br>ATCC 25922	B<br>*E. coli*<br>NCTC 13846	C<br>*K. pneumoniae*<br>CQ4921	D<br>*E. cloacae*<br>CQ1941	E<br>*K. pneumoniae*<br>CQ1942	F<br>*K. pneumoniae*<br>CQ1947	G<br>*A. baumannii*<br>CQ4322	H<br>*P. aeruginosa*<br>CQ4924
**1**	**0.4**	> 0.5	> 0.5	> 0.5	> 0.5	> 0.5	> 0.5	> 0.5	> 0.5
	**0.8**	> 0.5	> 0.5	> 0.5	0.5	0.5	> 0.5	> 0.5	> 0.5
	**1.6**	> 0.5	> 0.5	0.5	0.25	0.5	> 0.5	0.5	> 0.5
	**3.125**	0.25	> 0.5	0.25	0.25	0.25	0.5	0.25	> 0.5
	**6.25**	0.25	> 0.5	0.25	0.25	0.25	0.5	0.25	> 0.5
	**12.5**	0.25	> 0.5	0.25	0.25	0.25	0.5	0.25	> 0.5
**2**	**0.4**	> 0.5	> 0.5	0.5	0.5	0.5	> 0.5	> 0.5	> 0.5
	**0.8**	0.5	> 0.5	0.5	0.5	0.5	0.5	0.5	> 0.5
	**1.6**	0.5	> 0.5	0.5	0.25	0.5	0.5	0.5	> 0.5
	**3.125**	≤ 0.06	> 0.5	0.125	0.25	0.5	0.5	0.25	> 0.5
	**6.25**	≤ 0.06	> 0.5	0.125	0.25	0.25	0.5	0.125	0.5
	**12.5**	≤ 0.06	> 0.5	0.125	0.125	0.25	0.5	0.125	0.5
**3**	**0.4**	0.5	> 0.5	0.5	0.5	0.5	0.5	0.5	> 0.5
	**0.8**	0.5	> 0.5	0.5	0.5	0.5	0.5	0.5	> 0.5
	**1.6**	0.5	> 0.5	0.5	0.25	0.5	0.5	0.5	> 0.5
	**3.125**	0.25	> 0.5	0.25	0.25	0.25	0.5	0.5	0.5
	**6.25**	0.25	> 0.5	0.25	0.25	0.25	0.5	0.25	0.5
	**12.5**	0.25	> 0.5	0.25	0.25	0.25	0.5	0.25	0.5
**4**	**0.4**	0.5	> 0.5	0.5	0.5	0.5	0.5	0.5	> 0.5
	**0.8**	0.5	> 0.5	0.5	0.5	0.5	0.5	0.5	> 0.5
	**1.6**	0.5	> 0.5	0.5	0.5	0.5	0.5	0.5	> 0.5
	**3.125**	0.5	> 0.5	0.5	0.5	0.5	0.5	0.5	> 0.5
	**6.25**	0.5	> 0.5	0.5	0.5	0.5	0.5	0.5	> 0.5
	**12.5**	0.5	> 0.5	0.5	0.5	0.5	0.5	0.5	> 0.5
**5**	**0.4**	> 0.5	> 0.5	0.5	0.5	0.5	0.5	> 0.5	> 0.5
	**0.8**	> 0.5	> 0.5	0.5	0.5	0.5	0.5	0.5	> 0.5
	**1.6**	0.5	> 0.5	0.5	0.5	0.5	0.5	0.5	> 0.5
	**3.125**	0.25	> 0.5	0.125	0.125	0.25	0.25	0.25	0.5
	**6.25**	0.125	> 0.5	0.125	0.125	0.25	0.25	0.25	0.5
	**12.5**	≤ 0.06	> 0.5	0.125	0.125	0.25	0.25	0.25	0.5

Cpd: compound.

Table S3. MIC (µg/mL) values for compound in the combination of compound + colistin against several critical priority carbapenem-resistant Gram-negative clinical isolates. Results are presented as the median of three independent replicates.

Cpd	Colistin conc. (µg/mL)	**A** *E. coli* ATCC 25922	**B** *E. coli* NCTC 13846	**C** *K. pneumoniae* CQ4921	**D** *E. cloacae* CQ1941	**E** *K. pneumoniae* CQ1942	**F** *K. pneumoniae* CQ1947	**G** *A. baumannii* CQ4322	**H** *P. aeruginosa* CQ4924
**1**	0.5	3.1	>100	1.6	0.8	0.8	3.1	1.6	>100
	0.25	3.1	>100	3.1	1.6	3.1	>100	3.1	>100
	0.125	>100	>100	>100	>100	>100	>100	>100	>100
	0.06	>100	>100	>100	>100	>100	>100	>100	>100
	0	>100	>100	>100	>100	>100	>100	>100	>100
**2**	0.5	0.4	>100	≤0.1	≤0.1	≤0.1	0.8	0.8	6.3
	0.25	3.1	>100	3.1	1.6	6.3	>100	3.1	>100
	0.125	3.1	>100	3.1	12.5	>100	>100	6.3	>100
	0.06	3.1	>100	>100	>100	>100	>100	>100	>100
	0	>100	>100	>100	>100	>100	>100	>100	>100
**3**	0.5	≤0.1	>100	0.2	≤0.1	≤0.1	≤0.1	≤0.1	3.1
	0.25	3.1	>100	3.1	1.6	3.1	>100	6.3	>100
	0.125	>100	>100	>100	>100	>100	>100	>100	>100
	0.06	>100	>100	>100	>100	>100	>100	>100	>100
	0	>100	>100	>100	>100	>100	>100	>100	>100
**4**	0.5	≤0.1	>100	≤0.1	≤0.1	≤0.1	≤0.1	≤0.1	>100
	0.25	>100	>100	>100	>100	>100	>100	>100	>100
	0	>100	>100	>100	>100	>100	>100	>100	>100
**5**	0.5	1.6	>100	≤0.1	≤0.1	≤0.1	0.2	0.8	3.1
	0.25	3.1	>100	3.1	3.1	3.1	3.1	3.1	>100
	0.125	6.3	>100	3.1	3.1	>100	>100	25	>100
	0.06	12.5	>100	>100	>100	>100	>100	>100	>100
	0	>100	>100	>100	>100	>100	>100	>100	>100

Cpd: compound.

Table S4. MIC (µg/mL) values for compound in the combination of compound + PMB against several critical priority carbapenem-resistant Gram-negative clinical isolates. Results are presented as the median of three independent replicates.

Cpd	PMB conc. (µg/mL)	**A** *E. coli* ATCC 25922	**B** *E. coli* NCTC 13846	**C** *K. pneumoniae* CQ4921	**D** *E. cloacae* CQ1941	**E** *K. pneumoniae* CQ1942	**F** *K. pneumoniae* CQ1947	**G** *A. baumannii* CQ4322	**H** *P. aeruginosa* CQ4924
**1**	0.5	3.1	>100	1.6	1.6	1.6	3.1	3.1	>100
	0.25	>100	>100	>100	>100	>100	>100	>100	>100
	0	>100	>100	>100	>100	>100	>100	>100	>100
**2**	0.5	0.8	>100	≤0.1	≤0.1	≤0.1	0.8	0.8	6.3
	0.25	3.1	>100	6.3	12.5	>100	>100	6.3	>100
	0	>100	>100	>100	>100	>100	>100	>100	>100
**3**	0.5	≤0.1	>100	≤0.1	≤0.1	≤0.1	1.6	0.8	>100
	0.25	3.1	>100	6.3	6.3	>100	>100	>100	>100
	0	>100	>100	>100	>100	>100	>100	>100	>100
4	0.5	≤0.1	>100	1.6	≤0.1	1.6	>100	6.3	>100
	0.25	>100	>100	>100	>100	>100	>100	>100	>100
	0	>100	>100	>100	>100	>100	>100	>100	>100
**5**	0.5	3.1	>100	1.6	1.6	1.6	3.1	3.1	6.3
	0.25	3.1	>100	>100	>100	>100	>100	>100	>100
	0	>100	>100	>100	>100	>100	>100	>100	>100

Cpd: compound; PMB: Polymyxin B.

2.3. IC_{50} values (Cytotoxicity Assays)

Table S5. Cytotoxicity of compound combinations with polymyxins in HEK-293T cells. Results are presented as the mean ± standard deviation of three independent replicates. n.d.: not determined. Cpd: Compound.

IC_{50} (μM)							
Cpd	No polymyxin	PMB 0.5 μg/mL	PMB 0.25 μg/mL	PMB 0.125 μg/mL	Colistin 0.5 μg/mL	Colistin 0.25 μg/mL	Colistin 0.125 μg/mL
**1**	65.77 ± 1.28	67.67 ± 1.22	65.32 ± 0.40	64.63 ± 0.87	65.87 ± 3.75	69.41 ± 2.58	n.d.
**2**	84.17 ± 2.59	81.96 ± 2.90	84.49 ± 1.52	n.d.	84.14 ± 2.87	80.73 ± 6.08	n.d.
**3**	72.47 ± 2.51	75.10 ± 2.70	n.d.	n.d.	79.33 ± 2.00	n.d.	n.d.
**4**	>100	>100	n.d.	n.d.	>100	n.d.	n.d.
**5**	79.74 ± 0.53	83.18 ± 0.96	79.44 ± 1.58	79.19 ± 2.00	80.05 ± 1.69	78.57 ± 3.28	79.74 ± 1.05

Table S6. Cytotoxicity of compound combinations with polymyxins in Caco-2 cells. Results are presented as the mean ± standard deviation of three independent replicates. n.d.: not determined. Cpd: Compound.

IC_{50} (μM)							
Cpd	No polymyxin	PMB 0.5 μg/mL	PMB 0.25 μg/mL	PMB 0.125 μg/mL	Colistin 0.5 μg/mL	Colistin 0.25 μg/mL	Colistin 0.125 μg/mL
**1**	54.25 ± 2.77	52.21 ± 1.77	50.50 ± 0.15	n.d.	52.46 ± 2.28	54.72 ± 1.04	n.d.
**2**	54.82 ± 0.61	56.44 ± 3.90	58.79 ± 2.66	n.d.	53.42 ± 5.35	58.09 ± 0.33	55.7 ± 0.52
**3**	68.86 ± 2.21	n.d.	n.d.	n.d.	68.18 ± 2.12	69.20 ± 1.25	n.d.
**4**	>100	n.d.	n.d.	n.d.	>100	n.d.	n.d.
**5**	62.39 ± 1.22	n.d.	61.07 ± 1.06	n.d.	64.11 ± 1.09	64.87 ± 0.98	n.d.

Compound Characterization
3.1. NMR spectra

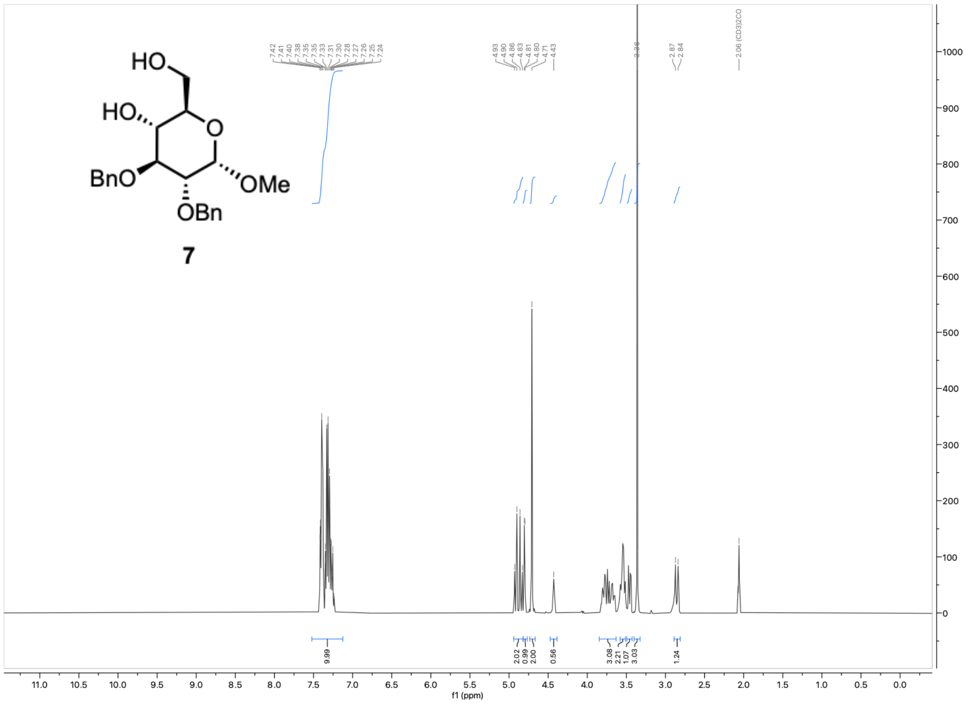

Figure S2. ^{1}H spectrum of compound **7** in Acetone-d_6.

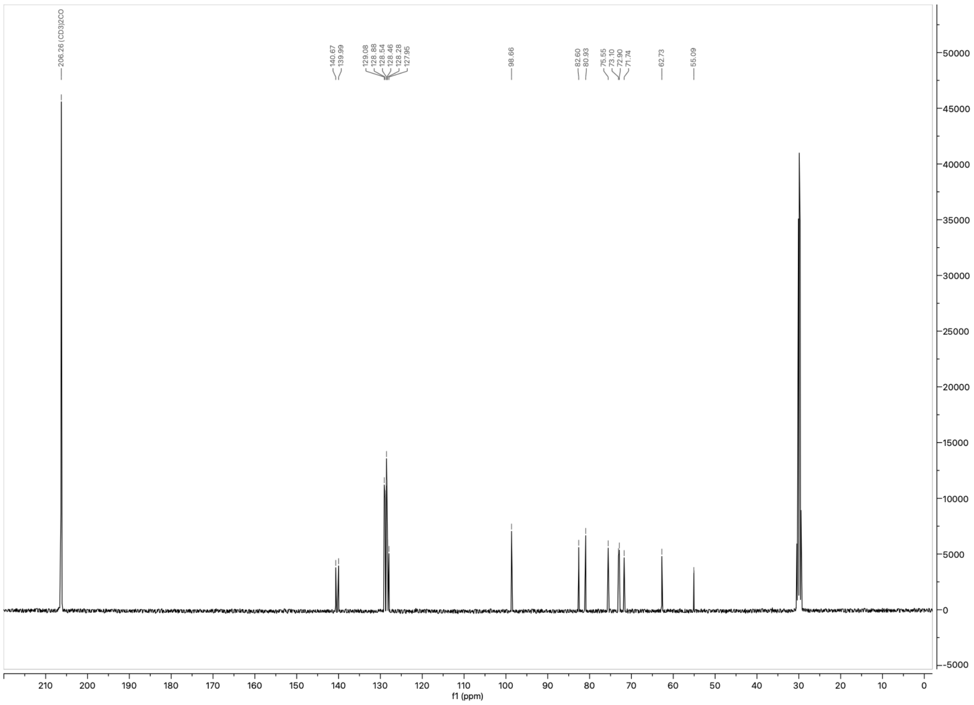

Figure S3. ^{13}C spectrum of compound **7** in Acetone-d_6.

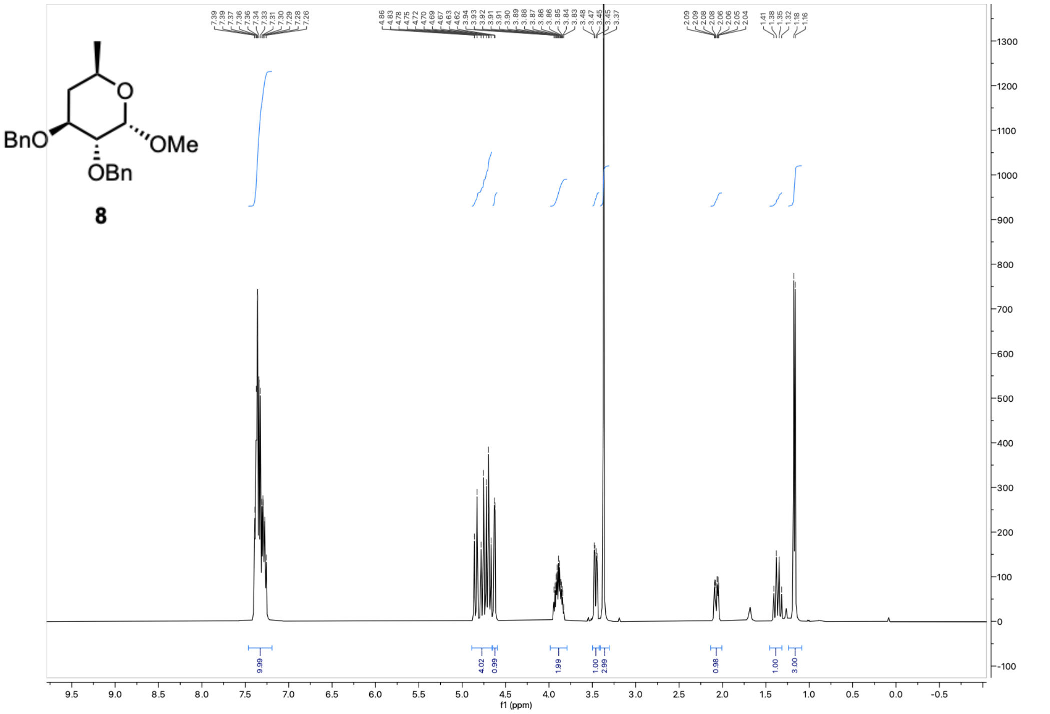

Figure S4. ^{1}H spectrum of compound **8** in Chloroform-d.

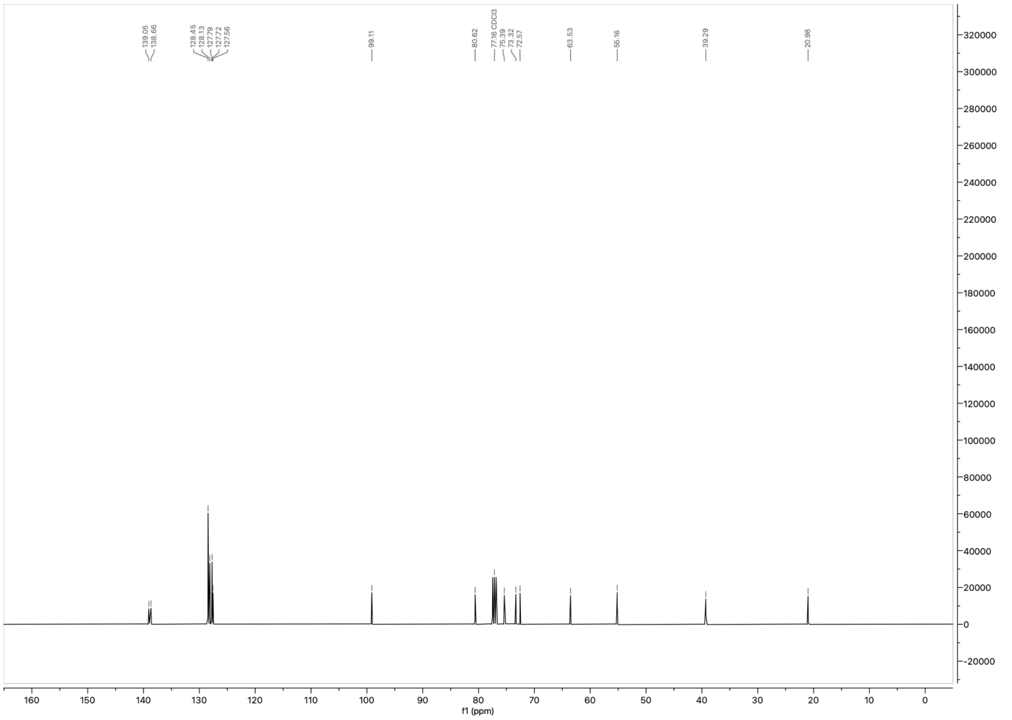

Figure S5. ^{13}C spectrum of compound **8** in Chloroform-d.

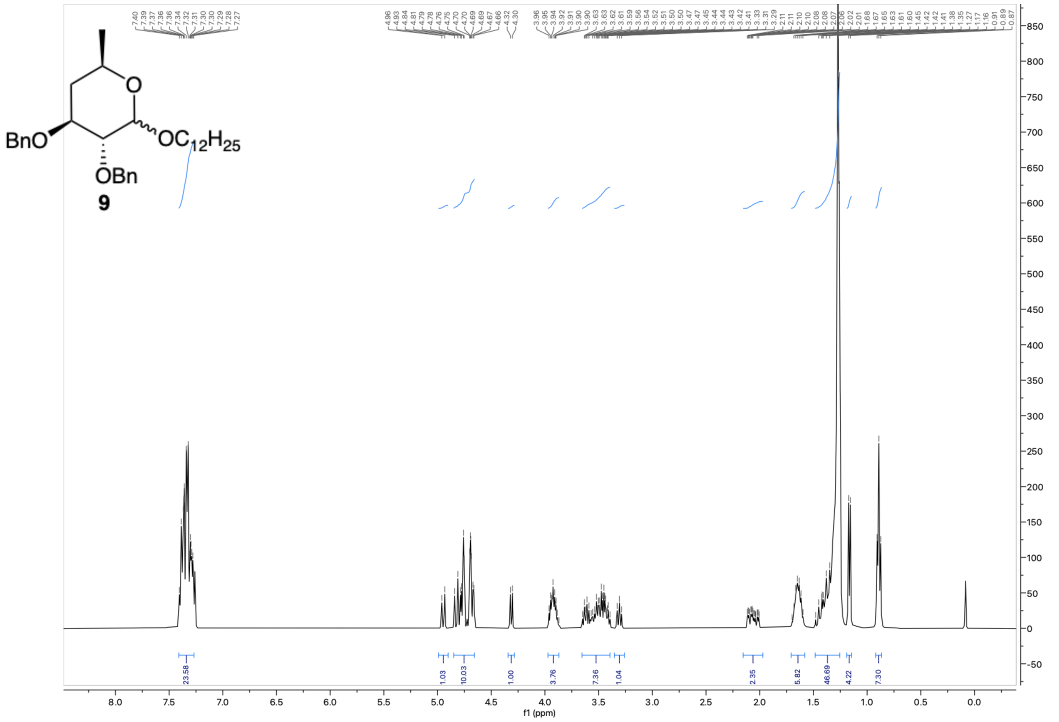

Figure S6. ^{1}H spectrum of compound **9** in Chloroform-d.

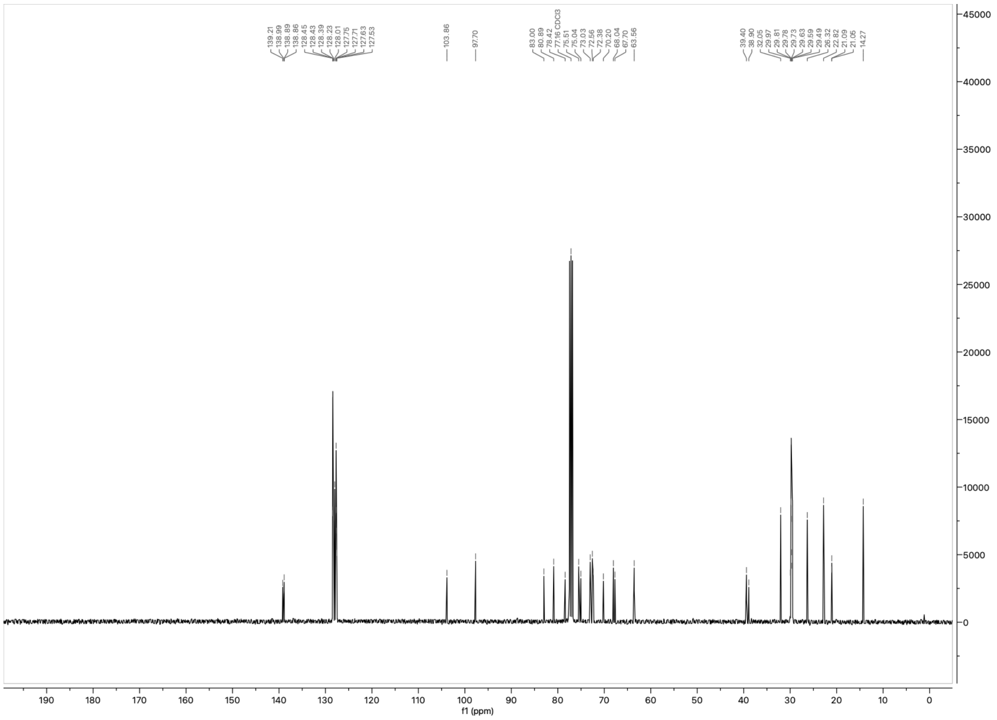

Figure S7. ^{13}C spectrum of compound **9** in Chloroform-d.

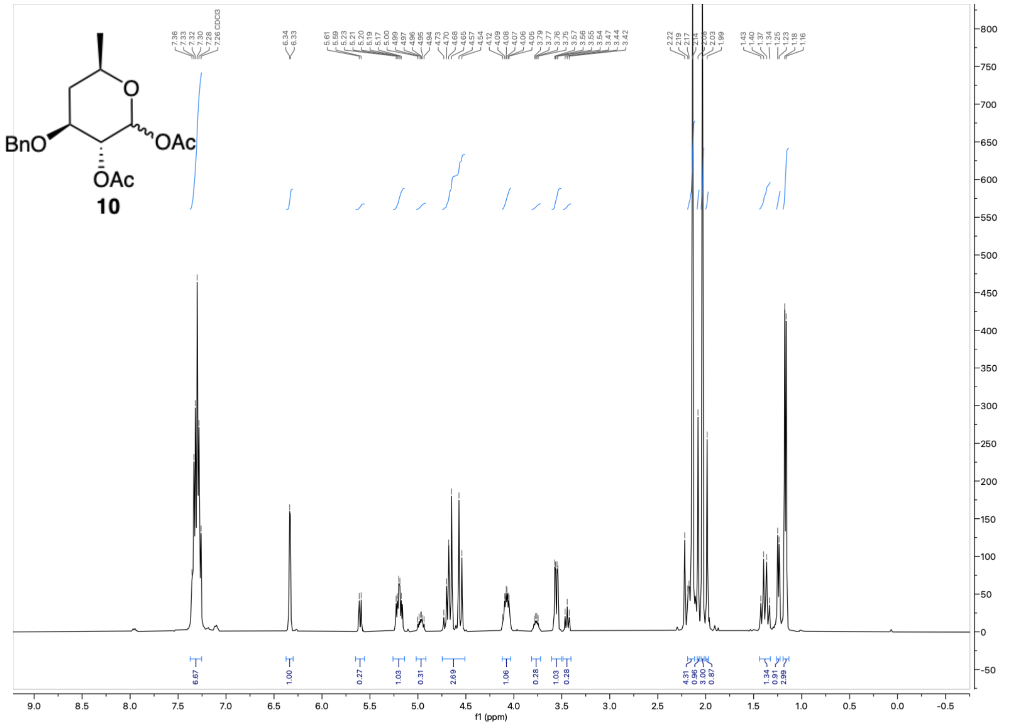

Figure S8. ^{1}H spectrum of compound **10** in Chloroform-d.

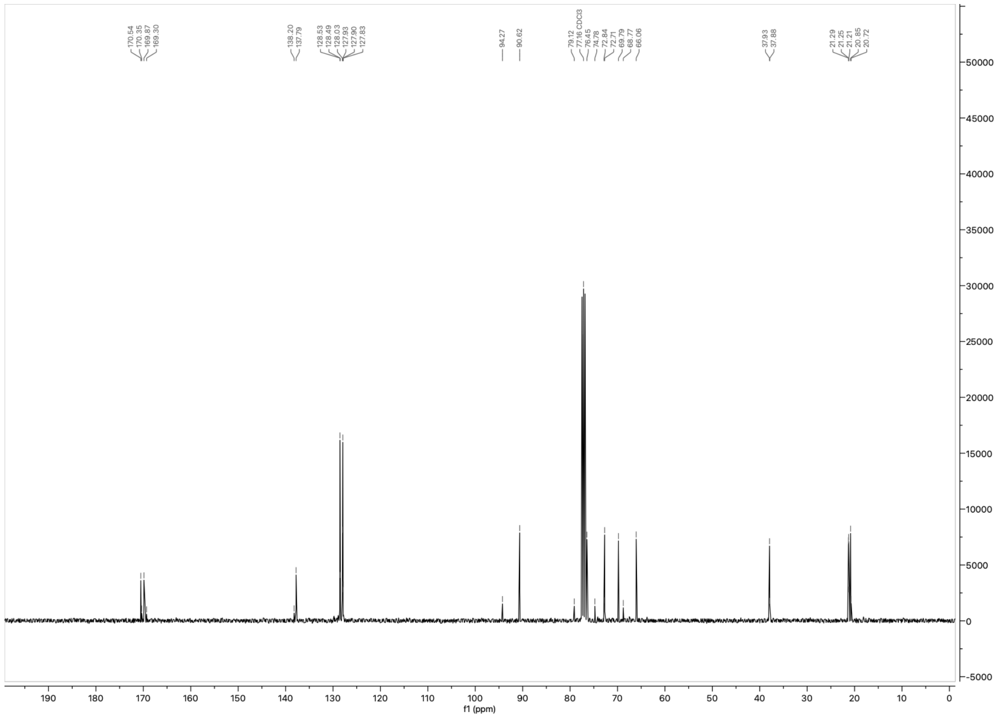

Figure S9. ^{13}C spectrum of compound **10** in Chloroform-d.

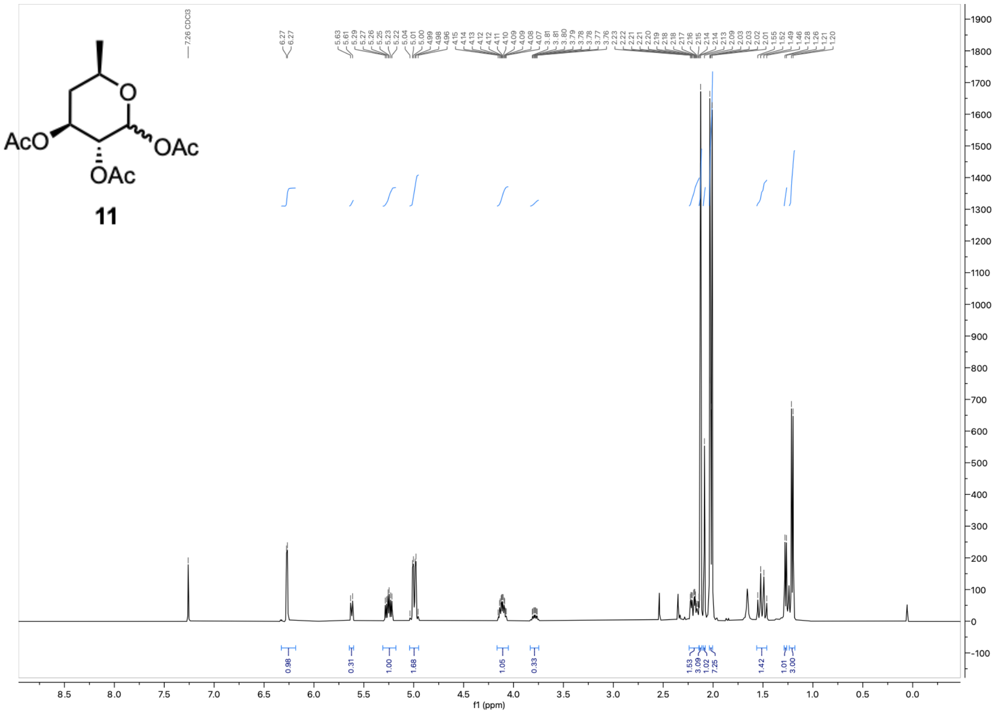

Figure S10. ^{1}H spectrum of compound **11** in Chloroform-d.

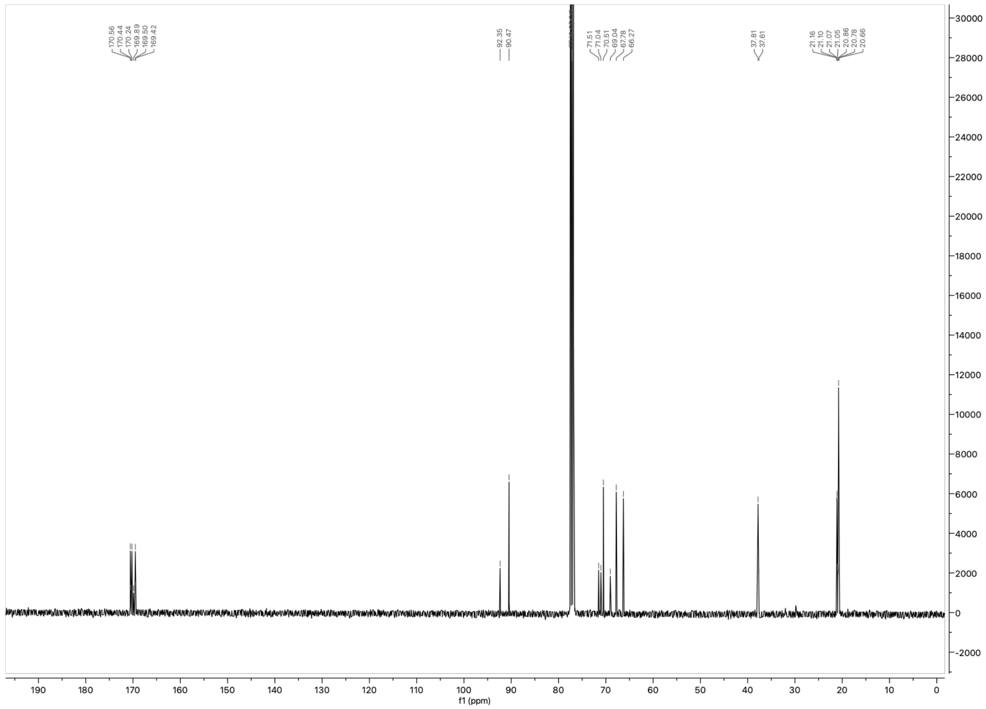

Figure S11. ^{13}C spectrum of compound **11** in Chloroform-d.

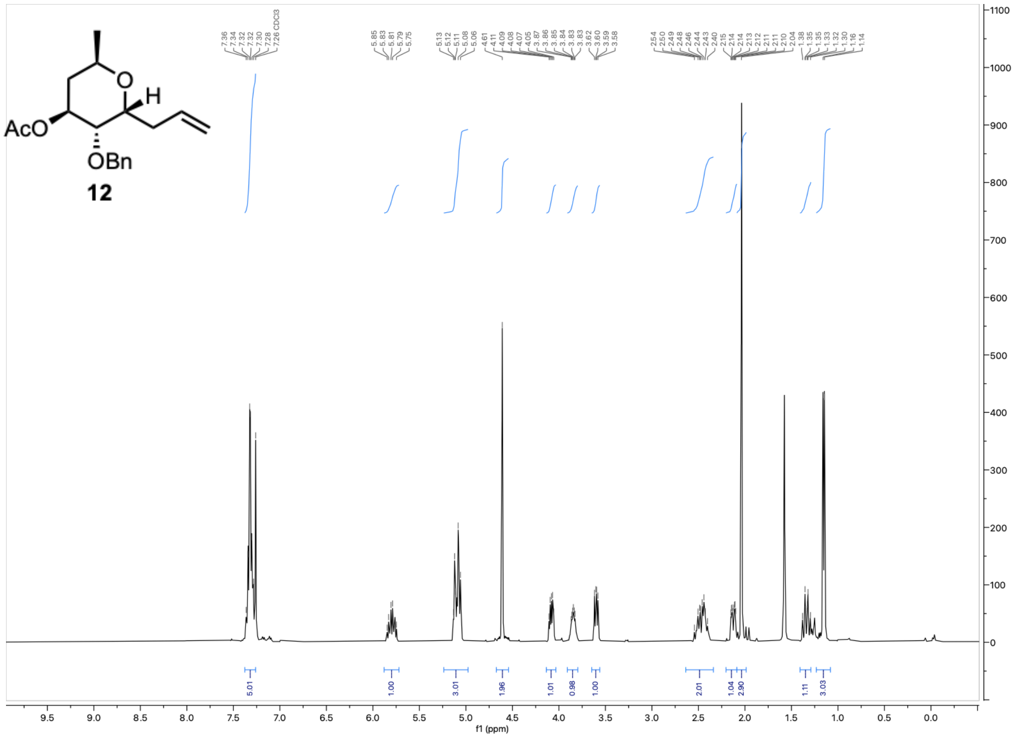

Figure S12. ^{1}H spectrum of compound **12** in Chloroform-d.

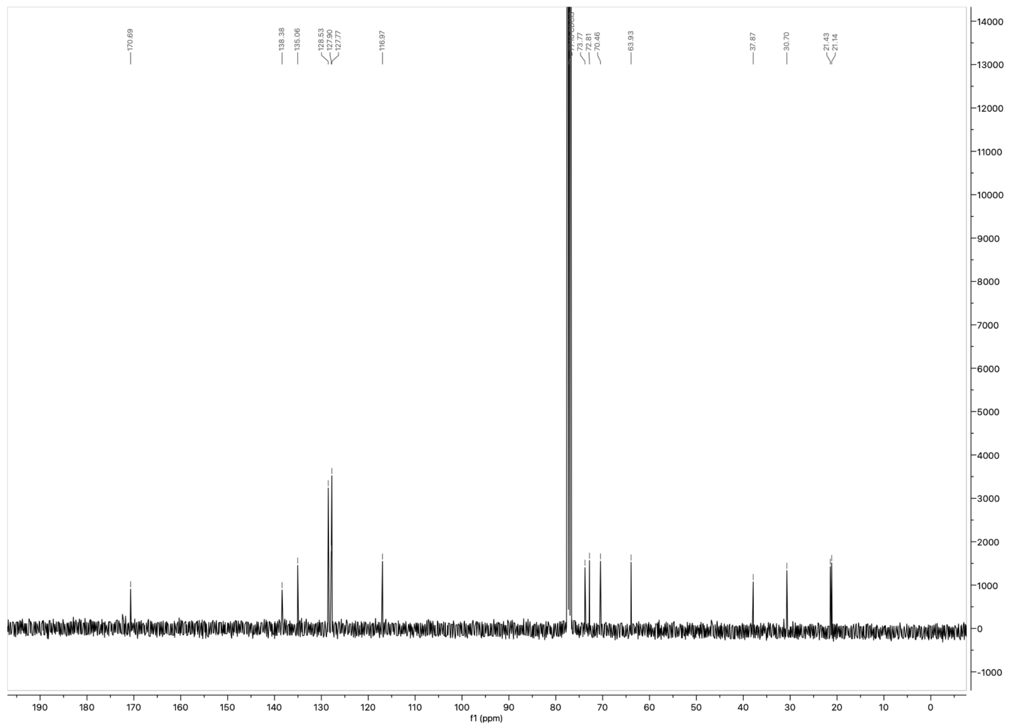

Figure S13. ^{13}C spectrum of compound **12** in Chloroform-d.

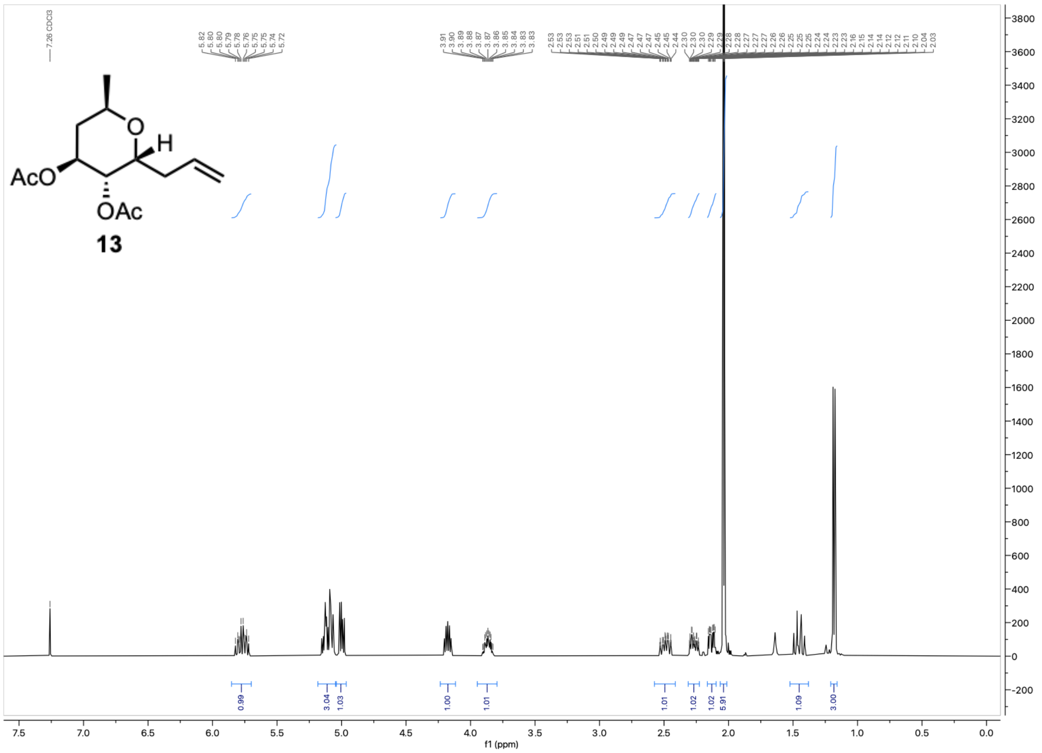

Figure S14. ^{1}H spectrum of compound **13** in Chloroform-d.

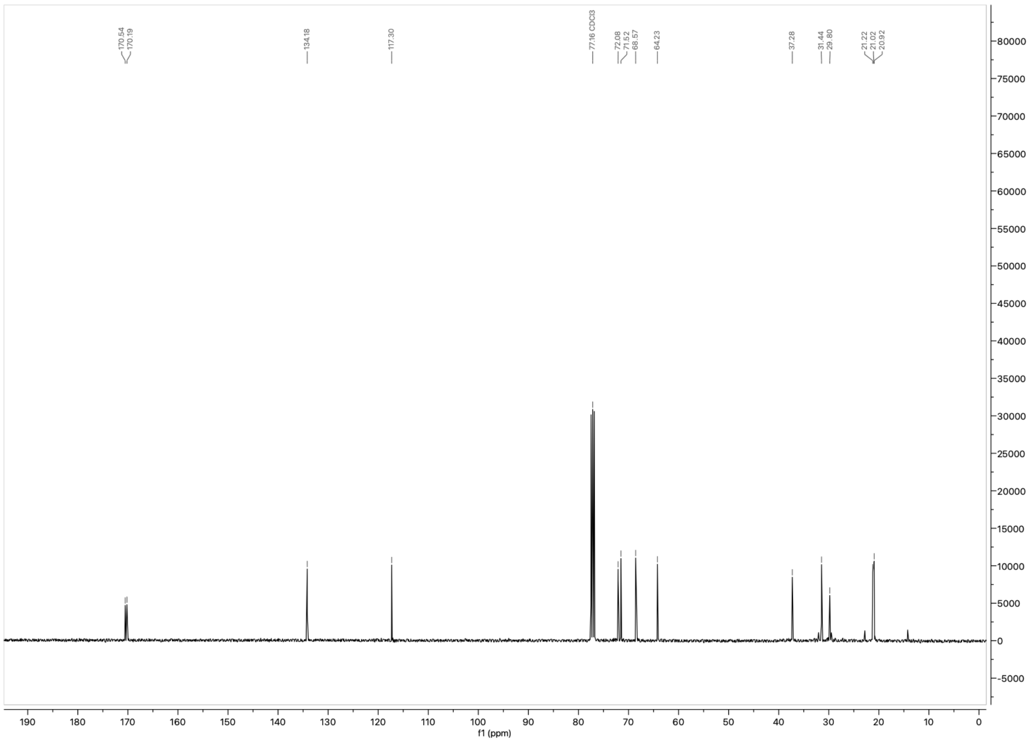

Figure S15. ^{13}C spectrum of compound **13** in Chloroform-d.

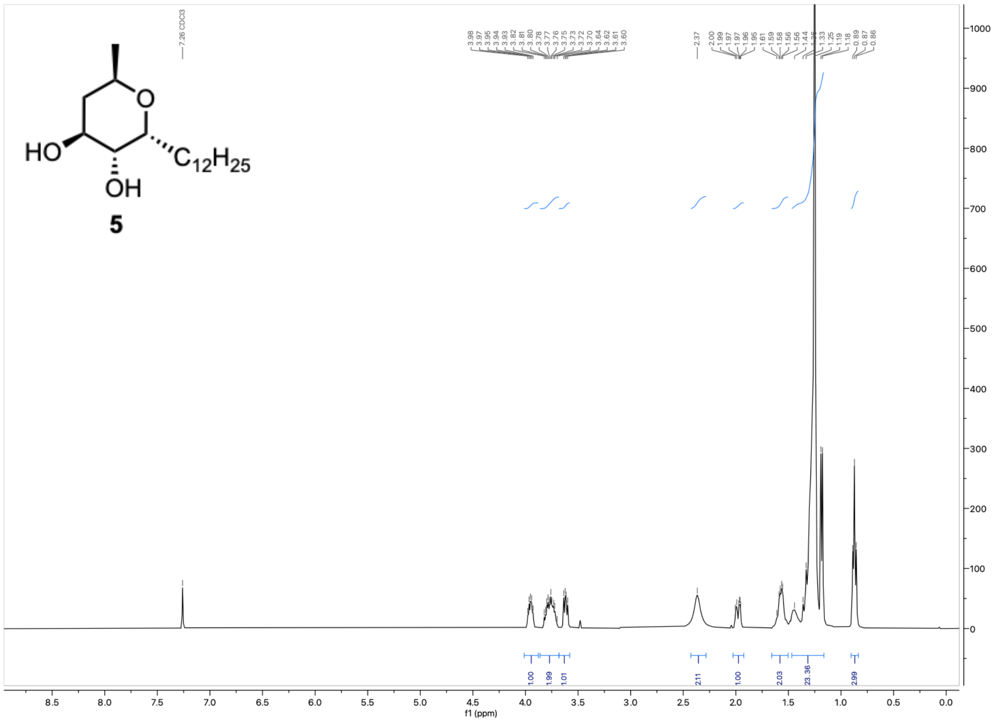

Figure S16. ^{1}H spectrum of compound **5** in Chloroform-d.

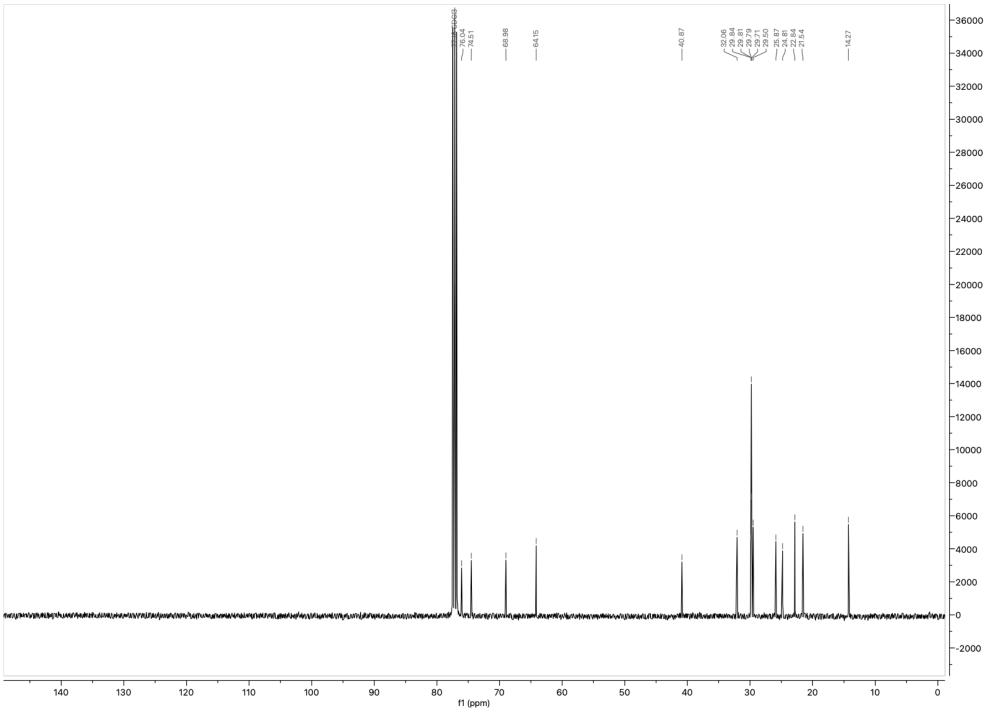

Figure S17. ^{13}C spectrum of compound **5** in Chloroform-d.

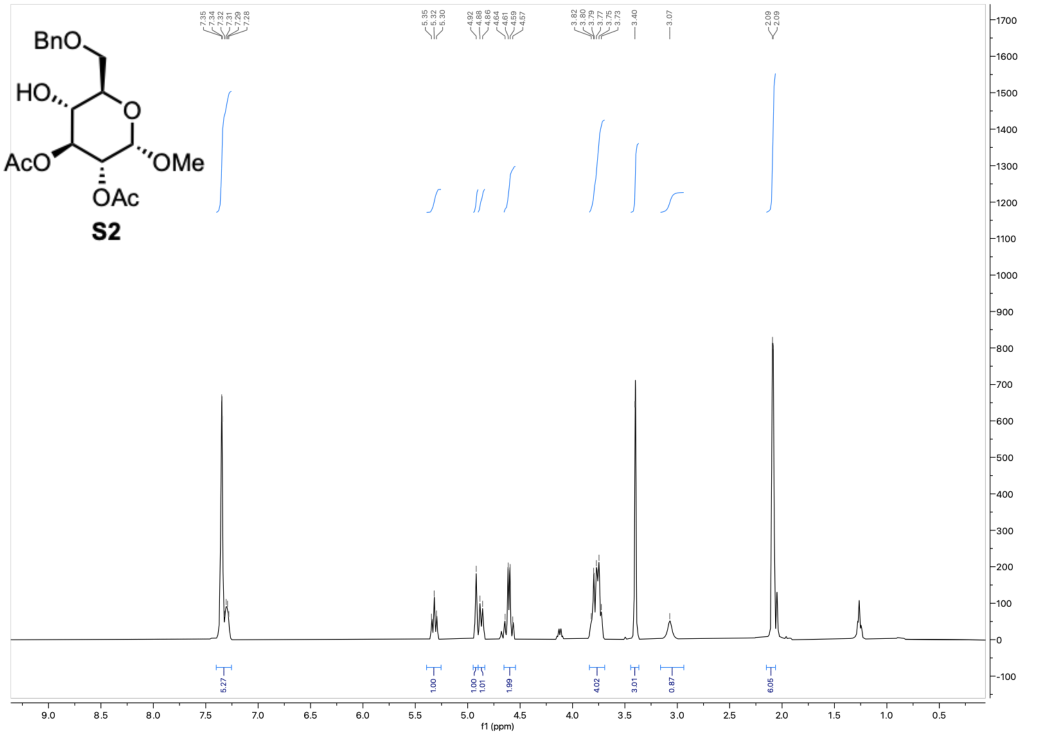

Figure S18. ^{1}H spectrum of compound **S2** in Chloroform-d.

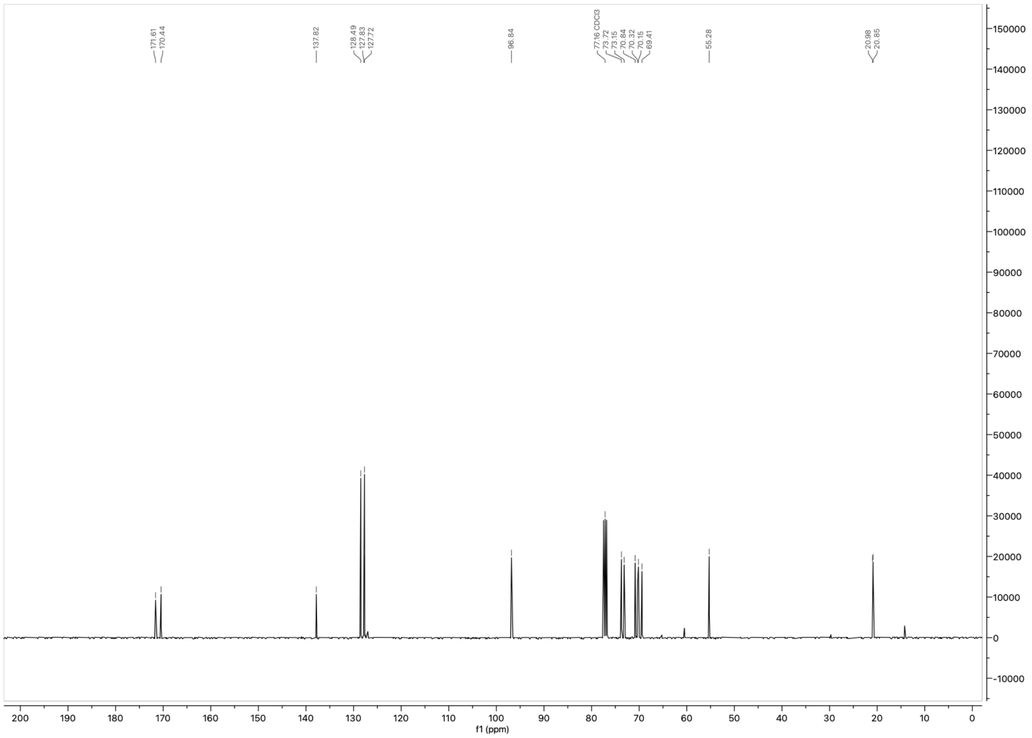

Figure S19. ^{13}C spectrum of compound **S2** in Chloroform-d.

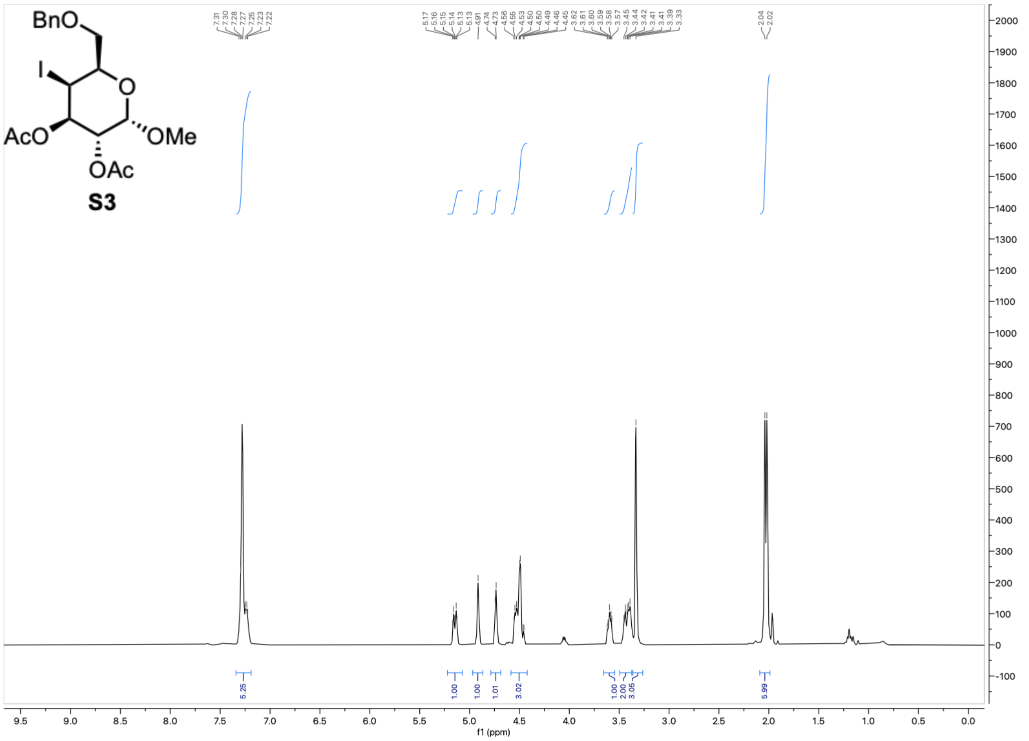

Figure S20. ^{1}H spectrum of compound **S3** in Chloroform-d.

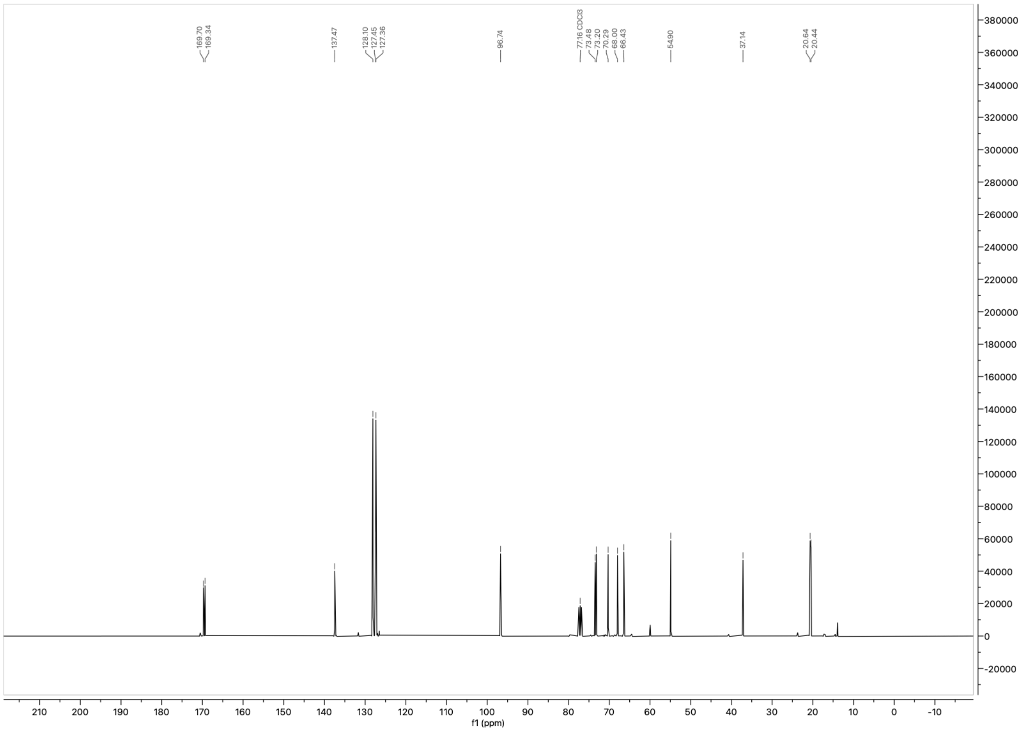

Figure S21. ^{13}C spectrum of compound **S3** in Chloroform-d.

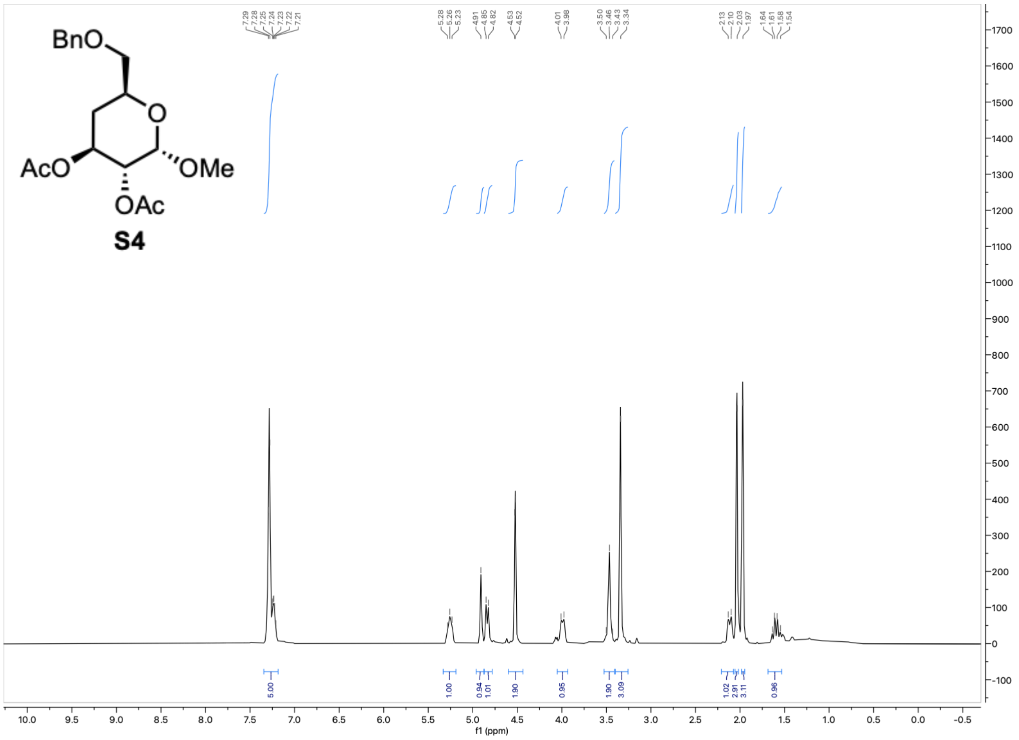

Figure S22. ^{1}H spectrum of compound **S4** in Chloroform-d.

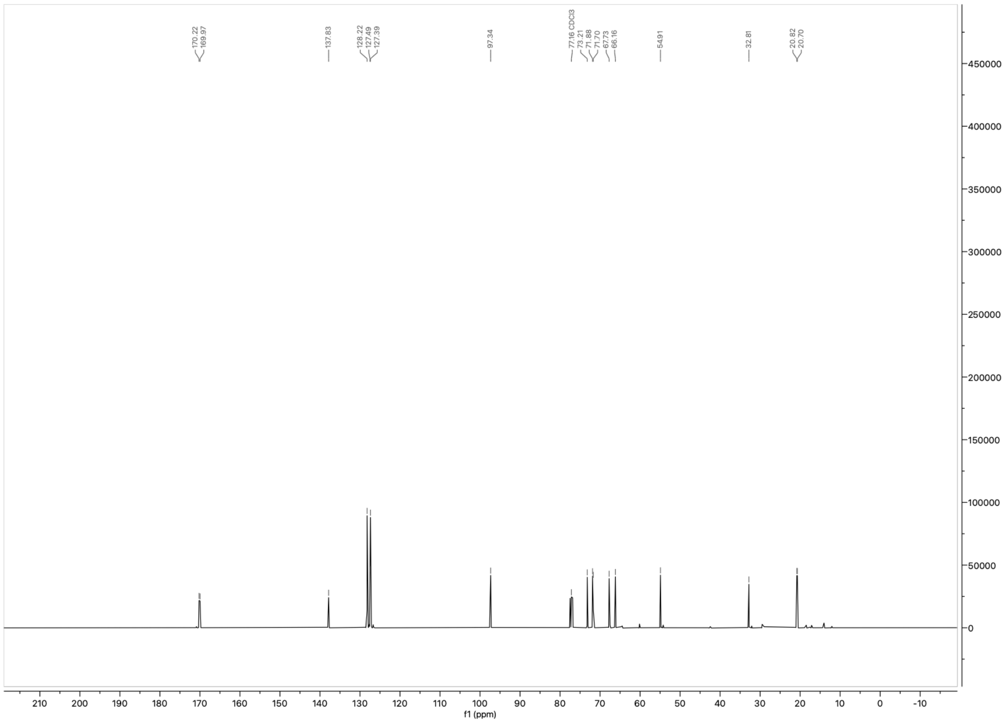

Figure S23. ^{13}C spectrum of compound **S4** in Chloroform-d.

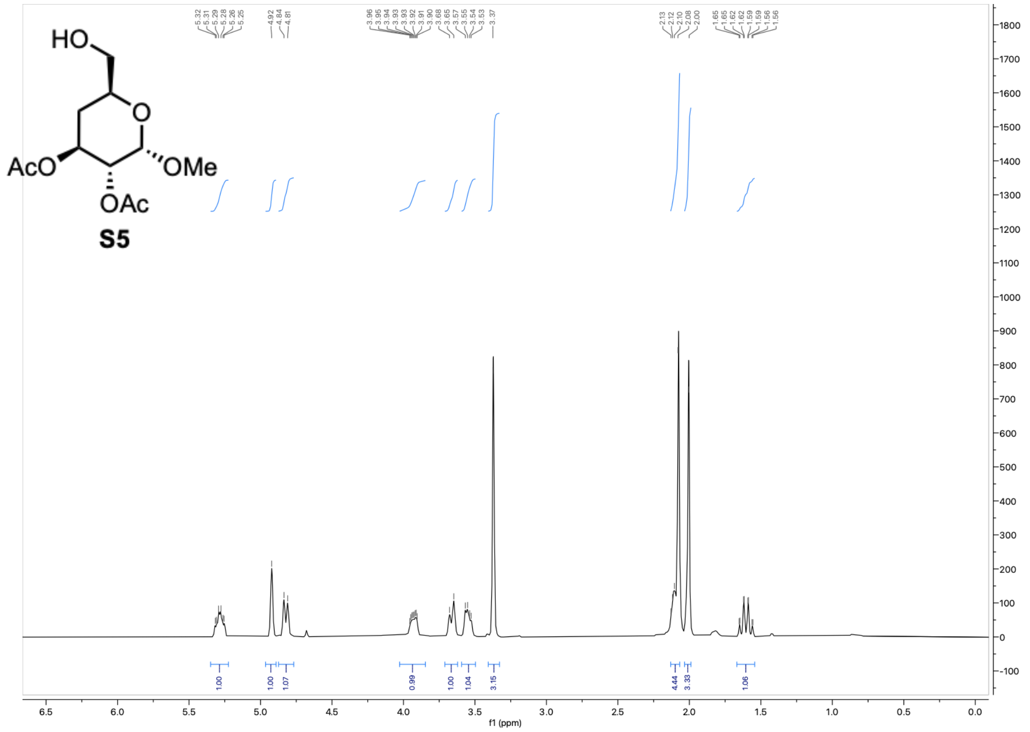

Figure S24. ^{1}H spectrum of compound **S5** in Chloroform-d.

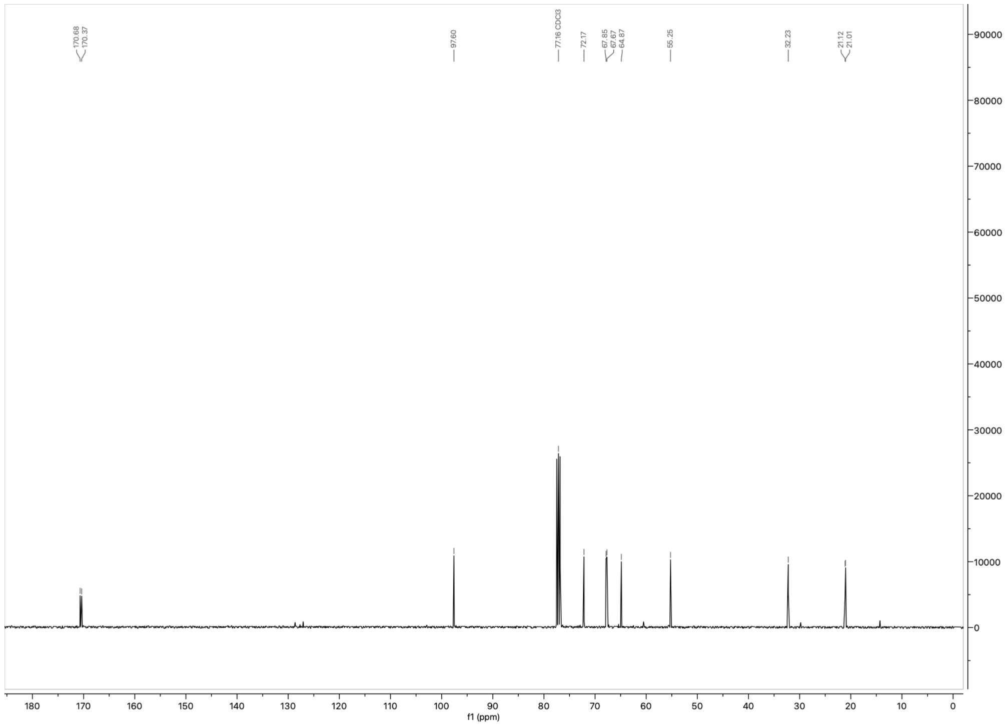

Figure S25. ^{13}C spectrum of compound **S5** in Chloroform-d.

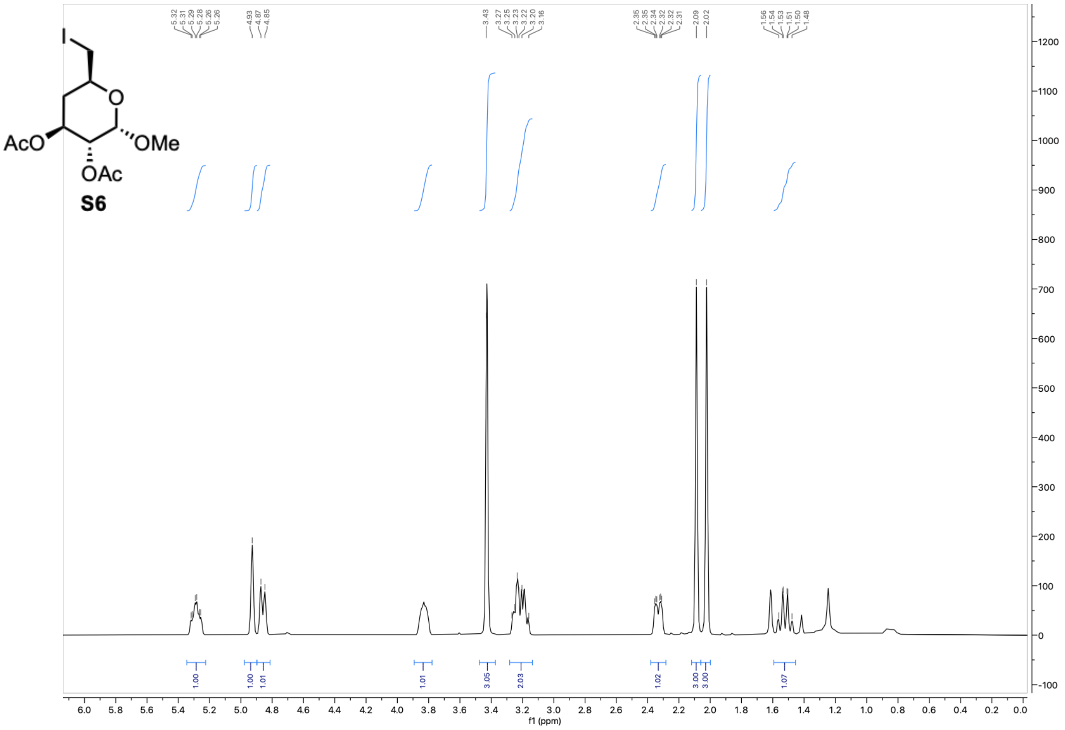

Figure S26. ^{1}H spectrum of compound **S6** in Chloroform-d.

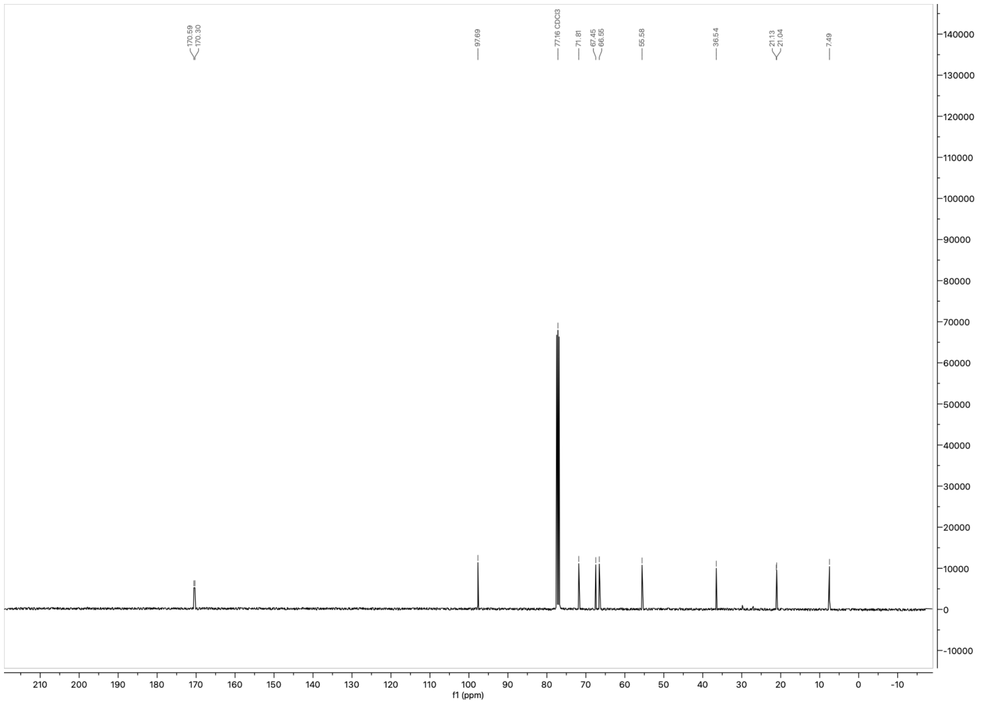

Figure S27. ^{13}C spectrum of compound **S6** in Chloroform-d.

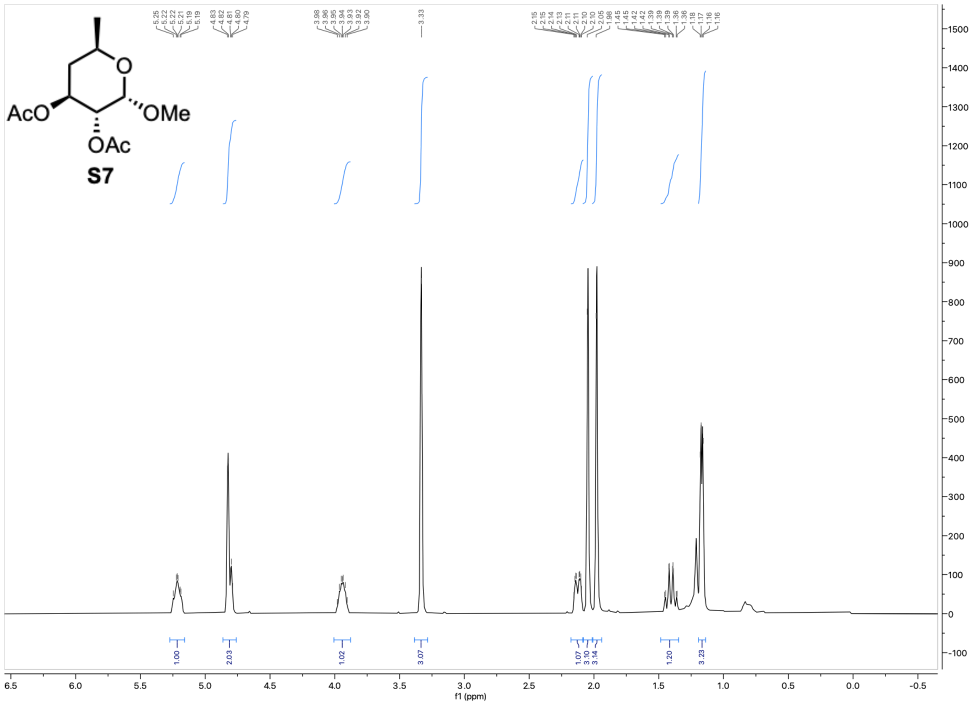

Figure S28. ^{1}H spectrum of compound **S7** in Chloroform-d.

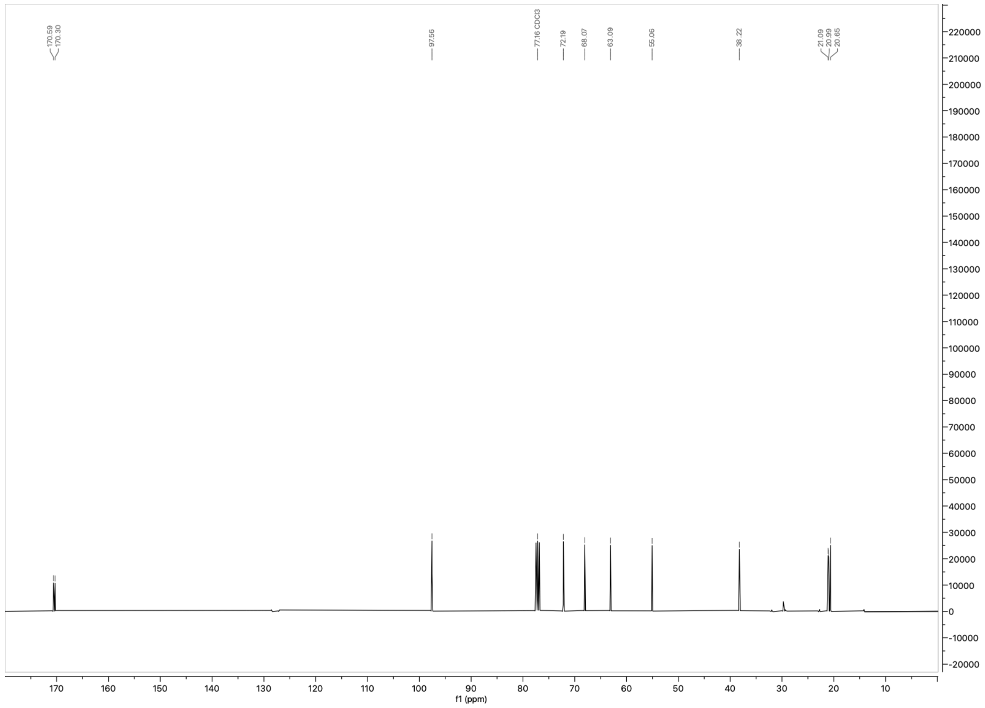

Figure S29. ^{13}C spectrum of compound **S7** in Chloroform-d.

3.2. HRMS Spectra

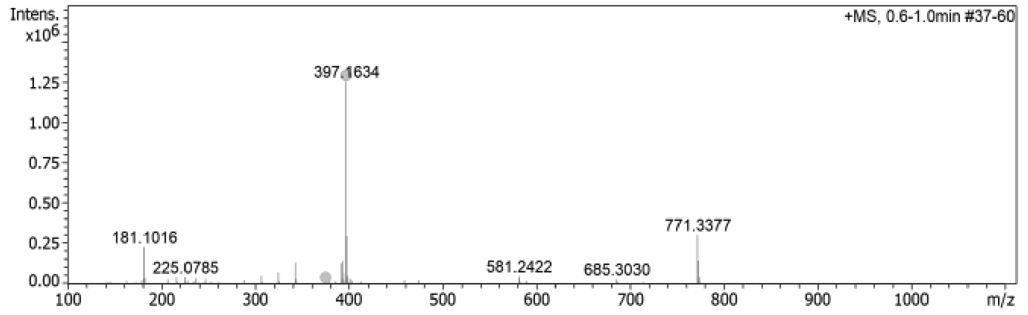

Meas. m/z	#	Ion Formula	m/z	err [ppm]	mSigma	# mSigma	Score	rdb	e⁻ Conf	N-Rule
375.1810	1	C21H27O6	375.1802	-2.0	35.5	1	100.00	9.0	even	ok
397.1634	1	C21H26NaO6	397.1622	-3.0	4.3	1	100.00	9.0	even	ok

Figure S30. HRMS results for compound **7**.

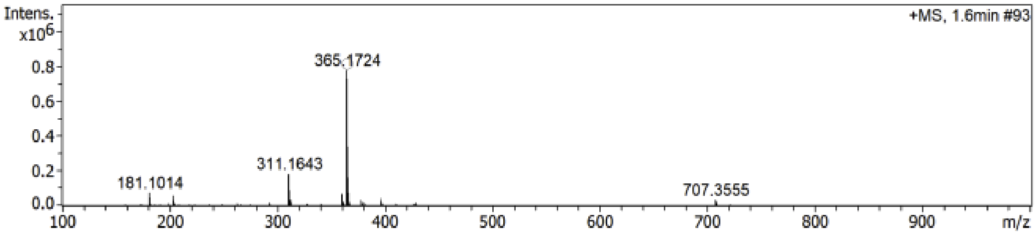

Meas. m/z	#	Ion Formula	m/z	err [ppm]	mSigma	# mSigma	Score	rdb	e⁻ Conf	N-Rule
365.1724	1	C21H26NaO4	365.1723	-0.2	6.9	1	100.00	9.0	even	ok

Figure S31. HRMS results for compound **8**.

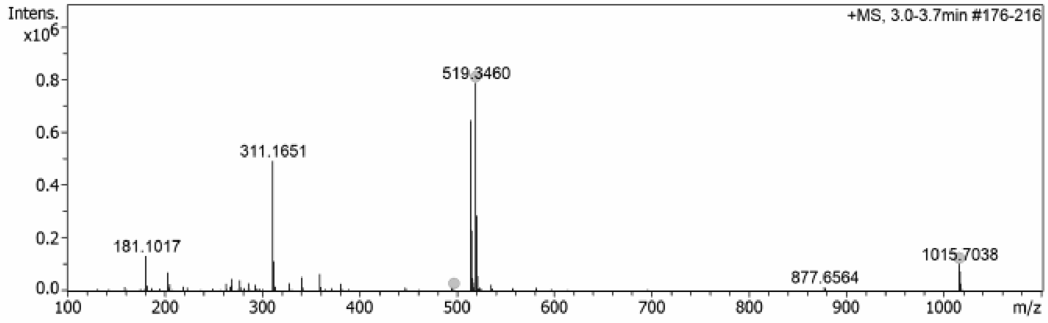

Meas. m/z	#	Ion Formula	m/z	err [ppm]	mSigma	# mSigma	Score	rdb	e⁻ Conf	N-Rule
497.3635	1	C32H49O4	497.3625	-1.9	39.9	1	100.00	9.0	even	ok
519.3460	1	C32H48NaO4	519.3445	-2.8	4.7	1	100.00	9.0	even	ok
1015.7038	1	C64H96NaO8	1015.6997	-4.0	7.9	1	100.00	17.0	even	ok

Figure S32. HRMS results for compound **9**.

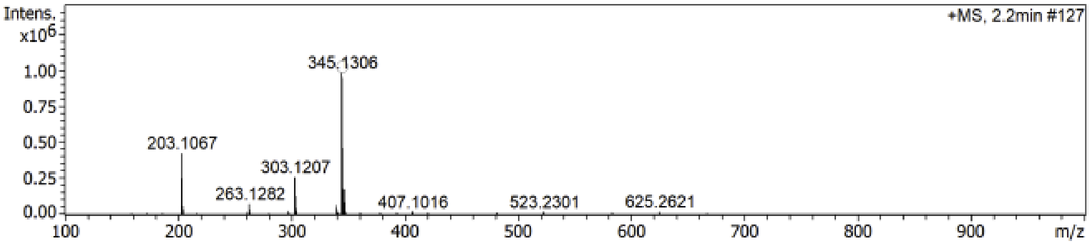

Meas. m/z	#	Ion Formula	m/z	err [ppm]	mSigma	# mSigma	Score	rdb	e⁻ Conf	N-Rule
345.1306	1	C17H22NaO6	345.1309	0.8	1.8	1	100.00	7.0	even	ok

Figure S33. HRMS results for compound **10**.

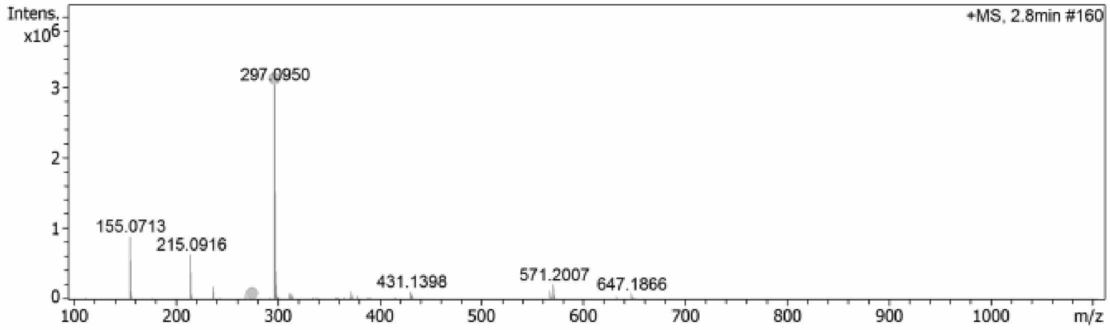

Meas. m/z	#	Ion Formula	m/z	err [ppm]	mSigma	# mSigma	Score	rdb	e⁻ Conf	N-Rule
275.1126	1	C12H19O7	275.1125	-0.3	n.a.	n.a.	n.a.	4.0	even	ok
297.0950	1	C12H18NaO7	297.0945	-1.8	0.1	1	100.00	4.0	even	ok

Figure S34. HRMS results for compound **11**.

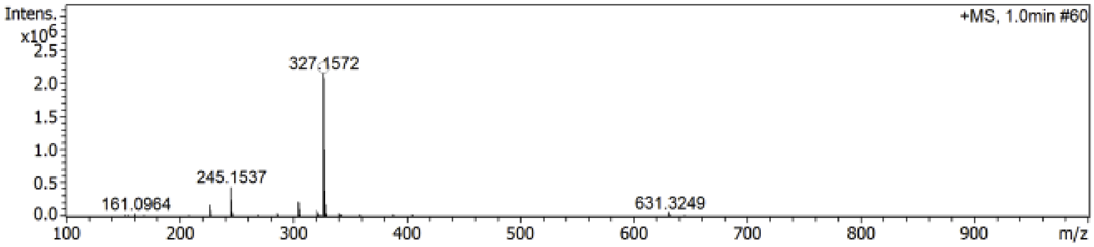

Meas. m/z	#	Ion Formula	m/z	err [ppm]	mSigma	# mSigma	Score	rdb	e⁻ Conf	N-Rule
327.1572	1	C18H24NaO4	327.1567	-1.5	5.8	1	100.00	7.0	even	ok

Figure S35. HRMS results for compound **12**.

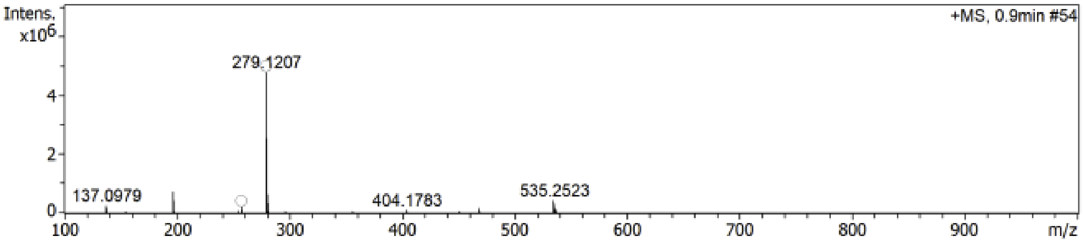

Meas. m/z	#	Ion Formula	m/z	err [ppm]	mSigma	# mSigma	Score	rdb	e¯ Conf	N-Rule
257.1386	1	C13H21O5	257.1384	-1.0	11.7	1	100.00	4.0	even	ok
279.1207	1	C13H20NaO5	279.1203	-1.6	6.5	1	100.00	4.0	even	ok

Figure S36. HRMS results for compound **13**.

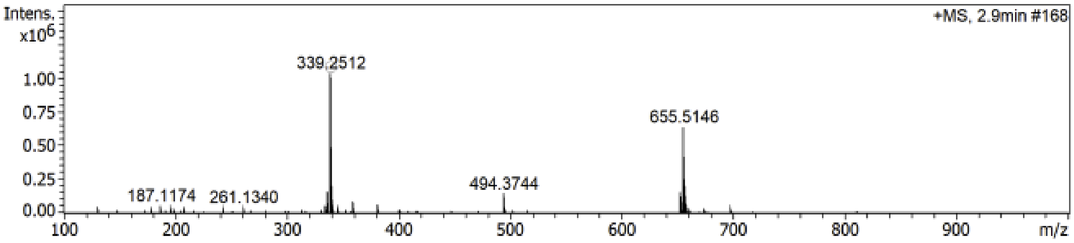

Meas. m/z	#	Ion Formula	m/z	err [ppm]	mSigma	# mSigma	Score	rdb	e¯ Conf	N-Rule
339.2512	1	C18H36NaO4	339.2506	-1.8	1.8	1	100.00	1.0	even	ok

Figure S37. HRMS results for compound **1**.

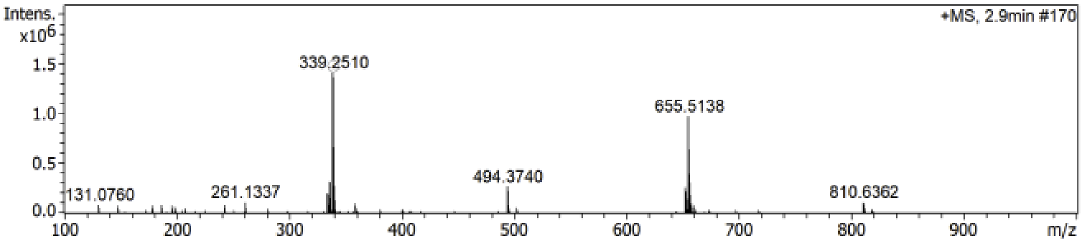

Meas. m/z	#	Ion Formula	m/z	err [ppm]	mSigma	# mSigma	Score	rdb	e¯ Conf	N-Rule
339.2510	1	C18H36NaO4	339.2506	-1.1	0.7	1	100.00	1.0	even	ok

Figure S38. HRMS results for compound **2**.

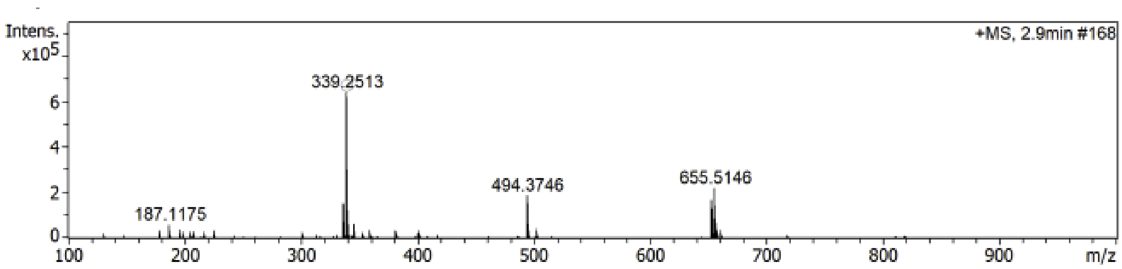

Meas. m/z	#	Ion Formula	m/z	err [ppm]	mSigma	# mSigma	Score	rdb	e^- Conf	N-Rule
339.2513	1	C18H36NaO4	339.2506	-2.0	2.2	1	100.00	1.0	even	ok

Figure S39. HRMS results for compound **3**.

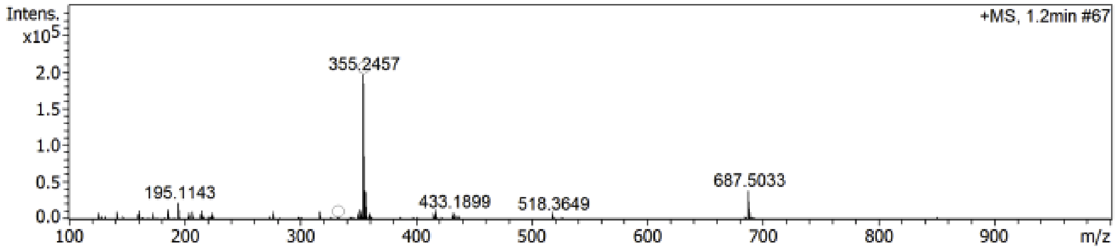

Meas. m/z	#	Ion Formula	m/z	err [ppm]	mSigma	# mSigma	Score	rdb	e^- Conf	N-Rule
333.2634	1	C18H37O5	333.2636	0.6	27.8	1	100.00	1.0	even	ok
355.2457	1	C18H36NaO5	355.2455	-0.5	1.0	1	100.00	1.0	even	ok

Figure S40. HRMS results for compound **4**.

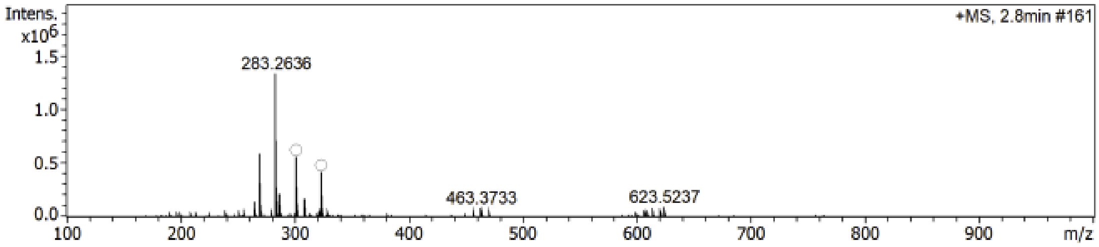

Meas. m/z	#	Ion Formula	m/z	err [ppm]	mSigma	# mSigma	Score	rdb	e^- Conf	N-Rule
301.2742	1	C18H37O3	301.2737	-1.7	8.2	1	100.00	1.0	even	ok
323.2563	1	C18H36NaO3	323.2557	-1.8	1.6	1	100.00	1.0	even	ok

Figure S41. HRMS results for compound **5**. The major peak corresponds to [M-H_2O].

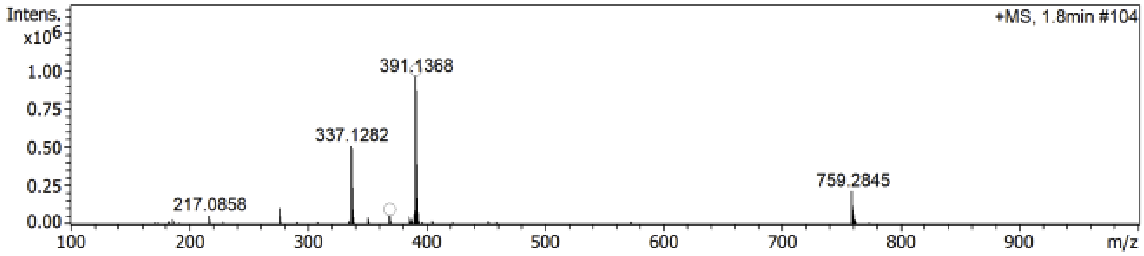

Meas. m/z	#	Ion Formula	m/z	err [ppm]	mSigma	# mSigma	Score	rdb	e⁻ Conf	N-Rule
369.1550	1	C18H25O8	369.1544	-1.6	5.3	1	100.00	7.0	even	ok
391.1368	1	C18H24NaO8	391.1363	-1.1	0.7	1	100.00	7.0	even	ok

Figure S42. HRMS results for compound **S2**.

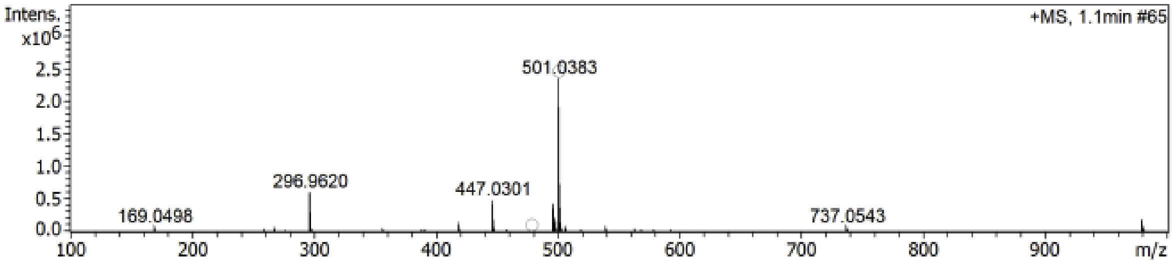

Meas. m/z	#	Ion Formula	m/z	err [ppm]	mSigma	# mSigma	Score	rdb	e⁻ Conf	N-Rule
479.0557	1	C18H24IO7	479.0561	0.8	n.a.	1	100.00	10.0	even	ok
501.0383	1	C18H23INaO7	501.0381	-0.4	2.7	1	100.00	10.0	even	ok

Figure S43. HRMS results for compound **S3**.

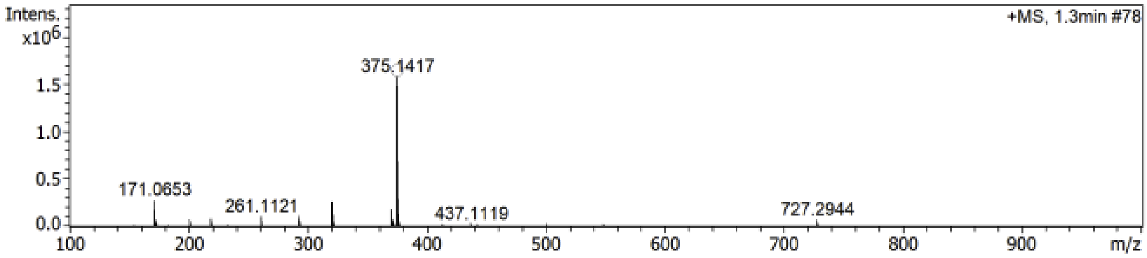

Meas. m/z	#	Ion Formula	m/z	err [ppm]	mSigma	# mSigma	Score	rdb	e⁻ Conf	N-Rule
375.1417	1	C18H24NaO7	375.1414	-0.8	1.3	1	100.00	7.0	even	ok

Figure S44. HRMS results for compound **S4**.

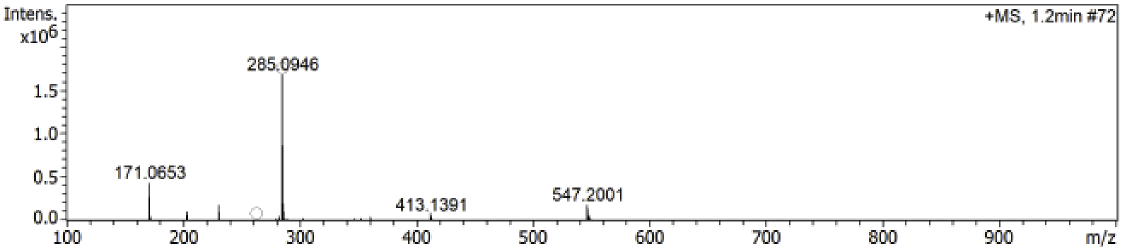

Meas. m/z	#	Ion Formula	m/z	err [ppm]	mSigma	# mSigma	Score	rdb	e⁻ Conf	N-Rule
263.1127	1	C11H19O7	263.1125	-0.6	30.7	1	100.00	3.0	even	ok
285.0946	1	C11H18NaO7	285.0945	-0.4	0.9	1	100.00	3.0	even	ok

Figure S45. HRMS results for compound **S5**.

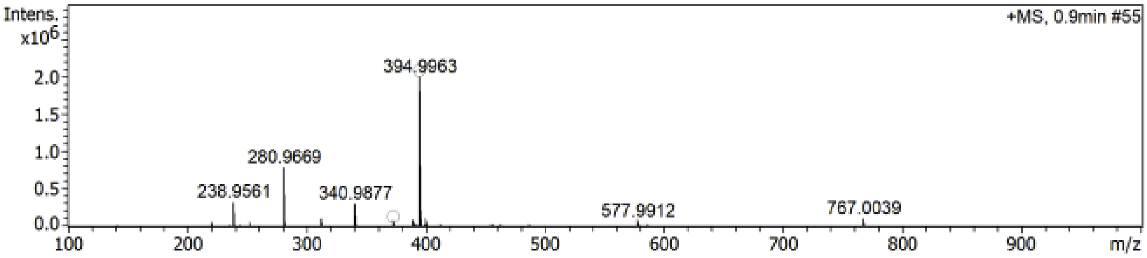

Meas. m/z	#	Ion Formula	m/z	err [ppm]	mSigma	# mSigma	Score	rdb	e⁻ Conf	N-Rule
373.0142	1	C11H18IO6	373.0143	0.3	11.2	1	100.00	6.0	even	ok
394.9963	1	C11H17INaO6	394.9962	-0.3	2.1	1	100.00	6.0	even	ok

Figure S46. HRMS results for compound **S6**.

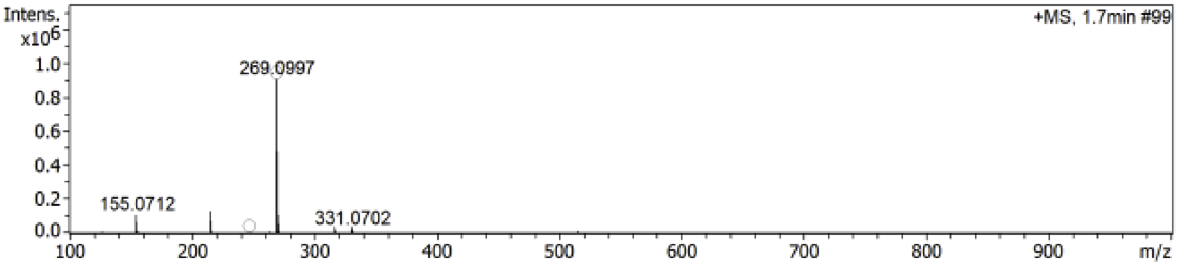

Meas. m/z	#	Ion Formula	m/z	err [ppm]	mSigma	# mSigma	Score	rdb	e⁻ Conf	N-Rule
247.1180	1	C11H19O6	247.1176	-1.6	n.a.	1	100.00	3.0	even	ok
269.0997	1	C11H18NaO6	269.0996	-0.4	0.7	1	100.00	3.0	even	ok

Figure S47. HRMS results for compound **S7**.
